# Identifying early metabolite markers of successful graft union formation in grapevine

**DOI:** 10.1093/hr/uhab070

**Published:** 2022-01-19

**Authors:** Grégoire Loupit, Josep Valls Fonayet, Sylvain Prigent, Duyen Prodhomme, Anne-Sophie Spilmont, Ghislaine Hilbert, Céline Franc, Gilles De Revel, Tristan Richard, Nathalie Ollat, Sarah Jane Cookson

**Affiliations:** 1 EGFV, University Bordeaux, Bordeaux Sciences Agro, INRAE, ISVV, F-33882 Villenave d'Ornon, France; 2 Bordeaux Metabolome Facility, MetaboHUB, PHENOME-EMPHASIS, Centre INRAE de Nouvelle Aquitaine - Bordeaux, av Edouard Bourlaux, 33140 Villenave d’Ornon, France; 3 University Bordeaux, Unité de recherche Œnologie, EA 4577, USC 1366 INRAE, ISVV, F33882 Villenave d’Ornon, France; 4 INRAE, University Bordeaux, UMR BFP, 33882 Villenave d’Ornon, France; 5 Institut Français de la Vigne et du Vin, Domaine de l’Espiguette, 30240 Le Grau-du-Roi, France

## Abstract

Grafting is an important horticultural technique used for many crop species. However, some scion/rootstock combinations are considered as incompatible due to poor graft union formation and subsequently high plant mortality. The early identification of graft incompatibility could allow the selection of non-viable plants before planting and would have a beneficial impact on research and development in the nursery sector. In general, visible phenotypes of grafted plants (size, root number, etc.) are poorly correlated with grafting success, but some studies have suggested that some polyphenols could be used as markers of graft incompatibility several months or years after grafting. However, much of the previous studies into metabolite markers of grafting success have not included all the controls necessary to unequivocally validate the markers proposed. In this study, we quantified 73 primary and secondary metabolites in nine hetero-grafts and six homo-grafted controls 33 days after grafting at the graft interface and in both the scion and rootstock woody tissues. Certain biomarker metabolites typical of a high stress status (such as proline, GABA and pallidol) were particularly accumulated at the graft interface of the incompatible scion/rootstock combination. We then used correlation analysis and generalized linear models to identify potential metabolite markers of grafting success measured one year after grafting. Here we present the first attempt to quantitatively predict graft compatibility and identify marker metabolites (especially asparagine, *trans*-resveratrol, *trans*-piceatannol and α-viniferin) 33 days after grafting, which was found to be particularly informative for homo-graft combinations.

## Introduction

In horticulture, grafting is a common vegetative propagation technique, allowing us to combine two different genotypes together to generate one plant. The mechanisms involved in graft union formation are complex and include the formation of a callus and new vascular tissues between the two genotypes. However, despite many studies and use of grafting for millennia, the mechanisms underlying the cohabitation of the two genotypes of grafted plants are still poorly understood [1].

The commercial use of grafting depends upon the degree of graft compatibility, i.e. the ability of the assembled scion/rootstock to form and sustain a successful graft union. Graft incompatibility is often characterized by anatomical irregularities at the graft interface, which induce mechanical weakness and subsequently death of plants. Graft incompatibility can occur soon after grafting, reducing grafting success in nurseries, or can appear several years after planting in the field (this delay may be only in the appearance of incompatibility symptoms that have been progressing, unobserved, since shortly after the grafting was performed). These incompatibilities can be due to the presence of a pathogen or to genetic distance, but poor grafting success can be also due to graft quality or climatic conditions during the growth [2]. The significant delay in the appearance of incompatibility symptoms renders the evaluation and transfer of new genotypes to industry time-consuming, expensive, and laborious.

In viticulture, after grafting, the grafts produced are planted in the nursery for one year, and then up-rooted and marketable grafts are selected. Many plants will not be considered marketable due to too insufficient root and/or shoot growth and/or problems with the development of callus at the graft interface; the proportion of marketable versus non-marketable plants gives an indication of grafting success. Graft incompatibility is relatively rare once grapevines have been planted in the vineyard, but of the 233.5 million plants grafted in France in 2017, only 155–165 million plants were commercialized [3], i.e. 69% of production and this is mainly due to failure to develop a successful graft union (personal communication, Pascal Bloy, IFV). To date, predicting poor grafting success using visual indicators or phenotypic measurements has not been conclusive. In grapevine, only one weak correlation between the stem diameter at the graft zone and the grafting success at 21 days after grafting (DAG) was identified, but the reliability of this criterion is questionable [4]. Therefore, it appears necessary to find other markers that can be correlated with the grafting success rate.

The accumulation of secondary compounds at the graft interface has long been described as an explanation of incompatibility mechanisms [5, 6]. The identification of metabolic markers linked to incompatibility has been studied in several species, such as pear, apricot or olive [7–9], but this was often done several months or years after grafting and did not allow the early prediction of grafting success. Furthermore, there are frequently control samples missing in many studies which make conclusions difficult to interpret.

In grapevine, the concentration of phenolic compounds at the graft interface three years after grafting was compared between two clones of *Vitis vinifera* L. cv. “Touriga Nacional”, which have different degrees of grafting success [10]. Gallic acid, epicatechin and catechin were found in higher concentrations at the graft interface of the more incompatible scion/rootstock combination at the rooting stage, and sinapic acid was at high concentrations in the incompatible scion/rootstock combination in all tissues at the end of the year in the nursery; these metabolites were described as potential metabolic markers of graft incompatibility [10]. Similarly, two studies have compared the metabolite profile of the scion, rootstock and graft interface of two different clones of *V. vinifera* cv. Syrah known to die back in the vineyard after grafting with certain rootstock genotypes and showed that the concentration of gallic acid was higher, and ferulic and sinapic acid was lower in the incompatible scion genotype [11, 12]. However, as homo-grafted controls were missing from these studies, it is impossible to know whether the differences are due to grafting with a non-compatible genotype or due to just the differences in the wounding response of the two clones being compared. In general, identifying reliable and robust graft incompatibility markers remains challenging [13].

The aim of this study was to identify marker metabolites that could be used to predict grafting success in grapevine; the objective was to graft different scion/rootstock combinations together, to analyze metabolites (24 primary and 49 secondary metabolites) in the scion, graft interface and rootstock tissues 33 DAG and quantify grafting success after one year in the nursery. These compounds were selected based on the literature [13]. The different hetero-grafts studied were selected to obtain differences in grafting success rates and graft development. *V. vinifera* cv. Merlot (MN)/*Vitis berlandieri x V. riparia* cv. Selection Oppenheim 4 (SO4) and *V. vinifera* cv. Ugni Blanc (UB)/*V. berlandieri x V. riparia* cv. Rességuier Sélection Birolleau 1 (RSB1) are very commonly produced by wine nurseries (6 and 5.3 million plants were produced in 2017 in France respectively) [3]. However, unlike MN/SO4 which is highly graft compatible, UB/RSB1 is often associated with a very low grafting success, and more generally, RSB1 is described as a rootstock with a moderate grafting capacity [14]. A third scion/rootstock combination, *V. vinifera* cv. Négrette (NG)/*V. berlandieri x V. rupestris* cv. Ruggeri (140Ru) was studied because it often has developmental problems producing large calluses and bulging graft unions and/or bad connections between the scion and rootstock that can result in dieback in the vineyard a few years after plantation (personal communication, Olivier Yobrégat, IFV) [14]. The leaves of these plants often redden during the autumn allegedly due to poor vascular connections between the rootstock and the scion [14]. In our experiment, all the three scion cultivars, MN, UB and NG were grafted with each of the three rootstocks SO4, RSB1 and 140Ru (along with their corresponding homo-grafted controls).

## Results

The graft combinations used in our study had very variable grafting success rates (ranging from 20.8% for UB/RSB1 to 77.1% for 140Ru/140Ru) (Table 1). Interestingly in most cases, the grafting success of the hetero-graft was between that of two corresponding homo-graft controls except in the case of scion/rootstock combinations containing RSB1: the grafting success was lower in the hetero-grafts UB/RSB1 and higher for MN/RSB1 and NG/RSB1 than the corresponding homo-graft controls. In case of UB/RSB1, we considered this hetero-graft as incompatible due to a lower grafting success (20.8%) compared to its corresponding homo-graft controls (36.6% for UB/UB and 25.1% for RSB1/RSB1).

**Table 1:** The scion/rootstock combinations used in this study and grafting success rate, Vitis International Variety Catalogue numbers given in brackets

Abbreviation	Scion genotype	Rootstock genotype	% of grafting success
*Hetero-grafts*			
MN/140Ru	*Vitis vinifera* cv. Merlot Noir clone 343 (7657)	*Vitis berlandieri* x *V. rupestris* cv. 140 Ruggeri clone 265 (10351)	**53.5**
NG/140Ru	*V. vinifera* cv. Négrette clone 581 (8452)	*V. berlandieri* x *V. rupestris* cv. 140 Ruggeri clone 265 (10351)	**50.0**
UB/140Ru	*V. vinifera* cv. Ugni Blanc clone 483 (17351)	*V. berlandieri* x *V. rupestris* cv. 140 Ruggeri clone 265 (10351)	**56.6**
MN/SO4	*V. vinifera* cv. Merlot Noir clone 343 (7657)	*V. berlandieri* x *V. riparia* cv. Sélection Oppenheim 4 clone 762 (11473)	**63.6**
NG/SO4	*V. vinifera* cv. Négrette clone 581 (8452)	*V. berlandieri* x *V. riparia* cv. Sélection Oppenheim 4 clone 762 (11473)	**41.8**
UB/SO4	*V. vinifera* cv. Ugni Blanc clone 483 (17351)	*V. berlandieri* x *V. riparia* cv. Sélection Oppenheim 4 clone 762 (11473)	**59.8**
NG/RSB1	*V. vinifera* cv. Négrette clone 582 (8452)	*V. berlandieri* x *V. riparia* cv. Rességuier Sélection Birolleau 1 clone 141 (4028)	**42.5**
MN/RSB1	*V. vinifera* cv. Merlot Noir clone 343 (7657)	*V. berlandieri* x *V. riparia* cv. Rességuier Sélection Birolleau 1 clone 141 (4028)	**44.8**
UB/RSB1	*V. vinifera* cv. Ugni Blanc clone 482 (17351)	*V. berlandieri* x *V. riparia* cv. Rességuier Sélection Birolleau 1 clone 141 (4028)	**20.8**
*Homo-grafts*			
MN/MN	*V. vinifera* cv. Merlot Noir clone 343 (7657)	*V. vinifera* cv. Merlot Noir clone 343 (7657)	**39.4**
NG/NG	*V. vinifera* cv. Négrette clone 582 (8452)	*V. vinifera* cv. Négrette clone 663 (8452)	**30.9**
UB/UB	*V. vinifera* cv. Ugni Blanc clone 482 (17351)	*V. vinifera* cv. Ugni Blanc clone 479 (17351)	**36.6**
140Ru/140Ru	*V. berlandieri* x *V. rupestris* cv. 140 Ruggeri clone 265 (10351)	*V. berlandieri* x *V. rupestris* cv. 140 Ruggeri clone 265 (10351)	**77.1**
SO4/SO4	*V. berlandieri* x *V. riparia* cv. Sélection Oppenheim 4 clone 762 (11473)	*V. berlandieri* x *V. riparia* cv. Sélection Oppenheim 4 clone 762 (11473)	**73.3**
RSB1/RSB1	*V. berlandieri* x *V. riparia* cv. Rességuier Sélection Birolleau 1 clone 141 (4028)	*V. berlandieri* x *V. riparia* cv. Rességuier Sélection Birolleau 1 (clone 141 (4028)	**25.1**

## Primary metabolism is reprogrammed at the graft interface.

**Figure 1:**
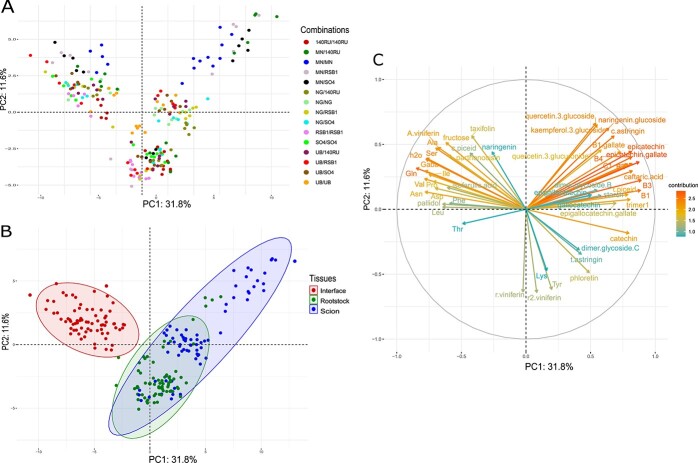
Principal component analysis (PCA) of metabolite concentration from all scion/rootstock combination and all tissues analyzed 33 days after grafting (n = 5). PCA score plots showing the different individuals, (A) colored by scion/rootstock combination and (B) colored by the tissue analyzed. (C) PCA loadings plot of the most 50 contributing metabolites. The color and the size of the arrows indicate the contribution strength of each metabolite.

To characterize the metabolite profiles of the nine hetero- and six homo-grafts studied, metabolites were quantified in the scion wood, graft interface and rootstock wood 33 DAG. Amino acids, starch, total proteins and soluble sugars were quantified as well as 49 secondary metabolites that were identified by HPLC-QqQ. The main secondary metabolites quantified were stilbenes (7 monomers, 12 dimers, 2 trimers and 4 tetramers). Overall, the metabolic profile of the graft interface was very different compared to scion and rootstock (Figure 1B and 1C). The first two principal components (PCs) explained 43.4% of the total inertia with an almost perfect separation between the graft interface and the surrounding woody scion and rootstock tissues (Figure 1B and 1C). Primary metabolites discriminating the graft interface from the scion and rootstock tissue were found mainly on the negative side of PC1, strongly associated with, globally, a higher concentration of amino acids such as Val, GABA, Ile, Ser, Pro, Ala, Gln, Asn, Asp, Leu and Phe, but also in soluble sugars, especially fructose. The concentration of total protein was mostly higher, and the concentration of starch was lower in the graft interface relative to the surrounding scion and rootstock tissues. Concerning secondary metabolites, PC1 was strongly correlated with compounds like isoferulic acid, pallidol, parthenocisin and α-viniferin. However, all flavanols and flavonols were globally at lower concentrations in the graft interface than the surrounding woody tissues (Figure 1B and 1C).

The scion and rootstock wood samples tended to cluster according to the genotype of the tissue studied. A separation was found along PC2 between MN, UB and NG and non-vinifera genotypes; RSB1, SO4 and 140Ru were on the negative side of PC2 which correlated with compounds like Tyr, Lys, *trans*-ω-viniferin, and *trans-*ε-viniferin as well as two tetramers of stilbenes (r-viniferin et r2-viniferin). Conversely, MN was on the positive side of PC2 and PC1, and strongly correlated with high concentrations of flavanols and some flavonols, especially epicatechin, flavanol dimers, quercetin-3-glucoside, naringenin-glucoside, and kaempferol-3-glucoside (Figure 1A and 1C).

## The metabolome at the graft interface, scion and rootstock

In addition to the more general metabolome differences between the scion, rootstock and graft interface tissues obtained from the PC analysis, details of the primary metabolite profile of the different scion/rootstock combinations are shown in Supplementary figure 1 (further details are given in Supplementary Tables 1-15). For example, Val was generally accumulated at the graft interface and this accumulation is particularly high in scion/rootstock combinations containing RSB1 and SO4. Similarly, Gly and Asn are often at particularly high concentrations in scion/rootstock combinations containing RSB1 and SO4 respectively (Supplementary figure 1). The scion/rootstock combinations in Supplementary figure 1 are ordered by grafting success rate, the hetero-grafts with the lowest grafting success (such as UB/RSB1 and MN/RSB1) were associated with the highest concentrations of the free amino acids Phe, Ser, Pro, Leu, Ala, Asp, Pro and Ile at the graft interface. In addition, it was interesting to note that RSB1/RSB1 homograft had the lowest concentration of starch and soluble sugars in wood tissues, but had similar concentrations compared to other combinations (such as NG/NG or NG/RSB1) at the graft interface (Supplementary figure 1). The concentration of fructose at the graft interface was particularly high in the hetero-grafts with high grafting success (namely MN/SO4 and UB/SO4) and higher than their corresponding homo-graft controls.

The quantification of secondary metabolites in the scion, rootstock and graft interface at 33 DAG showed significant differences between the different scion/rootstock combinations (Supplementary figure 2, Supplementary Table 1-15). Concerning stilbenes, MN had high concentrations of stilbene monomers (especially in *cis-*astringin) while the other genotypes had lower concentrations, especially RSB1/RSB1, which had the lowest concentration at the graft interface (Supplementary figure 2). However, regarding stilbene dimers, NG, RSB1 and SO4 had higher concentrations than 140Ru and MN. A significant accumulation at the graft interface was observed in stilbenes trimers except for NG hetero-grafts and for the RSB1 homo-grafts. Also, in homo-grafted controls, the accumulation of a stilbene dimer, parthenocisin, was found in higher concentration in *V. vinifera* homo-grafts compared to rootstock homo-grafts (Supplementary figure 2). Generally, no accumulation of flavanols at the graft interface was observed, but there were large genotype-specific differences (Supplementary figure 2). For example, non-*vinifera* woody tissues had lower epicatechin concentrations than MN, NG or UB. In addition, woody tissues of MN and NG had higher concentrations of gallocatechin, epigallocatechin, epigallocatechin gallate and B1 gallate compared to UB and non-*vinifera* genotypes. Several flavonols, phenolic acids, compounds such as naringenin and its glucosidic form, and taxifolin, belonging to the subfamilies of flavonoids, were also measured. In our data, total flavonol concentration was low and did not exceed 10 mg kg^−1^ (except for certain MN tissues). Despite these low concentrations, the accumulation of kaempferol 3-glucoside in NG/NG and UB/UB, and quercetin 3-glucoside in NG/NG, UB/UB, RSB1/RSB1, and NG/140Ru, was measured at the graft interface. Interestingly, a specific and strong accumulation of naringenin and taxifolin at the graft interface of MN hetero-grafts was found, contrary to MN/MN and non-*vinifera* homo-grafts (Supplementary figure 2), showing the impact of hetero-grafting on the metabolite profile of the graft interface. Four phenolic acids were identified and quantified; gallic acid was the most highly concentrated. However, this compound was found in very low concentration in UB tissues. No accumulation of total phenolic acids at the graft interface was shown, but there was an increase of isoferulic acid at the graft interface compared to scion and rootstock in all scion/rootstock combinations except for MN/MN, NG/NG and UB/UB (Supplementary figure 2).

## Certain biomarker metabolites identified in the incompatible combination

Because only UB/RSB1 had lower grafting success than its corresponding homo-graft controls, we analyzed the data to determine how UB/RSB1 differs from the other scion/rootstock combinations. For example, if we consider the percentage of water in the samples of the graft interface, the two homo-grafts UB/UB and RSB1/RSB1 had the highest percentages, but, the UB/RSB1 heterograft had a much lower percentage; the other hetero-grafts had intermediate or equivalent percentages with respect to their homo-grafted controls. Compared to the homo-grafted controls, UB/RSB1 had higher concentrations of total amino acids at the graft interface while the different hetero-grafts had concentrations that were between those of their corresponding homo-graft controls (data not shown). By focusing on UB/RSB1, a specific accumulation of GABA, Pro and Asp at the interface could be identified compared to homo-grafted controls and other hetero-grafts (Supplementary figure 1).

Concerning secondary metabolites, no specific accumulation could be observed at the graft interface of UB/RSB1 in comparison to its homo-grafted controls except for pallidol. In fact, this compound was found at a higher concentration at the graft interface of UB/RBS1 than any other scion/rootstock combinations studied (Supplementary figure 2). However, the accumulation of pallidol at the graft interface of MN/SO4, UB/140Ru and UB/SO4 could also be observed in comparison with their corresponding homo-graft controls.

## Stilbenes can predict grafting success well in homo-grafted controls.

**Figure 2:**
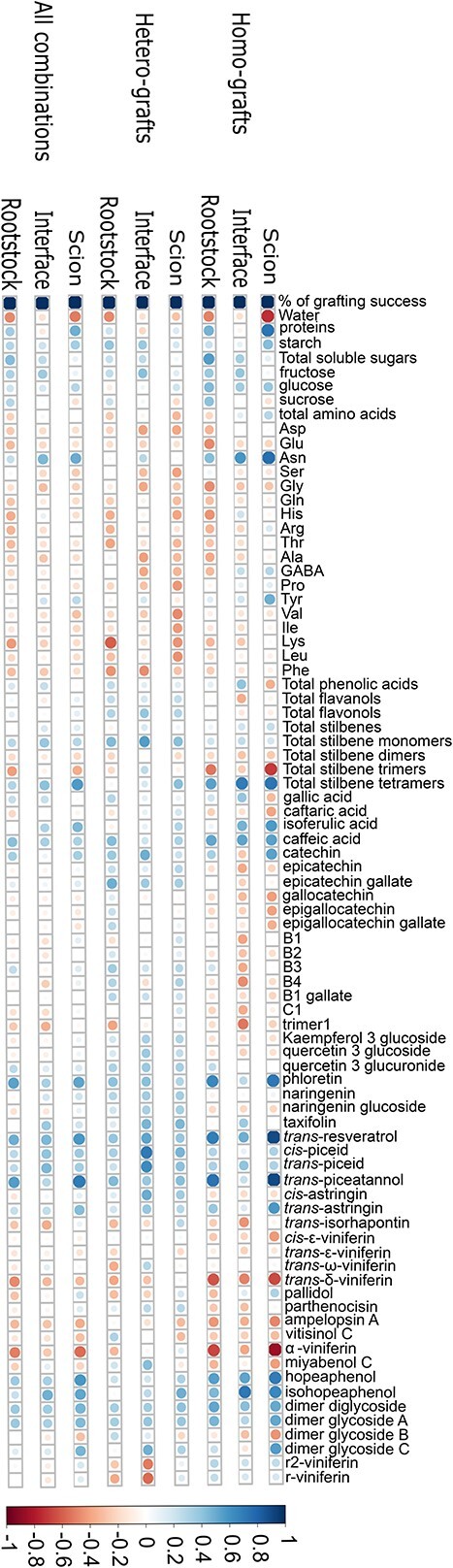
The Pearson correlation coefficient between percentage of grafting success (calculated 37 weeks after grafting, n = 200) and metabolite concentrations (quantified 33 days after grafting, using 5 pools of 5 plants) for all scion/rootstock combinations, only hetero-graft combinations, and only homo-graft combinations in the three different tissues studied. Positive correlations are colored in blue and negative correlation in red. The size and color intensities represent the correlation level.

To assess whether any metabolite concentration correlated with grafting success rate, correlation matrices were made between the different tissues (scion, graft interface and rootstock) and percentage of successful grafts produced; either the data from all scion/rootstock combinations were used or it was separated into either homo- or hetero-graft data (Figure 2). When considering all the dataset from all scion/rootstock combinations, it is not at the graft interface nor in the rootstock that we have found the most significant correlations, but in the scions. In scions, i.e. just above the graft interface, the strongest correlation (r^2^ = 0.48) between a metabolite concentration and grafting success was found for *t*-piceatannol.

When studying only the homo-graft dataset, the strongest correlation between grafting success and a metabolite concentration was found at the graft interface for stilbene total tetramers and for isohopeaphenol (r^2^ = 0.49 and 0.51 respectively). In rootstock tissue, the strongest correlation was between grafting success and *t*-piceatannol (r^2^ = 0.55). The correlations were highest in the scion tissue between grafting success and *t*-resveratrol, *t*-piceatannol (positively correlated) and α-viniferin (negatively correlated) concentrations (r^2^ = 0.79, 0.8 and 0.74 respectively) In addition, the percentage of water and total stilbene trimers were negatively correlated with the grafting success rate (r^2^ = 0.54 and 0.49 respectively) while the amount of total protein, and Asn, total stilbene tetramers and hopeaphenol concentrations were positively correlated (r^2^ = 0.52, 0.57, 0.53 and 0.49 respectively). (Figure 2).

On the contrary, no strong correlations between metabolite concentration and grafting success were found when only the hetero-graft dataset was used; the highest correlation found was at the graft interface for *c*-piceid (r^2^ = 0.5) (Figure 2).

**Figure 3:**
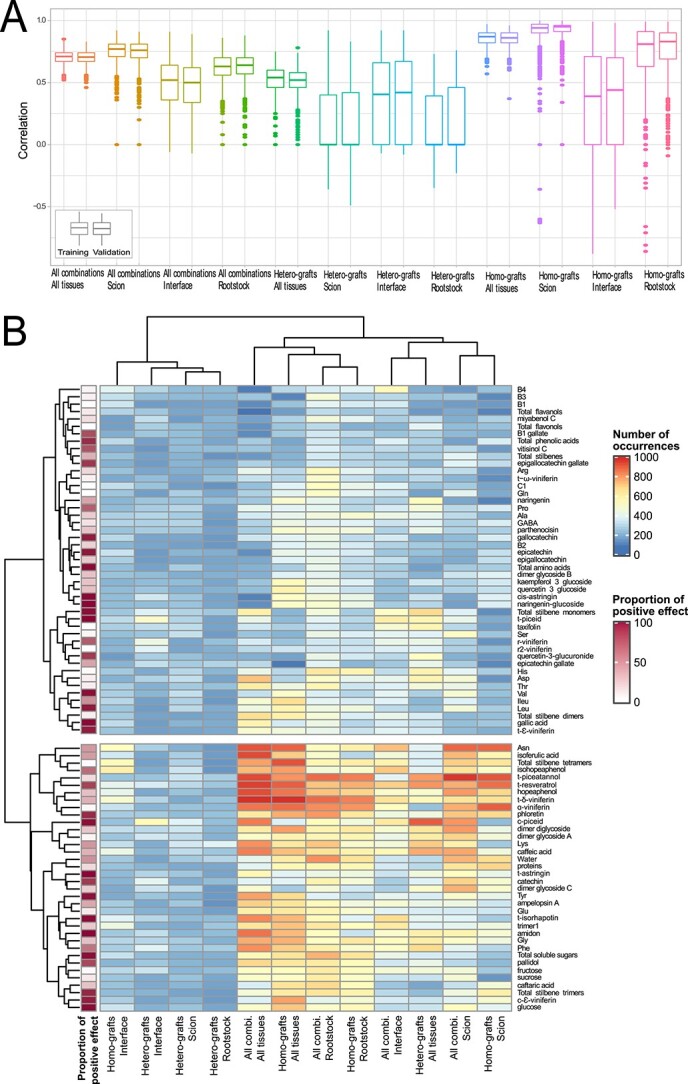
Correlation boxplots (A) between models created and quantified data, and heatmap of number of occurrences (B) for metabolites used for predicting the generalized linear models. Negative or positive correlations are indicated by the column of proportion of positives.

To further identify markers of grafting success, Generalized Linear Models (GLMs) were created to predict the percentage of grafting success using all the metabolomic data (primary and secondary metabolites). Two parameters were taken into these models, which were the correlation between the model created and real values with the grafting success, and the contribution of each compound to participate in model construction. Variables appearing the most times in the models were considered as reliable predictors. Models were created for all data combined (all combinations in all tissues), then separated into different datasets: scion, interface, or rootstock, using only homo-grafts or hetero-grafts combinations (Figure 3A). Additionally, the proportion of positive correlation for each compound found in all models was calculated (Figure 3B). Correlations between predicted and real values of grafting success showed the strongest correlation (> 80%) in homo-graft scion tissues (Figure 3A). The compounds found the most times in the model were *t*-resveratrol, *t*-piceatannol, α-viniferin as well as Asn (Figure 3B). In comparison with the different datasets used, stronger correlations were found for homo-grafts compared to hetero-grafts except at the graft interface where they were quite similar. In addition, correlations were also higher in the tissue of the scion, than in the rootstock or the graft interface (Figure 3A). Predictable variables in the different datasets were often stilbenes and some amino acids, whereas flavanols and flavonols were not found (Figure 3B).

## Discussion

Here we identified and quantified part of the metabolome of the graft interface and surrounding woody tissues at 33 DAG in different scion/rootstock combinations with the objective to identify markers of graft incompatibility and compounds correlated with grafting success. Grafting success is a complex trait to phenotype, in this study, grafts were considered successful if the shoot had developed and the stem had lignified, if roots (at least 3) were present and homogeneously distributed, and if the graft interface was resistant to a force being applied to it (“the thumb test”), which are the same criteria used in grapevine nurseries. As such, it is difficult to separate grafting success from rooting success (which is the ability to produce roots) in the first year in the nursery. Here we define graft incompatibility as a lower rate of plant survival of a hetero-graft in relation to its corresponding homo-graft controls: based on this criterion only UB/RSB1 is incompatible, but other scion/rootstock combinations studied here also showed large variation in grafting success rates. The scion/rootstock combination MN/SO4 had the highest grafting success in agreement with the known behavior of this scion/rootstock combination.

## Primary metabolites associated with grafting success in grapevine.

The analysis of primary metabolites revealed the high concentration of sugars and several amino acids like Gln, GABA, Ser, Ala, Asp, Pro, and Ile at the graft interface 33 DAG, which presumably comes from the mobilization of wood starch reserves. Cell proliferation for callus formation at the graft interface is one of the key steps to generate a successful graft union formation and is probably very energetically expensive. High concentrations of fructose were observed at the graft interface of highly compatible hetero-grafts, potentially suggesting a higher metabolic activity than homo-grafts and scion/rootstock combinations with lower grafting success. Low concentrations of starch were found in the wood and the graft interface of RSB1/RSB1, which suggests that the wood used for grafting had fewer starch reserves, which could explain the low soluble sugar concentrations in wood and its poor grafting success. However, this did not seem to have an impact on grafting success of RSB1 hetero-grafts. Furthermore, high levels of soluble sugars were found in MN/SO4 as well in UB/RSB1, which showed very different levels of grafting success suggesting that the mobilization of carbon reserves at the graft interface is not what limits graft union formation.

## Primary metabolism status as a marker of incompatibility

Some potential incompatibility markers were identified in the UB/RSB1 hetero-graft, such as a high accumulation of Asp, Pro and GABA at the graft interface. γ-aminobutyric acid is well known for its role in plant development [15]. In addition, both GABA and Pro accumulate in response to oxidative stress [16] and exogenous application of GABA and Pro has been shown to reduce stress induced oxidative damage [17, 18]. Aspartic acid is a central amino acid used to supply precursors to a large number of metabolic pathways, Asp, like GABA and Pro, is known to accumulate in response to stress [19]. The accumulation of GABA, Pro and Asp is seen in response to wounding of tomato cotyledons [20] and the over-expression of *WOUND INDUCED DEDIFFERENTIATION1 (WIND1)*, a gene involved in wound induced cellular reprogramming, triggers the accumulation of both GABA and Pro [21]. Proline and GABA have both been shown to improve callus formation *in vitro* [22–24]. This could suggest that both Pro and GABA are important for graft union formation potentially by reducing oxidative stress damage. The accumulation of Pro and GABA at the graft interface of UB/RSB1 could suggest that this combination has a higher stress status that the graft interfaces of the other scion/rootstock combinations and that the high accumulation of these amino acids could be markers of graft incompatibility.

## Few secondary metabolites were accumulated at the graft interface

Both grafting and wounding stimulate defense mechanisms and local secondary metabolite synthesis [13, 25, 26]. During graft union formation, phenolic compounds are potentially involved in various developmental and differentiation processes [1]. Surprisingly, in our data on grapevine, only few secondary metabolites were accumulated specifically at the graft interface (i.e. secondary metabolite concentrations were lower than the surrounding scion and rootstock tissues), whereas Prodhomme et al. (2019) identified the accumulation of 12 stilbenes 28 DAG at the graft interface of homo-grafts of *V. vinifera* cv. Cabernet sauvignon at 28 DAG. In our data, only MN/MN homo-graft had higher total stilbene concentration at the graft interface compared to scion and rootstock tissues. This could be due to the genotypes studied, or the different stratification methods used, in our study grafts were placed in boxes with 1 cm of water in the bottom, while Prodhomme et al. (2019) used humid sawdust filled boxes [27]. Another hypothesis is that this difference of accumulation was because callus was more developed at 33 than 28 DAG, so secondary metabolites were probably more diluted in the new callus tissue. A lower concentration of catechin and epicatechin at the graft interface compared to the surrounding woody tissues has also been found in all studies on grapevine grafting, whereas the accumulation of phenolic acids at the graft interface relative to the surrounding woody tissues appears to be more variable [10–12]. In our experiment, we did not observe an accumulation of gallic acid at the graft interface, but ferulic acid was accumulated at the graft interface of all scion/rootstock combinations except UB/UB and NG/NG.

## No flavanols, flavonols or phenolic acids were identified as markers of grafting success.

Previous studies have tried to identify markers of graft incompatibility in several species by targeting secondary metabolites. Several metabolites, in particular flavanols or phenolic acids, have been quantified in higher concentrations at the graft interface in incompatible combinations several months or years after grafting as reviewed by Loupit and Cookson, 2020 [13]. Studies in grapevine have shown that catechin, epicatechin, and gallic acid are found at different concentrations at the graft interface in scion/rootstock combinations with different degrees of compatibility [10–12]. This could suggest that epicatechin concentrations are potential marker metabolites in specific scion/rootstock combinations; however, in our study we did not find a strong association between these metabolites and grafting success. In different species, various studies have demonstrated an association between flavanols and certain cases of incompatibility, such as in pear [28] or apricot [9], but these compounds were quantified several years after grafting.

Finally, an accumulation of naringenin and taxifolin was found at the graft interface of MN hetero-grafts in comparison to rootstock homo-grafts and MN/MN. In addition to the antioxidant capacity of naringenin and taxifolin [29], naringenin has been shown to have an inhibitory effect on 4-Coumarate CoA ligase, potentially inhibiting the formation of lignin [30]. This could suggest that lignin formation is inhibited in MN hetero-grafts. However, the concentrations of flavonols and naringenin-glucoside were generally high in MN tissues in all scion/rootstock combinations, which could explain some of the high accumulation of naringenin and taxifolin at the graft interface of hetero-grafts of MN.

## Using stilbene concentrations to predict grafting success.

Stilbenes are well known in grapevine as participating in defense mechanisms thanks to their antioxidant and antifungal properties [31]. Concerning the secondary metabolites studied here, only the accumulation of one stilbene dimer (pallidol) was found at the graft interface of the incompatible UB/RSB1 combination. This compound, formed from two molecules of resveratrol, has an antioxidant capacity higher or equal than resveratrol and is considered as a selective singlet oxygen quencher [32, 33]. Pallidol has already been identified as being accumulated after wounding [26] and in response to downy mildew [34]. Like the high accumulation of GABA, Pro and Asp in UB/RSB1, the high concentrations of pallidol at the graft interface of UB/RSB1 may reflect higher stress status of the graft interface of this incompatible scion/rootstock combination.

In grapevine, to date no markers have been quantitatively related to grafting success across different scion/rootstock combinations. Previous studies have been limited to comparing either two [10] or four [11, 12] scion/rootstock combinations. In this study we used a larger range of scion/rootstock genotypes to attempt to quantitatively relate grafting success to a metabolite marker. The GLM made using data from all scion/rootstock combinations and all tissues gave a reasonable prediction of grafting success, which was further improved when only the scion tissues were analyzed. Why the metabolome of the scion has a greater predictive power than the rootstock is not clear, but it is generally assumed that the scion has a larger influence over graft union formation than the rootstock [2]. However, when the hetero-grafts were considered separately, the predictive power of the graft interface dataset was higher than of the scion and rootstock datasets considered separately, but the GLM build with all the data from all the tissues had the highest predictive power. Overall, we found that best dataset to predict the percentage of grafting success was when homo-grafted plants were considered separately, particularly in the scion tissue. This analysis showed that two stilbene monomers (*trans*-resveratrol and *trans*-piceatannol, which were positively related with grafting success) and one stilbene trimer α-viniferin, which was negatively related to grafting success) in the scion tissue just above the graft interface 33 DAG were the most important compounds used to predict grafting success. Presumably, finding markers of homo-grafting success is simpler than in hetero-grafts as the effect of the different genotypes on the grafting success is stronger as the phenotype is not affected by the interaction with another genotype of the graft.

**Figure 4:**
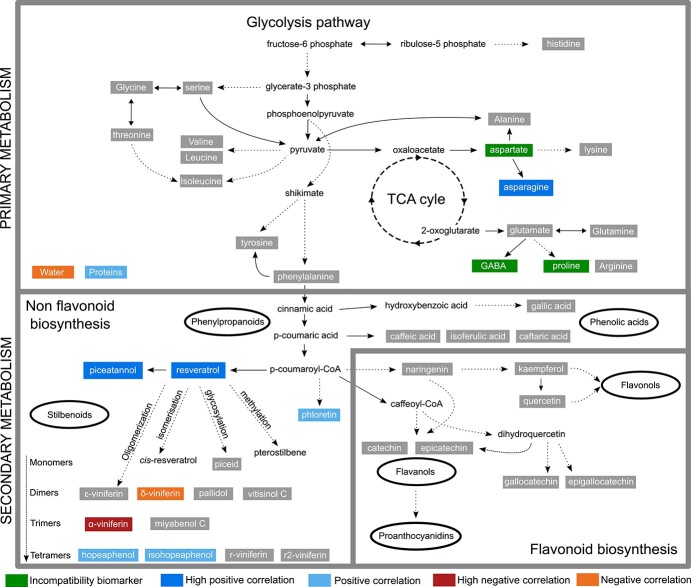
Schematic representation of primary and secondary metabolism in and around the graft interface of grapevine associated with grafting success. Compounds in green indicate an accumulation at the interface in the incompatible combination (UB/RBS1). Compounds in blue and red indicate the correlations found in homo-graft combinations between metabolite concentration and grafting success.

These two stilbene monomers and trimer are well known for their accumulation in response to abiotic stresses, particularly in the leaves, i.e. from 6 h after wounding for *trans*-resveratrol and from 96 h after wounding for α-viniferin [26], but also in grapevine canes after pruning in a context of valorization of viticulture by-products [35, 36]. As little is known about the function of individual stilbenes it is difficult to identify a potential role of these compounds and why their concentrations in the scion tissue are correlated with grafting success.

## Conclusion

Here we present an approach to identify metabolite markers of grafting success in grapevine; this is the first attempt to study so many metabolites across such many scion/rootstock combinations and to use correlation analysis and GLMs to interpret the data. We have tentatively identified some differences between the incompatible scion/rootstock combination UB/RSB1 and its homo-grafted controls and other scion/rootstock combinations, which highlighted the accumulation of amino acids known to be involved in stress responses at the graft interface. These results are summarized in Figure 4. Our research suggests that stilbenes can be used as a good early marker of grafting success to determine the short-term compatibility particularly in *V. vinifera* as well as in rootstock homo-grafts. However, the identification of markers of grafting success remains complex and may only be valid at a certain time point studied. This underlines the importance of doing a time course study to understand the kinetics of metabolome changes at the graft interface, such as changes in sugar and amino acid remobilization, and stilbene accumulation to select the best time point for grafting success prediction.

## Materials and methods

### Chemicals and standards

Standard amino acids (alanine (Ala), arginine (Arg), aspartate (Asp), asparagine (Asn), γ-aminobutyric acid (GABA), glycine (Gly), glutamate (Glu), glutamine (Gln), histidine (His), isoleucine (Ile), leucine (Leu), lysine (Lys), phenylalanine (Phe), proline (Pro), serine (Ser), threonine (Thr), tyrosine (Tyr) and valine (Val)) were purchased from Sigma.

Standards flavonols (quercetin 3-glucoside, quercetin 3-glucoronide and kaempferol 3-glucoside); phenolic acids (caffeic acid, gallic acid, caftaric acid and isoferulic acid); flavanols and procyanidins (catechin, epicatechin, epicatechin gallate, epigallocatechin, epigallocatechin gallate, B1, B2, C1); stilbenes (*trans*-piceid, *trans*-resveratrol and *trans-*piceatannol) and other compounds (phloretin, naringenin, naringenin glucoside and taxifolin) were purchased from Extrasynthesis (France). Stilbenes, for the majority, have been purified in the MIB laboratory (such as the monomers *trans-*astringin and *trans*-isorhapontin, the dimers *trans-*ε-viniferin, *trans*-ω-viniferin, *trans*-δ-viniferin, pallidol, parthenocissin A, vitisinol C and ampelopsin A, the trimers miyabenol C and α-viniferin, and the tetramers: hopeaphenol, isohopeaphenol, r2-viniferin and r-viniferin).

### Plant material and grafting procedure

Fifteen scion/rootstock combinations were omega bench grafted (n = 200); the list of different combinations is given in Table 1. The typical scion genotypes used in this study came from the Wine and Vine French Institute (IFV) based in Le Grau-du-Roi, France, and the typical rootstock genotypes came from Saint Jean de Maruejol (Gard, France). The material was virus-free. All the grafting steps were carried out at the IFV during spring 2019, after pruning the grapevine canes were stored in the fridge, then soaked in water at room temperature for rehydration 1 d before grafting. Scions were cut to a single bud and rootstocks were de-budded and cut to a length of about 28 cm. The grafting was realized using an Omega blade (Omega Star, Chauvin, France) on grapevine canes of approximately the same diameter on 28^th^ March 2019, then immediately dipped in melted wax, containing dichlorobenzoic acid (Staehler Rebwachs pro and Optimax (20–80), Chauvin) and placed in plastic crates. During the first 7 DAG, plants were kept at room temperature and then placed in callusing room (which gradually increased in temperature from 18 to 28°C over 4 d). Two cm of water (with 0.2% dichlorobenzoic acid and 40 mg L of copper sulfate) was added to the crates. Callusing lasted between 8 and 14 DAG depending on scion/rootstock combination. Just before plantation, grafted plants were again dipped in melted wax, containing indolebutyric acid (Rhizopon).

On 30^th^ April 2019, 33 DAG, 5 pools of 5 plants were harvested randomly. Pieces of about 1 cm in length were taken from the graft interface, and above (scion), and below (rootstock) the graft interface (without nodes) and directly snap frozen in liquid nitrogen. Samples were ground to powder in a ball mill (MM400 RETSCH) in liquid nitrogen at 30 Hz during 30 s and kept at −80°C.

On 16^th^ December 2019, grafting success was measured on all scion/rootstock combinations. After verifying that the plant has enough roots and a lignified stem, the “thumb test” was performed to test the strength of the callus. The success rate represents the percentage of plants that validate all these criteria.

### Soluble sugars, starch, and total protein analysis

The analysis was preceded by an ethanolic extraction as used by Hendriks et al., 2003 [37]. The pellet was used to analyze starch and total protein in the same way as Hendriks et al., 2003 [37] and Smith et al., 1985 [38]. The measurements were made in 96-well plates and the absorbance was read at 340 nm and 562 nm for starch and total protein, respectively. The supernatant allowed the analysis of glucose, fructose and sucrose as described in [39]. Starch, total proteins and soluble sugars are expressed as μmol eq glucose g^−1^ fresh weigh (FW), mg g^−1^ FW and μmol g^−1^ FW respectively.

### Free amino acids analysis

The ethanolic extract used for soluble sugars quantification was also used for quantification of free amino acids. Measures was made using an UltiMate 3000 ultra-HPLC system coupled with an FLD-3000 Fluorescence Detector after derivation with 6-aminoquinolyl-N-hydroxy-succinimidyl-carbamate (AccQTag derivatization reagent, Waters) with an excitation energy at 250 nm and a emission energy at 395 nm [40]. All results are expressed as μmol g^−1^ FW.

### Polyphenols analysis

About 250 mg of ground sample was extracted into 4 mL of methanol in an ultrasound bath for 15 min. Then, after centrifugation, the supernatants were collected and diluted with milli Q water (1:1, v:v). Analysis of phenolic compounds was carried out using a high performance liquid chromatography (Agilent Technology 1260 Infinity HPLC instrument equipped with an Agilent Poroshell 120 EC-C18 column (150 mm x 2.1 mm, 2.7 μm) thermostated at 35°C) coupled with a triple quadrupole mass spectrometer (Agilent Technologies 6430 triple quadrupole detector) as described and used by Loupit et al., 2020 [41] with some compounds added (further details are given in Supplementary Table 16). The concentration of standards ranging from 0.04 to 100 mg L^−1^ made it possible to build the calibration curve and quantify the different compounds. All compounds were quantified with their corresponding standards except for gallocatechin, procyanidin B3, B4, B1 gallate, *cis*-piceid, *cis*-ε-viniferin and *cis-*astringin, which were quantified as epigallocatechin, procyanidin B1, B2, B1, *trans*-piceid, *trans-*ε-viniferin and *trans-*astringin respectively. Also, one flavanol and four stilbene dimers have been identified with an attempted assignment, which are trimers1, and dimer diglycoside, dimer glycoside A, dimer glycoside B and dimer glycoside C, quantified as procyanidin B1 and *trans-*ε-viniferin respectively. All results are expressed as g kg^−1^ FW.

### Statistics

Principle component analysis (PCA), HeatMap, boxplot and statistical tests were made on the software R (version 4.0.4) and RStudio (version 1.2.5019) using ggplot2, gplot, FactoMineR and agricolae packages [42]. Furthermore, generalized linear models (GLM) were made on R (version 3.6.1) with glmnet package (version 3.0–2) [43]. Stratified sampling was performed to select randomly 80% of the samples to build the predictive models and 20% to test the quality of the prediction. This was performed 100 times for each dataset to cope with the random selection of the training and validation sets. 30-fold cross-validation was used in the construction of the models to decrease over-fitting, and mean square error was used to select the best models during the training step while testing for 1000 different penalization values (alpha) distributed between 0 and 1.

## Supplementary Material

Web_Material_uhab070

## Data Availability

Means and standard deviation for all combinations studied in all tissues are given in Supplementary Table 1 to 15. Data, with 5 five biological replicates, for analyzes of secondary metabolites, amino acids, soluble sugars, starch, proteins are available upon request.
