# Supplementary material for: Identifying early metabolite markers of successful graft union formation in grapevine
Source: Hortic Res. 2022 Jan 19;9:uhab070. doi: 10.1093/hr/uhab070 (PMC8881376; doi:10.1093/hr/uhab070)
Supplement: Web_Material_uhab070 [file web_material_uhab070.zip › supplementary figure 1 and 2.docx]

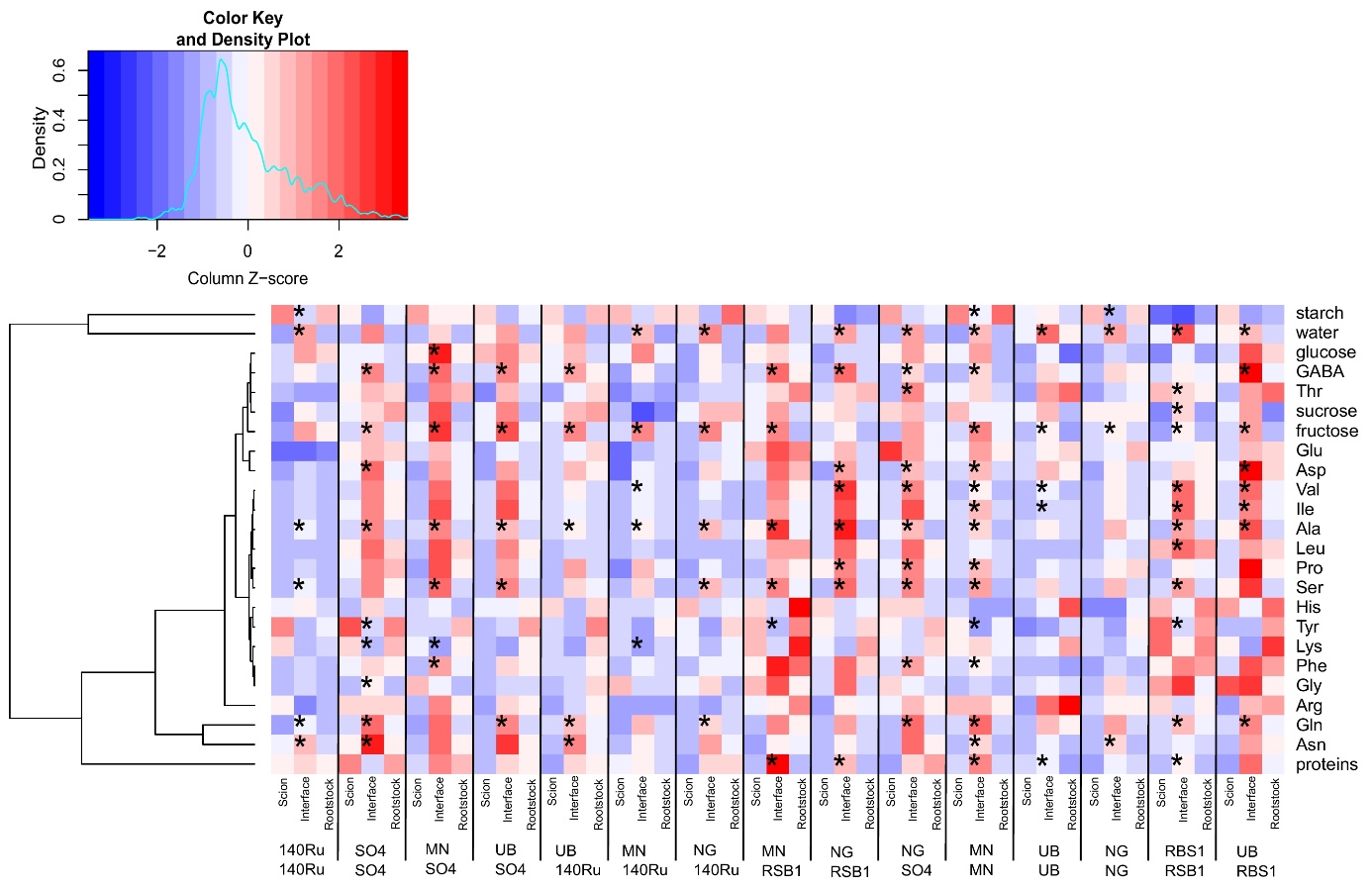
Supplementary Figure 1: Heatmap of the mean concentration of protein, amino acids, soluble sugars and starch, and the percentage of water in the different scion/rootstock combinations and tissues analyzed 33 days after grafting. Abbreviations used for the different genotypes given in Table 1. Stars indicates a significant difference between scion and graft interface, and between graft interface and rootstock, but no significant difference between scion and rootstock. Significance threshold set at p-value < 0.05.


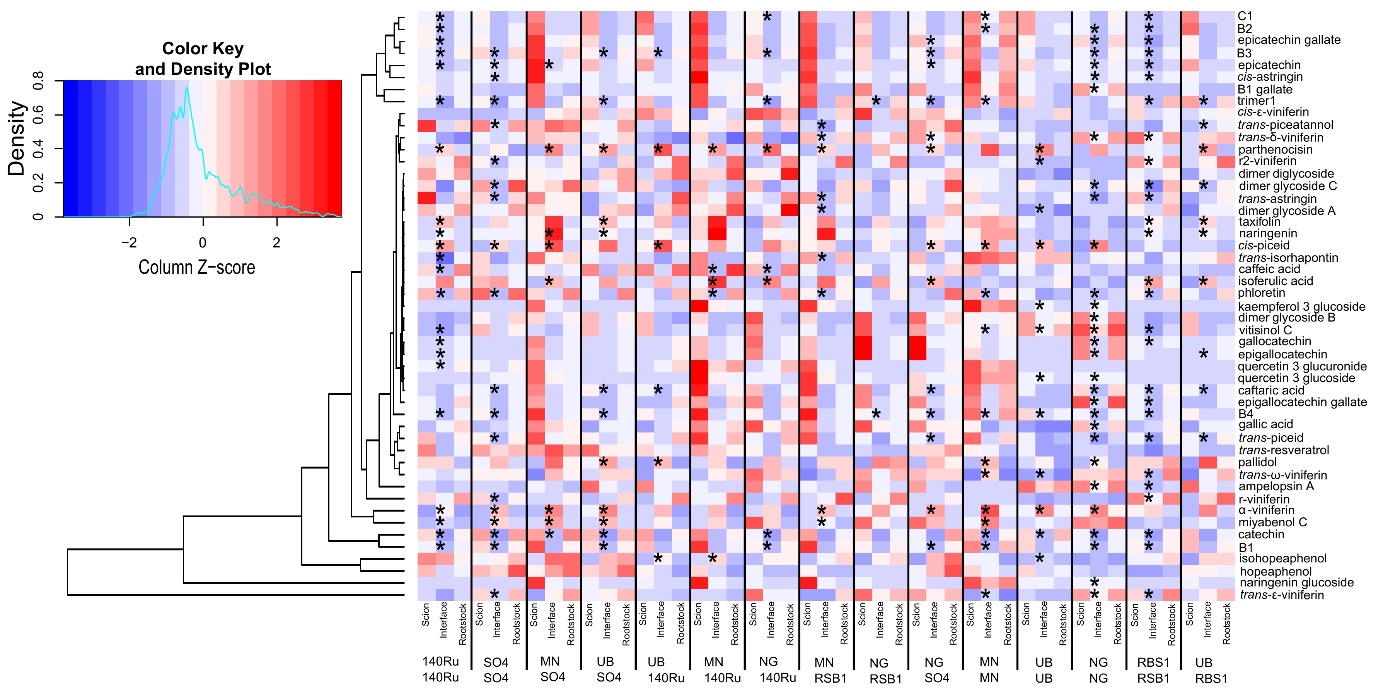
Supplementary Figure 2: Heatmap of the mean concentration of secondary metabolites in the different grafted scion/rootstock combinations and tissues analyzed 33 days after grafting. Abbreviations used for the different genotypes given in Table 1. Stars indicates a significant difference between scion and graft interface, and between graft interface and rootstock, but no significant difference between scion and rootstock. Significance threshold set at p-value < 0.05.
