# Supplementary material for: Identifying early metabolite markers of successful graft union formation in grapevine
Source: Hortic Res. 2022 Jan 19;9:uhab070. doi: 10.1093/hr/uhab070 (PMC8881376; doi:10.1093/hr/uhab070)
Supplement: Web_Material_uhab070 [file web_material_uhab070.zip › supplementary table 1-16.docx]

Supplementary Table 1: Metabolite concentration, protein content and percentage of water in the scion wood, graft interface and rootstock wood of homo-grafts of Vitis berlandieri x Vitis rupestris cv. 140 Ruggeri 33 days after grafting. Mean concentrations and standard deviation shown (n = 5). p-value of Wilcoxon-test between scion and interface tissues (S - I), scion and rootstock tissues (S - R), and interface and rootstock tissues (I - R). Stars indicates a significant difference between S – I and between I – R, but no significant difference between S - R. Significance threshold set at p-value < 0.05.

| **Variables** | **Mean** | | | **standard deviation** | | | ***p-value* Wilcoxon-test** | | |
| --- | --- | --- | --- | --- | --- | --- | --- | --- | --- |
|  | Scion | Interface | Rootstock | Scion | Interface | Rootstock | S - I | S - R | I - R |
| **% of water*** | 53.3459 | 71.3417 | 55.0657 | 0.7005 | 3.4854 | 1.3026 | 0.0119 | 0.0952 | 0.0119 |
| **starch*** | 99.2707 | 54.5091 | 83.1065 | 10.8165 | 9.0485 | 14.7529 | 0.0119 | 0.0952 | 0.0119 |
| **proteins** | 29.4356 | 30.7712 | 27.4925 | 6.3137 | 4.2136 | 4.8763 | 1 | 0.8214 | 0.6667 |
| **sucrose** | 0.4726 | 1.4436 | 1.0681 | 0.2849 | 1.3296 | 0.9124 | 0.4643 | 0.4643 | 0.6905 |
| **glucose** | 1.2756 | 2.0893 | 1.7437 | 0.2982 | 0.5869 | 0.5564 | 0.0952 | 0.2262 | 0.5476 |
| **fructose** | 0.6923 | 2.8987 | 1.8364 | 0.4552 | 0.6572 | 0.5224 | 0.0119 | 0.0119 | 0.0556 |
| *Amino acids* |  |  |  |  |  |  |  |  |  |
| **Ala*** | 0.2545 | 0.5097 | 0.2797 | 0.0857 | 0.0978 | 0.0662 | 0.0238 | 0.1508 | 0.0238 |
| **Arg** | 4.7819 | 2.3594 | 3.1853 | 0.7036 | 0.5775 | 1.3558 | 0.0238 | 0.2262 | 0.4206 |
| **Asn*** | 5.8908 | 12.2822 | 4.8934 | 0.8212 | 1.3621 | 1.9681 | 0.0119 | 0.4206 | 0.0119 |
| **Asp** | 1.7394 | 2.0566 | 1.7802 | 0.3784 | 0.3899 | 0.3751 | 0.631 | 1 | 0.631 |
| **GABA** | 1.0338 | 1.4796 | 0.9988 | 0.2234 | 0.2834 | 0.2745 | 0.0833 | 0.8413 | 0.0833 |
| **Gln*** | 5.6673 | 11.8361 | 6.9677 | 1.5696 | 1.4584 | 1.3534 | 0.0119 | 0.1508 | 0.0119 |
| **Glu** | 1.8082 | 1.9207 | 1.9958 | 0.4332 | 0.4401 | 0.4389 | 1 | 1 | 1 |
| **Gly** | 0.0113 | 0.0326 | 0.0138 | 0.015 | 0.013 | 0.0064 | 0.089 | 0.5296 | 0.089 |
| **His** | 0.6197 | 0.6904 | 0.4917 | 0.13 | 0.0906 | 0.1439 | 0.5476 | 0.4643 | 0.1667 |
| **Ile** | 0.163 | 0.2897 | 0.1687 | 0.0409 | 0.1525 | 0.0919 | 0.0952 | 0.8413 | 0.1429 |
| **Leu** | 0.1691 | 0.1461 | 0.1437 | 0.038 | 0.0709 | 0.1121 | 0.4643 | 0.4524 | 0.5476 |
| **Lys** | 0.3416 | 0.0949 | 0.1712 | 0.094 | 0.0293 | 0.1237 | 0.0238 | 0.0833 | 0.5476 |
| **Phe** | 0.0639 | 0.0797 | 0.0625 | 0.0172 | 0.0082 | 0.0182 | 0.2262 | 0.8413 | 0.2262 |
| **Pro** | 0.5237 | 0.6468 | 0.4942 | 0.1567 | 0.0716 | 0.111 | 0.4643 | 0.6905 | 0.1667 |
| **Ser*** | 0.3154 | 0.7464 | 0.3871 | 0.0573 | 0.1164 | 0.1 | 0.0119 | 0.3095 | 0.0119 |
| **Thr** | 0.9775 | 0.8244 | 0.8395 | 0.1584 | 0.1148 | 0.1903 | 0.4524 | 0.4643 | 1 |
| **Tyr** | 0.9946 | 0.4066 | 0.5844 | 0.1846 | 0.0598 | 0.1378 | 0.0238 | 0.0238 | 0.0952 |
| **Val** | 0.1998 | 0.3957 | 0.2523 | 0.0571 | 0.0999 | 0.0867 | 0.0238 | 0.2222 | 0.0833 |
| *Phenolic acids* |  |  |  |  |  |  |  |  |  |
| **caftaric acid** | 0.0009 | 0.0003 | 0.0023 | 0.0002 | 0.0002 | 0.0008 | 0.0079 | 0.0079 | 0.0079 |
| **caffeic acid*** | 0.0004 | 0.0001 | 0.0007 | 0.0001 | 0.0001 | 0.0003 | 0.0119 | 0.2222 | 0.0119 |
| **gallic acid** | 0.0167 | 0.0214 | 0.0295 | 0.0026 | 0.0036 | 0.0065 | 0.0952 | 0.0476 | 0.0833 |
| **isoferulic acid** | 0.0004 | 0.0009 | 0.0005 | 0.0001 | 0.0003 | 0.0003 | 0.0238 | 0.8413 | 0.1429 |
| *Flavonols* |  |  |  |  |  |  |  |  |  |
| **quercetin 3 glucoside** | 0.0006 | 0.0006 | 0.0006 | 0.0003 | 0.0001 | 0.0001 | 0.8413 | 0.8214 | 0.8214 |
| **quercetin 3 glucuronide*** | 0.0027 | 0.0014 | 0.0031 | 0.0021 | 0.0002 | 0.001 | 0.0476 | 0.5476 | 0.0476 |
| **kaempferol 3 glucoside** | 0 | 0.0001 | 0.0001 | 0 | 0.0001 | 0.0001 | 0.3333 | 0.3333 | 1 |
| *Flavanols* |  |  |  |  |  |  |  |  |  |
| **catechin*** | 0.2122 | 0.0351 | 0.1768 | 0.0308 | 0.0036 | 0.0676 | 0.0119 | 0.1508 | 0.0119 |
| **epicatechin*** | 0.0363 | 0.0029 | 0.0261 | 0.0048 | 0.0006 | 0.0108 | 0.0119 | 0.1508 | 0.0119 |
| **epicatechin gallate*** | 0.0443 | 0.0111 | 0.0413 | 0.005 | 0.0022 | 0.0098 | 0.0119 | 0.2222 | 0.0119 |
| **epigallocatechin*** | 0.0012 | 0.0001 | 0.0021 | 0.0002 | 0.0001 | 0.0009 | 0.0119 | 0.2222 | 0.0119 |
| **epigallocatechin gallate** | 0.005 | 0.0013 | 0.0104 | 0.0017 | 0.0004 | 0.0034 | 0.0119 | 0.0159 | 0.0119 |
| **gallocatechin*** | 0.001 | 0.0001 | 0.0019 | 0.0006 | 0.0001 | 0.0008 | 0.0119 | 0.0952 | 0.0119 |
| **B1*** | 0.1444 | 0.0195 | 0.1299 | 0.0301 | 0.0031 | 0.0328 | 0.0119 | 0.5476 | 0.0119 |
| **B2*** | 0.0399 | 0.0059 | 0.0297 | 0.0089 | 0.0008 | 0.0043 | 0.0119 | 0.0952 | 0.0119 |
| **B3*** | 0.0293 | 0.0058 | 0.0396 | 0.0082 | 0.001 | 0.0135 | 0.0119 | 0.3095 | 0.0119 |
| **B4*** | 0.0129 | 0.0021 | 0.0133 | 0.0028 | 0.0008 | 0.0031 | 0.0119 | 1 | 0.0119 |
| **B1 gallate** | 0.0948 | 0.0557 | 0.0918 | 0.0328 | 0.0189 | 0.0342 | 0.1429 | 1 | 0.1429 |
| **C1*** | 0.0485 | 0.0095 | 0.0419 | 0.0068 | 0.0041 | 0.0199 | 0.0119 | 0.1508 | 0.0119 |
| **trimer1*** | 0.0785 | 0.0234 | 0.0842 | 0.0111 | 0.0095 | 0.0168 | 0.0119 | 0.4206 | 0.0119 |
| *Stilbenes* |  |  |  |  |  |  |  |  |  |
| ***trans*-resveratrol** | 0.0567 | 0.0207 | 0.0277 | 0.0122 | 0.0071 | 0.0128 | 0.0238 | 0.0476 | 0.3095 |
| ***trans*-piceid** | 0.0326 | 0.0097 | 0.0227 | 0.0022 | 0.0023 | 0.0042 | 0.0079 | 0.0079 | 0.0079 |
| ***cis*-piceid*** | 0.0009 | 0.0019 | 0.0009 | 0.0003 | 0.0005 | 0.0003 | 0.0476 | 0.6905 | 0.0476 |
| ***trans*-piceatannol** | 0.0432 | 0.0143 | 0.0254 | 0.0057 | 0.0054 | 0.0094 | 0.0238 | 0.0238 | 0.0556 |
| ***trans*-astringin** | 0.0059 | 0.0015 | 0.0031 | 0.0007 | 0.0003 | 0.0003 | 0.0079 | 0.0079 | 0.0079 |
| ***cis*-astringin** | 0.0149 | 0.0026 | 0.0093 | 0.0035 | 0.001 | 0.0011 | 0.0079 | 0.0079 | 0.0079 |
| ***trans-*isorhapotin*** | 0.0003 | 0.0002 | 0.0004 | 0 | 0 | 0.0002 | 0.0119 | 0.5476 | 0.0119 |
| ***trans*-ε-viniferin** | 0.7377 | 0.6178 | 0.745 | 0.1351 | 0.0888 | 0.3362 | 0.631 | 0.631 | 0.8413 |
| ***trans-*ω-viniferin** | 0.0311 | 0.0271 | 0.0344 | 0.0054 | 0.0043 | 0.0097 | 0.4643 | 0.8413 | 0.4643 |
| ***cis*-ε-viniferin** | 0.0126 | 0.0137 | 0.0129 | 0.006 | 0.005 | 0.0016 | 1 | 1 | 1 |
| ***trans-*δ*-*viniferin** | 0.0131 | 0.007 | 0.0067 | 0.0044 | 0.0017 | 0.0074 | 0.0952 | 0.1508 | 0.1508 |
| **pallidol** | 0.0423 | 0.0398 | 0.0236 | 0.0108 | 0.0023 | 0.0123 | 0.6905 | 0.1429 | 0.1429 |
| **parthenocisin A*** | 0.0087 | 0.0167 | 0.0106 | 0.002 | 0.0019 | 0.0041 | 0.0238 | 0.6905 | 0.0476 |
| **ampelopsin A** | 0.0114 | 0.0073 | 0.019 | 0.0046 | 0.0037 | 0.0111 | 0.2262 | 0.4206 | 0.2262 |
| **vitisinol C*** | 0.0016 | 0.0006 | 0.0023 | 0.0007 | 0.0004 | 0.0009 | 0.0476 | 0.0952 | 0.0238 |
| **dimer diglycoside** | 0.0062 | 0.0048 | 0.0067 | 0.0016 | 0.0017 | 0.0029 | 0.4643 | 1 | 0.4643 |
| **dimer glycoside A** | 0.0032 | 0.0023 | 0.0033 | 0.0004 | 0.0005 | 0.001 | 0.0952 | 0.4206 | 0.1429 |
| **dimer glycoside B** | 0.0017 | 0.0009 | 0.0019 | 0.0013 | 0.0007 | 0.0021 | 0.631 | 1 | 0.631 |
| **dimer glycoside C** | 0.0049 | 0.0017 | 0.0036 | 0.0013 | 0.0006 | 0.003 | 0.0238 | 0.1508 | 0.1429 |
| **α-viniferin*** | 0.019 | 0.1229 | 0.0344 | 0.0069 | 0.0253 | 0.0405 | 0.0238 | 0.8413 | 0.0476 |
| **miyabenol C*** | 0.0236 | 0.0553 | 0.0273 | 0.0084 | 0.0131 | 0.017 | 0.0238 | 1 | 0.0476 |
| **hopeaphenol** | 0.2794 | 0.157 | 0.1708 | 0.1119 | 0.0357 | 0.1738 | 0.0952 | 0.2222 | 0.2222 |
| **isohopeaphenol** | 0.4564 | 0.4139 | 0.2656 | 0.1586 | 0.0473 | 0.1577 | 0.6905 | 0.2262 | 0.2262 |
| **r2-viniferin** | 0.016 | 0.0087 | 0.0255 | 0.0017 | 0.0007 | 0.006 | 0.0079 | 0.0079 | 0.0079 |
| **r-viniferin** | 0.1088 | 0.0767 | 0.158 | 0.0118 | 0.0114 | 0.0298 | 0.0119 | 0.0317 | 0.0119 |
| *Others* |  |  |  |  |  |  |  |  |  |
| **naringenin*** | 0.0004 | 0.0006 | 0.0003 | 0.0001 | 0 | 0.0001 | 0.0119 | 0.4206 | 0.0119 |
| **naringenin glucoside** | 0.0155 | 0.0039 | 0.0084 | 0.0029 | 0.0013 | 0.0051 | 0.0238 | 0.0952 | 0.0833 |
| **phloretin*** | 0.0012 | 0.0002 | 0.0013 | 0.0003 | 0 | 0.0004 | 0.0119 | 0.6905 | 0.0119 |
| **taxifolin*** | 0.0006 | 0.0016 | 0.0006 | 0.0002 | 0 | 0.0001 | 0.0119 | 0.8413 | 0.0119 |

Supplementary table 2: Metabolite concentration, protein content and percentage of water in the scion wood, graft interface and rootstock wood of hetero-grafts of Vitis vinifera cv. Merlot onto Vitis berlandieri x Vitis rupestris cv. 140 Ruggeri 33 days after grafting. Mean concentrations and standard deviation shown (n = 5). p-value of Wilcoxon-test between scion and interface tissues (S - I), scion and rootstock tissues (S - R), and interface and rootstock tissues (I - R). Stars indicates a significant difference between S – I and between I – R, but no significant difference between S - R. Significance threshold set at p-value < 0.05.

| **Variables** | **Mean** | | | **standard deviation** | | | ***p-value* wilcoxon-test** | | |
| --- | --- | --- | --- | --- | --- | --- | --- | --- | --- |
|  | Scion | Interface | Rootstock | Scion | Interface | Rootstock | S - I | S - R | I - R |
| **% of water*** | 55.6138 | 67.5459 | 54.3143 | 1.0694 | 1.1327 | 1.4634 | 0.0119 | 0.0952 | 0.0119 |
| **starch** | 83.4728 | 55.4475 | 78.0913 | 29.3678 | 10.2748 | 19.4357 | 0.2262 | 0.6905 | 0.1667 |
| **proteins** | 25.5999 | 27.395 | 24.1073 | 5.2301 | 10.2291 | 6.5552 | 1 | 1 | 1 |
| **sucrose** | 0.7523 | 0 | 0.3723 | 0.5432 | 0 | 0.5618 | 0.0761 | 0.2903 | 0.108 |
| **glucose** | 1.4475 | 2.3014 | 1.4492 | 0.3699 | 1.1361 | 0.6522 | 0.631 | 1 | 0.631 |
| **fructose*** | 1.3418 | 3.2805 | 1.3565 | 0.5137 | 0.8603 | 0.5718 | 0.0238 | 0.8413 | 0.0238 |
| *Amino acids* |  |  |  |  |  |  |  |  |  |
| **Ala*** | 0.2891 | 0.7447 | 0.3739 | 0.2009 | 0.3262 | 0.1652 | 0.0476 | 0.3095 | 0.0476 |
| **Arg** | 2.7099 | 2.5795 | 2.8621 | 1.0993 | 1.5837 | 0.1023 | 0.8413 | 0.8413 | 0.4524 |
| **Asn** | 0.7365 | 8.861 | 3.4389 | 0.1771 | 4.4509 | 1.1303 | 0.0079 | 0.0079 | 0.0079 |
| **Asp** | 1.2207 | 2.3803 | 2.2304 | 0.2394 | 1.198 | 0.4912 | 0.0476 | 0.0238 | 0.8413 |
| **GABA** | 0.4822 | 1.4261 | 0.8336 | 0.1509 | 0.6479 | 0.2412 | 0.0238 | 0.0476 | 0.0952 |
| **Gln** | 3.9046 | 19.8597 | 9.4454 | 1.2361 | 11.6447 | 2.0116 | 0.0119 | 0.0119 | 0.0159 |
| **Glu** | 1.8574 | 2.4517 | 2.774 | 0.367 | 0.9239 | 0.5187 | 0.4206 | 0.0476 | 0.4206 |
| **Gly** | 0.1233 | 0.0446 | 0.0409 | 0.268 | 0.0177 | 0.0398 | 0.2137 | 0.2137 | 0.4206 |
| **His** | 0.5545 | 0.5648 | 0.6143 | 0.3365 | 0.2628 | 0.1368 | 1 | 1 | 1 |
| **Ile** | 0.1038 | 0.4562 | 0.2229 | 0.0477 | 0.226 | 0.0594 | 0.0238 | 0.0238 | 0.0317 |
| **Leu** | 0.1094 | 0.2526 | 0.1779 | 0.0569 | 0.1119 | 0.0501 | 0.0833 | 0.0833 | 0.2222 |
| **Lys*** | 0.1358 | 0.0071 | 0.126 | 0.0753 | 0.0126 | 0.04 | 0.0318 | 0.6905 | 0.0318 |
| **Phe** | 0.0621 | 0.0977 | 0.0892 | 0.0299 | 0.0211 | 0.0203 | 0.1429 | 0.1429 | 0.5476 |
| **Pro** | 0.3218 | 1.1337 | 0.7144 | 0.0816 | 0.3159 | 0.1131 | 0.0119 | 0.0119 | 0.0317 |
| **Ser** | 0.4085 | 0.9985 | 0.4601 | 0.501 | 0.5787 | 0.1624 | 0.1429 | 0.1508 | 0.0952 |
| **Thr** | 0.6016 | 0.9078 | 0.8398 | 0.1879 | 0.4288 | 0.0715 | 0.3333 | 0.0238 | 0.8413 |
| **Tyr** | 0.4405 | 0.327 | 0.6846 | 0.0833 | 0.0757 | 0.1896 | 0.0317 | 0.0119 | 0.0119 |
| **Val*** | 0.1537 | 0.5774 | 0.3269 | 0.0915 | 0.2668 | 0.094 | 0.0238 | 0.0556 | 0.0476 |
| *Phenolic acids* |  |  |  |  |  |  |  |  |  |
| **caftaric acid** | 0.017 | 0.001 | 0.0042 | 0.0035 | 0.0003 | 0.0014 | 0.0079 | 0.0079 | 0.0079 |
| **caffeic acid*** | 0.0007 | 0.0001 | 0.001 | 0.0002 | 0.0001 | 0.0005 | 0.0119 | 0.3095 | 0.0119 |
| **gallic acid** | 0.0409 | 0.0246 | 0.0317 | 0.003 | 0.0033 | 0.0032 | 0.0119 | 0.0119 | 0.0317 |
| **isoferulic acid*** | 0.0005 | 0.0012 | 0.0004 | 0.0001 | 0.0003 | 0.0001 | 0.0119 | 0.4206 | 0.0119 |
| *Flavonols* |  |  |  |  |  |  |  |  |  |
| **quercetin 3 glucoside** | 0.0158 | 0.003 | 0.0013 | 0.0053 | 0.0006 | 0.0007 | 0.0119 | 0.0119 | 0.0159 |
| **quercetin 3 glucuronide** | 0.0167 | 0.0047 | 0.0085 | 0.0061 | 0.0012 | 0.0037 | 0.0119 | 0.0317 | 0.0119 |
| **kaempferol 3 glucoside** | 0.0053 | 0.0009 | 0.0003 | 0.0017 | 0.0002 | 0.0002 | 0.0079 | 0.0079 | 0.0079 |
| *Flavanols* |  |  |  |  |  |  |  |  |  |
| **catechin** | 0.3314 | 0.0568 | 0.2009 | 0.0363 | 0.0064 | 0.0557 | 0.0079 | 0.0079 | 0.0079 |
| **epicatechin** | 0.2139 | 0.0088 | 0.0283 | 0.0538 | 0.0017 | 0.0102 | 0.0079 | 0.0079 | 0.0079 |
| **epicatechin gallate** | 0.1143 | 0.0205 | 0.0383 | 0.0142 | 0.0022 | 0.0075 | 0.0079 | 0.0079 | 0.0079 |
| **epigallocatechin** | 0.0073 | 0.0004 | 0.0027 | 0.0039 | 0.0002 | 0.0008 | 0.0079 | 0.0079 | 0.0079 |
| **epigallocatechin gallate** | 0.0169 | 0.0016 | 0.0117 | 0.004 | 0.0003 | 0.0019 | 0.0119 | 0.0159 | 0.0119 |
| **gallocatechin** | 0.007 | 0.0004 | 0.0027 | 0.004 | 0.0002 | 0.0009 | 0.0079 | 0.0079 | 0.0079 |
| **B1** | 0.4606 | 0.0302 | 0.1417 | 0.0446 | 0.0053 | 0.029 | 0.0079 | 0.0079 | 0.0079 |
| **B2** | 0.1418 | 0.01 | 0.0345 | 0.0234 | 0.0023 | 0.0075 | 0.0079 | 0.0079 | 0.0079 |
| **B3** | 0.1291 | 0.0092 | 0.0428 | 0.0153 | 0.0017 | 0.0095 | 0.0079 | 0.0079 | 0.0079 |
| **B4** | 0.0894 | 0.0036 | 0.015 | 0.0129 | 0.0015 | 0.005 | 0.0079 | 0.0079 | 0.0079 |
| **B1 gallate** | 0.2515 | 0.0796 | 0.1024 | 0.0753 | 0.0209 | 0.0286 | 0.0119 | 0.0119 | 0.2222 |
| **C1** | 0.1558 | 0.018 | 0.0417 | 0.0329 | 0.0047 | 0.0101 | 0.0079 | 0.0079 | 0.0079 |
| **trimer1** | 0.2092 | 0.0419 | 0.0856 | 0.0256 | 0.0042 | 0.0246 | 0.0079 | 0.0079 | 0.0079 |
| *Stilbenes* |  |  |  |  |  |  |  |  |  |
| ***trans*-resveratrol** | 0.0515 | 0.0399 | 0.0357 | 0.0347 | 0.013 | 0.0103 | 0.8413 | 0.8413 | 0.8413 |
| ***trans*-piceid** | 0.0517 | 0.0158 | 0.0323 | 0.0105 | 0.0036 | 0.0091 | 0.0119 | 0.0317 | 0.0119 |
| ***cis*-piceid** | 0.0011 | 0.002 | 0.0011 | 0.0001 | 0.0005 | 0.0008 | 0.0238 | 0.1508 | 0.1429 |
| ***trans*-piceatannol** | 0.0225 | 0.0196 | 0.0254 | 0.0094 | 0.0041 | 0.0063 | 0.8413 | 0.631 | 0.631 |
| ***trans*-astringin** | 0.003 | 0.0021 | 0.0041 | 0.0006 | 0.0003 | 0.0008 | 0.0238 | 0.0317 | 0.0238 |
| ***cis*-astringin** | 0.2646 | 0.0134 | 0.0124 | 0.0279 | 0.0024 | 0.0034 | 0.0119 | 0.0119 | 0.8413 |
| ***trans-isorhapotin**** | 0.0007 | 0.0003 | 0.0003 | 0.0002 | 0.0001 | 0.0001 | 0.0119 | 0.0119 | 0.8413 |
| ***trans*-ε-viniferin** | 0.7323 | 0.7409 | 0.6842 | 0.1082 | 0.0647 | 0.0349 | 1 | 0.8214 | 0.2857 |
| ***trans-*ω-viniferin** | 0.0243 | 0.0365 | 0.0394 | 0.0064 | 0.0035 | 0.0058 | 0.0238 | 0.0238 | 0.4206 |
| ***cis*-ε-viniferin** | 0.0113 | 0.0216 | 0.0154 | 0.0031 | 0.0088 | 0.0096 | 0.1667 | 0.8413 | 0.4643 |
| ***trans-*δ*-*viniferin** | 0.0176 | 0.0095 | 0.0037 | 0.0043 | 0.0018 | 0.0018 | 0.0159 | 0.0119 | 0.0119 |
| **pallidol** | 0.0287 | 0.0418 | 0.0214 | 0.0132 | 0.0098 | 0.0045 | 0.2262 | 0.8413 | 0.0238 |
| **parthenocisin A*** | 0.0128 | 0.0225 | 0.009 | 0.0051 | 0.0031 | 0.0009 | 0.0119 | 0.5476 | 0.0119 |
| **ampelopsin A** | 0.0387 | 0.0124 | 0.012 | 0.0151 | 0.0031 | 0.0054 | 0.0119 | 0.0119 | 0.8413 |
| **vitisinol C** | 0.0034 | 0.0013 | 0.0024 | 0.0002 | 0.0003 | 0.0004 | 0.0079 | 0.0079 | 0.0079 |
| **dimer diglycoside** | 0.005 | 0.0054 | 0.0086 | 0.0011 | 0.002 | 0.0041 | 1 | 0.4643 | 0.4643 |
| **dimer glycoside A** | 0.0025 | 0.0027 | 0.0042 | 0.0004 | 0.0005 | 0.0013 | 0.8413 | 0.1667 | 0.2262 |
| **dimer glycoside B** | 0.0063 | 0.0017 | 0.0011 | 0.0009 | 0.0002 | 0.0003 | 0.0119 | 0.0119 | 0.0317 |
| **dimer glycoside C** | 0.0051 | 0.0031 | 0.0027 | 0.0013 | 0.0005 | 0.0007 | 0.0119 | 0.0119 | 0.5476 |
| **α-viniferin** | 0.0717 | 0.1728 | 0.0207 | 0.0139 | 0.0276 | 0.0141 | 0.0079 | 0.0079 | 0.0079 |
| **miyabenol C** | 0.0522 | 0.1121 | 0.0246 | 0.0142 | 0.0231 | 0.0067 | 0.0119 | 0.0159 | 0.0119 |
| **hopeaphenol** | 0.2401 | 0.1543 | 0.0919 | 0.0442 | 0.0256 | 0.0331 | 0.0119 | 0.0119 | 0.0556 |
| **isohopeaphenol*** | 0.1641 | 0.3219 | 0.1313 | 0.0581 | 0.0912 | 0.0602 | 0.0119 | 0.4206 | 0.0119 |
| **r2-viniferin** | 0.0087 | 0.0072 | 0.0291 | 0.0031 | 0.0009 | 0.0047 | 0.4206 | 0.0119 | 0.0119 |
| **r-viniferin** | 0.0652 | 0.0629 | 0.1668 | 0.0179 | 0.005 | 0.0216 | 0.8413 | 0.0119 | 0.0119 |
| *Others* |  |  |  |  |  |  |  |  |  |
| **naringenin** | 0.0012 | 0.0034 | 0.0006 | 0.0002 | 0.0005 | 0.0001 | 0.0079 | 0.0079 | 0.0079 |
| **naringenin glucoside** | 1.9231 | 0.0673 | 0.0127 | 0.1624 | 0.0148 | 0.0042 | 0.0079 | 0.0079 | 0.0079 |
| **phloretin*** | 0.0011 | 0.0003 | 0.0015 | 0.0002 | 0.0001 | 0.0003 | 0.0119 | 0.0556 | 0.0119 |
| **taxifolin** | 0.0013 | 0.0029 | 0.0006 | 0.0002 | 0.0002 | 0.0002 | 0.0079 | 0.0079 | 0.0079 |

Supplementary table 3: Metabolite concentration, protein content and percentage of water in the scion wood, graft interface and rootstock wood of homo-grafts of Vitis vinifera cv. Merlot 33 days after grafting. Mean concentrations and standard deviation shown (n = 5). p-value of Wilcoxon-test between scion and interface tissues (S – I), scion and rootstock tissues (S – R), and interface and rootstock tissues (I -R). Stars indicates a significant difference between S – I and between I – R, but no significant difference between S - R. Significance threshold set at p-value < 0.05.

| **Variables** | **Mean** | | | **standard deviation** | | | ***p-value* wilcoxon-test** | | |
| --- | --- | --- | --- | --- | --- | --- | --- | --- | --- |
|  | Scion | Interface | Rootstock | Scion | Interface | Rootstock | S - I | S - R | I - R |
| **% of water*** | 54.2281 | 67.6134 | 55.318 | 1.1472 | 1.123 | 0.9129 | 0.0119 | 0.0952 | 0.0119 |
| **starch*** | 101.7716 | 58.4426 | 107.0275 | 17.0533 | 11.3956 | 9.117 | 0.0119 | 0.4206 | 0.0119 |
| **proteins*** | 23.6707 | 37.5317 | 23.1552 | 3.4905 | 4.0134 | 7.3124 | 0.0238 | 0.4206 | 0.0238 |
| **sucrose** | 1.6998 | 1.1291 | 1.292 | 0.3757 | 0.6905 | 0.4467 | 0.4524 | 0.4643 | 0.6905 |
| **glucose** | 1.4413 | 2.0092 | 1.1153 | 0.624 | 0.3133 | 0.2534 | 0.1429 | 0.3095 | 0.0476 |
| **fructose*** | 1.4528 | 3.458 | 1.4772 | 0.2488 | 0.3685 | 0.545 | 0.0119 | 1 | 0.0119 |
| *Amino acids* |  |  |  |  |  |  |  |  |  |
| **Ala*** | 0.3725 | 0.8376 | 0.3478 | 0.1087 | 0.1161 | 0.1114 | 0.0119 | 0.8413 | 0.0119 |
| **Arg** | 5.9568 | 6.3628 | 5.0259 | 1.7586 | 1.6477 | 0.5893 | 0.8413 | 0.4643 | 0.4643 |
| **Asn*** | 0.8014 | 11.2668 | 1.5468 | 0.3619 | 1.6906 | 0.9613 | 0.0119 | 0.0952 | 0.0119 |
| **Asp*** | 2.2425 | 3.107 | 2.1058 | 0.4406 | 0.3052 | 0.3392 | 0.0238 | 0.6905 | 0.0238 |
| **GABA*** | 0.7507 | 1.7977 | 0.8097 | 0.116 | 0.2884 | 0.2113 | 0.0119 | 0.8413 | 0.0119 |
| **Gln*** | 9.1453 | 29.4157 | 9.0462 | 1.698 | 4.638 | 1.9372 | 0.0119 | 1 | 0.0119 |
| **Glu** | 2.9229 | 3.7634 | 3.1814 | 1.5924 | 0.4872 | 0.6577 | 0.6905 | 0.6905 | 0.6667 |
| **Gly** | 0.0023 | 0.0272 | 0.0023 | 0.0052 | 0.0289 | 0.0032 | 0.1004 | 0.7972 | 0.1004 |
| **His** | 0.5131 | 0.2952 | 0.3478 | 0.3379 | 0.2938 | 0.1348 | 0.631 | 0.631 | 0.8413 |
| **Ile*** | 0.2326 | 1.0269 | 0.2465 | 0.1087 | 0.3428 | 0.0851 | 0.0119 | 0.8413 | 0.0119 |
| **Leu** | 0.2062 | 0.3622 | 0.2467 | 0.0811 | 0.092 | 0.0833 | 0.0952 | 0.5476 | 0.1429 |
| **Lys** | 0.3023 | 0.3447 | 0.2315 | 0.0861 | 0.3704 | 0.0545 | 0.631 | 0.631 | 0.8413 |
| **Phe*** | 0.0822 | 0.1145 | 0.0839 | 0.0159 | 0.0037 | 0.0234 | 0.0238 | 1 | 0.0476 |
| **Pro*** | 0.617 | 1.1553 | 0.6535 | 0.1826 | 0.3315 | 0.1846 | 0.0476 | 1 | 0.0476 |
| **Ser*** | 0.3543 | 1.377 | 0.4273 | 0.089 | 0.1487 | 0.0976 | 0.0119 | 0.4206 | 0.0119 |
| **Thr** | 1.2608 | 1.5328 | 1.0685 | 0.6273 | 0.2492 | 0.1824 | 0.8214 | 1 | 0.0476 |
| **Tyr*** | 0.6573 | 0.3045 | 0.5803 | 0.1105 | 0.0821 | 0.1009 | 0.0119 | 0.4206 | 0.0119 |
| **Val*** | 0.2815 | 0.7612 | 0.3017 | 0.0842 | 0.2026 | 0.0874 | 0.0119 | 1 | 0.0119 |
| *Phenolic acids* |  |  |  |  |  |  |  |  |  |
| **caftaric acid** | 0.0126 | 0.0009 | 0.0071 | 0.0013 | 0.0003 | 0.0015 | 0.0079 | 0.0079 | 0.0079 |
| **caffeic acid** | 0.0005 | 0.0001 | 0.0003 | 0.0001 | 0 | 0.0001 | 0.0079 | 0.0079 | 0.0079 |
| **gallic acid** | 0.0272 | 0.0134 | 0.0209 | 0.0016 | 0.0017 | 0.0034 | 0.0079 | 0.0079 | 0.0079 |
| **isoferulic acid** | 0.0003 | 0.0004 | 0.0002 | 0.0001 | 0.0002 | 0.0001 | 0.8413 | 0.631 | 0.631 |
| *Flavonols* |  |  |  |  |  |  |  |  |  |
| **quercetin 3 glucoside** | 0.0116 | 0.0069 | 0.0076 | 0.0038 | 0.0016 | 0.0018 | 0.1429 | 0.1429 | 0.8413 |
| **quercetin 3 glucuronide** | 0.0129 | 0.0059 | 0.008 | 0.005 | 0.0009 | 0.0019 | 0.0238 | 0.1429 | 0.1508 |
| **kaempferol 3 glucoside** | 0.0048 | 0.0023 | 0.0032 | 0.0014 | 0.0003 | 0.0008 | 0.0238 | 0.0952 | 0.0833 |
| *Flavanols* |  |  |  |  |  |  |  |  |  |
| **catechin*** | 0.2517 | 0.0506 | 0.2059 | 0.0263 | 0.0102 | 0.0396 | 0.0119 | 0.0952 | 0.0119 |
| **epicatechin** | 0.193 | 0.0107 | 0.1178 | 0.021 | 0.0026 | 0.0289 | 0.0079 | 0.0079 | 0.0079 |
| **epicatechin gallate** | 0.1099 | 0.0321 | 0.0902 | 0.0046 | 0.0044 | 0.0161 | 0.0119 | 0.0317 | 0.0119 |
| **epigallocatechin** | 0.005 | 0.0004 | 0.0024 | 0.0017 | 0.0001 | 0.0009 | 0.0119 | 0.0159 | 0.0119 |
| **epigallocatechin gallate** | 0.0187 | 0.0023 | 0.0143 | 0.0022 | 0.0004 | 0.002 | 0.0119 | 0.0317 | 0.0119 |
| **gallocatechin** | 0.004 | 0.0004 | 0.0017 | 0.0008 | 0.0003 | 0.0003 | 0.0079 | 0.0079 | 0.0079 |
| **B1*** | 0.3215 | 0.0609 | 0.2602 | 0.0166 | 0.0113 | 0.0413 | 0.0119 | 0.0556 | 0.0119 |
| **B2*** | 0.0872 | 0.0182 | 0.0745 | 0.0119 | 0.0037 | 0.0118 | 0.0119 | 0.1508 | 0.0119 |
| **B3** | 0.09 | 0.0143 | 0.072 | 0.0069 | 0.0038 | 0.0098 | 0.0119 | 0.0317 | 0.0119 |
| **B4*** | 0.0623 | 0.0053 | 0.0466 | 0.0062 | 0.0016 | 0.0136 | 0.0119 | 0.1508 | 0.0119 |
| **B1 gallate** | 0.2194 | 0.1094 | 0.1911 | 0.0149 | 0.0152 | 0.0182 | 0.0119 | 0.0317 | 0.0119 |
| **C1*** | 0.1235 | 0.0365 | 0.1086 | 0.035 | 0.0099 | 0.0322 | 0.0119 | 0.2222 | 0.0119 |
| **trimer1*** | 0.1637 | 0.0615 | 0.1483 | 0.0112 | 0.0106 | 0.0182 | 0.0119 | 0.2222 | 0.0119 |
| *Stilbenes* |  |  |  |  |  |  |  |  |  |
| ***trans*-resveratrol** | 0.0321 | 0.028 | 0.0196 | 0.0082 | 0.0037 | 0.0025 | 0.6905 | 0.0119 | 0.0119 |
| ***trans*-piceid** | 0.0438 | 0.0116 | 0.0273 | 0.0028 | 0.0027 | 0.0024 | 0.0079 | 0.0079 | 0.0079 |
| ***cis*-piceid*** | 0.0009 | 0.002 | 0.0008 | 0.0002 | 0.0005 | 0.0002 | 0.0119 | 0.4206 | 0.0119 |
| ***trans*-piceatannol** | 0.0167 | 0.0196 | 0.0128 | 0.0039 | 0.0036 | 0.0017 | 0.3095 | 0.2262 | 0.0476 |
| ***trans*-astringin** | 0.0031 | 0.0018 | 0.0019 | 0.0005 | 0.0003 | 0.0003 | 0.0119 | 0.0119 | 0.5476 |
| ***cis*-astringin** | 0.217 | 0.0303 | 0.1207 | 0.0178 | 0.0054 | 0.0202 | 0.0079 | 0.0079 | 0.0079 |
| ***trans-isorhapotin**** | 0.0008 | 0.0007 | 0.0007 | 0.0001 | 0.0002 | 0.0003 | 1 | 1 | 1 |
| ***trans*-ε-viniferin*** | 0.5958 | 0.7421 | 0.5233 | 0.0902 | 0.0317 | 0.0513 | 0.0119 | 0.2222 | 0.0119 |
| ***trans-*ω-viniferin*** | 0.0181 | 0.0418 | 0.0151 | 0.0059 | 0.0041 | 0.0025 | 0.0119 | 0.4206 | 0.0119 |
| ***cis*-ε-viniferin** | 0.0144 | 0.0173 | 0.0122 | 0.003 | 0.0055 | 0.0041 | 0.4206 | 0.4206 | 0.4206 |
| ***trans-*δ*-*viniferin** | 0.018 | 0.0123 | 0.0143 | 0.0026 | 0.0013 | 0.0018 | 0.0238 | 0.0238 | 0.0952 |
| **pallidol*** | 0.024 | 0.0437 | 0.0211 | 0.0029 | 0.0017 | 0.0047 | 0.0119 | 0.3095 | 0.0119 |
| **parthenocisin A** | 0.0102 | 0.0291 | 0.0073 | 0.003 | 0.0024 | 0.0006 | 0.0119 | 0.0159 | 0.0119 |
| **ampelopsin A** | 0.0274 | 0.016 | 0.0232 | 0.0037 | 0.0028 | 0.0052 | 0.0238 | 0.3095 | 0.0833 |
| **vitisinol C*** | 0.0043 | 0.0026 | 0.0045 | 0.0012 | 0.0005 | 0.0004 | 0.0119 | 0.1508 | 0.0119 |
| **dimer diglycoside** | 0.0039 | 0.0042 | 0.0034 | 0.0007 | 0.0002 | 0.0004 | 0.3095 | 0.3095 | 0.0476 |
| **dimer glycoside A** | 0.0022 | 0.0025 | 0.0021 | 0.0003 | 0.0002 | 0.0003 | 0.3333 | 0.4206 | 0.1667 |
| **dimer glycoside B** | 0.0048 | 0.0034 | 0.0052 | 0.0013 | 0.0007 | 0.0009 | 0.1429 | 0.6905 | 0.0476 |
| **dimer glycoside C** | 0.0042 | 0.0038 | 0.0035 | 0.0006 | 0.0004 | 0.0005 | 0.5476 | 0.4524 | 0.5476 |
| **α-viniferin*** | 0.0819 | 0.2784 | 0.099 | 0.0263 | 0.0537 | 0.0296 | 0.0119 | 0.3095 | 0.0119 |
| **miyabenol C*** | 0.0655 | 0.1954 | 0.0388 | 0.0309 | 0.0331 | 0.0113 | 0.0119 | 0.2222 | 0.0119 |
| **hopeaphenol** | 0.2368 | 0.1649 | 0.153 | 0.0453 | 0.0223 | 0.0257 | 0.0119 | 0.0119 | 0.5476 |
| **isohopeaphenol** | 0.1938 | 0.2311 | 0.0691 | 0.092 | 0.0385 | 0.011 | 0.4206 | 0.0119 | 0.0119 |
| **r2-viniferin** | 0.0062 | 0.0055 | 0.0079 | 0.0014 | 0.0017 | 0.0036 | 0.6905 | 0.6905 | 0.6905 |
| **r-viniferin** | 0.0488 | 0.0474 | 0.0571 | 0.0062 | 0.0167 | 0.025 | 0.631 | 1 | 0.631 |
| *Others* |  |  |  |  |  |  |  |  |  |
| **naringenin** | 0.001 | 0.0016 | 0.0017 | 0.0002 | 0.0017 | 0.0018 | 1 | 1 | 1 |
| **naringenin glucoside** | 1.572 | 0.6541 | 1.2036 | 0.0868 | 0.3079 | 0.3848 | 0.0119 | 0.0119 | 0.0952 |
| **phloretin*** | 0.0012 | 0.0003 | 0.0009 | 0.0001 | 0.0002 | 0.0004 | 0.0238 | 0.5476 | 0.0238 |
| **taxifolin** | 0.0015 | 0.0019 | 0.0016 | 0.0004 | 0.0007 | 0.0003 | 0.8214 | 0.8214 | 0.8413 |

Supplementary table 4: Metabolite concentration, protein content and percentage of water in the scion wood, graft interface and rootstock wood of hetero-grafts of Vitis vinifera cv. Merlot onto Vitis berlandieri x Vitis riparia cv. Rességuier Sélection Birolleau 1, 33 days after grafting. Mean concentrations and standard deviation shown (n = 5). p-value of Wilcoxon-test between scion and interface tissues (S – I), scion and rootstock tissues (S – R), and interface and rootstock tissues (I -R). Stars indicates a significant difference between S – I and between I – R, but no significant difference between S - R. Significance threshold set at p-value < 0.05.

| **Variables** | **Mean** | | | **standard deviation** | | | ***p-value* wilcoxon-test** | | |
| --- | --- | --- | --- | --- | --- | --- | --- | --- | --- |
|  | Scion | Interface | Rootstock | Scion | Interface | Rootstock | S - I | S - R | I - R |
| **% of water** | 56.4861 | 73.1269 | 59.1998 | 2.0264 | 2.8215 | 1.2749 | 0.0238 | 0.0317 | 0.0238 |
| **starch** | 75.4806 | 65.8828 | 55.2645 | 20.5578 | 12.5196 | 19.8256 | 0.3095 | 0.3095 | 0.3095 |
| **proteins*** | 21.5061 | 49.6096 | 22.4267 | 4.6241 | 9.0641 | 5.6908 | 0.0238 | 0.8413 | 0.0238 |
| **sucrose** | 1.2542 | 1.9257 | 0.9345 | 0.7619 | 0.705 | 0.6379 | 0.4286 | 0.6905 | 0.1905 |
| **glucose** | 1.4697 | 1.6581 | 1.366 | 0.4402 | 0.5634 | 0.7691 | 0.7302 | 0.7302 | 0.7302 |
| **fructose*** | 1.6143 | 3.3154 | 0.882 | 0.6144 | 0.5543 | 0.3846 | 0.0238 | 0.0556 | 0.0238 |
| *Amino acids* |  |  |  |  |  |  |  |  |  |
| **Ala*** | 0.6669 | 1.6436 | 0.5729 | 0.2336 | 0.2911 | 0.136 | 0.0238 | 0.8413 | 0.0238 |
| **Arg** | 4.3148 | 5.2182 | 7.4575 | 1.1249 | 2.067 | 3.2648 | 0.5556 | 0.4524 | 0.5556 |
| **Asn** | 0.6484 | 8.3795 | 5.8861 | 0.2175 | 2.1201 | 1.3339 | 0.0238 | 0.0238 | 0.1111 |
| **Asp** | 2.1864 | 3.9552 | 3.2037 | 0.2531 | 1.3629 | 0.9799 | 0.1429 | 0.1429 | 0.4127 |
| **GABA*** | 1.1771 | 2.7469 | 1.5155 | 0.2115 | 0.6356 | 0.572 | 0.0476 | 0.4206 | 0.0476 |
| **Gln** | 6.4391 | 26.8101 | 15.0443 | 1.2075 | 7.2059 | 5.3623 | 0.0238 | 0.0238 | 0.0635 |
| **Glu** | 3.5147 | 4.5453 | 4.0628 | 0.5115 | 1.794 | 1.2211 | 0.7302 | 0.7302 | 0.7302 |
| **Gly** | 0.1271 | 0.2306 | 0.0843 | 0.1961 | 0.2662 | 0.0523 | 0.2857 | 0.4206 | 0.2857 |
| **His** | 0.8123 | 0.5272 | 1.5591 | 0.2111 | 0.3771 | 0.4938 | 0.4127 | 0.0476 | 0.0476 |
| **Ile** | 0.2124 | 1.1974 | 0.5451 | 0.03 | 0.2336 | 0.1262 | 0.0159 | 0.0159 | 0.0159 |
| **Leu** | 0.2209 | 0.6978 | 0.6766 | 0.0463 | 0.146 | 0.1321 | 0.0238 | 0.0238 | 0.9048 |
| **Lys** | 0.3973 | 0.1354 | 0.7753 | 0.0933 | 0.1896 | 0.2907 | 0.1111 | 0.0833 | 0.0833 |
| **Phe** | 0.1172 | 0.2667 | 0.209 | 0.0218 | 0.0824 | 0.0601 | 0.0476 | 0.0476 | 0.4127 |
| **Pro** | 0.5251 | 1.102 | 0.9327 | 0.1446 | 0.421 | 0.2179 | 0.0952 | 0.0952 | 0.7302 |
| **Ser*** | 0.5464 | 1.6394 | 0.7151 | 0.3226 | 0.5384 | 0.1884 | 0.0476 | 0.1508 | 0.0476 |
| **Thr** | 1.2527 | 1.5395 | 2.0106 | 0.3407 | 0.3295 | 0.5348 | 0.2857 | 0.0476 | 0.2857 |
| **Tyr*** | 0.798 | 0.4023 | 1.0323 | 0.1463 | 0.131 | 0.2775 | 0.0238 | 0.1508 | 0.0238 |
| **Val** | 0.2808 | 1.2742 | 0.7353 | 0.0553 | 0.2771 | 0.1439 | 0.0238 | 0.0238 | 0.0317 |
| *Phenolic acids* |  |  |  |  |  |  |  |  |  |
| **caftaric acid** | 0.0143 | 0.0007 | 0.0034 | 0.0066 | 0.0001 | 0.001 | 0.0159 | 0.0159 | 0.0159 |
| **caffeic acid** | 0.0003 | 0.0001 | 0.0002 | 0.0001 | 0 | 0.0001 | 0.0584 | 0.0833 | 0.5386 |
| **gallic acid** | 0.0427 | 0.0205 | 0.0214 | 0.0082 | 0.0042 | 0.0021 | 0.0238 | 0.0238 | 0.7302 |
| **isoferulic acid** | 0.0004 | 0.0009 | 0.0005 | 0.0001 | 0.0002 | 0.0001 | 0.0238 | 0.0238 | 0.0317 |
| *Flavonols* |  |  |  |  |  |  |  |  |  |
| **quercetin 3 glucoside** | 0.0123 | 0.0025 | 0.0004 | 0.005 | 0.0005 | 0.0001 | 0.0159 | 0.0159 | 0.0159 |
| **quercetin 3 glucuronide** | 0.012 | 0.0021 | 0.0003 | 0.004 | 0.0005 | 0.0002 | 0.0159 | 0.0159 | 0.0159 |
| **kaempferol 3 glucoside** | 0.0043 | 0.0008 | 0.0001 | 0.0016 | 0.0001 | 0 | 0.0159 | 0.0159 | 0.0159 |
| *Flavanols* |  |  |  |  |  |  |  |  |  |
| **catechin** | 0.3203 | 0.0378 | 0.1545 | 0.0674 | 0.0105 | 0.0281 | 0.0159 | 0.0159 | 0.0159 |
| **epicatechin** | 0.2178 | 0.0085 | 0.0159 | 0.0397 | 0.0027 | 0.0044 | 0.0238 | 0.0238 | 0.0317 |
| **epicatechin gallate** | 0.1183 | 0.0128 | 0.0239 | 0.0113 | 0.0039 | 0.0021 | 0.0159 | 0.0159 | 0.0159 |
| **epigallocatechin** | 0.0062 | 0.0003 | 0.0008 | 0.0016 | 0.0002 | 0.0002 | 0.0238 | 0.0238 | 0.0317 |
| **epigallocatechin gallate** | 0.0166 | 0.0013 | 0.0055 | 0.0026 | 0.0008 | 0.0011 | 0.0159 | 0.0159 | 0.0159 |
| **gallocatechin** | 0.0067 | 0.0004 | 0.0008 | 0.0021 | 0.0002 | 0.0003 | 0.0238 | 0.0238 | 0.0635 |
| **B1** | 0.4567 | 0.0326 | 0.1805 | 0.0959 | 0.0092 | 0.035 | 0.0159 | 0.0159 | 0.0159 |
| **B2** | 0.1295 | 0.0076 | 0.0204 | 0.0262 | 0.003 | 0.0037 | 0.0159 | 0.0159 | 0.0159 |
| **B3** | 0.1291 | 0.0077 | 0.0307 | 0.0367 | 0.0026 | 0.0054 | 0.0159 | 0.0159 | 0.0159 |
| **B4** | 0.0862 | 0.0042 | 0.0129 | 0.0202 | 0.0012 | 0.0054 | 0.0159 | 0.0159 | 0.0159 |
| **B1 gallate** | 0.2248 | 0.0366 | 0.0394 | 0.0372 | 0.0064 | 0.0042 | 0.0238 | 0.0238 | 0.5556 |
| **C1** | 0.136 | 0.021 | 0.0305 | 0.0243 | 0.0108 | 0.0055 | 0.0238 | 0.0238 | 0.2857 |
| **trimer1** | 0.2093 | 0.0417 | 0.1351 | 0.0272 | 0.0087 | 0.028 | 0.0159 | 0.0159 | 0.0159 |
| *Stilbenes* |  |  |  |  |  |  |  |  |  |
| ***trans*-resveratrol** | 0.0281 | 0.0161 | 0.0205 | 0.0025 | 0.0041 | 0.003 | 0.0238 | 0.0238 | 0.1111 |
| ***trans*-piceid** | 0.0485 | 0.0074 | 0.0195 | 0.0025 | 0.0021 | 0.0034 | 0.0159 | 0.0159 | 0.0159 |
| ***cis*-piceid** | 0.0011 | 0.0014 | 0.0005 | 0.0002 | 0.0006 | 0.0002 | 0.9048 | 0.0476 | 0.0476 |
| ***trans*-piceatannol*** | 0.0162 | 0.0091 | 0.0171 | 0.0041 | 0.0034 | 0.0026 | 0.0476 | 0.6905 | 0.0476 |
| ***trans*-astringin*** | 0.0041 | 0.0013 | 0.0031 | 0.0009 | 0.0003 | 0.0005 | 0.0238 | 0.0952 | 0.0238 |
| ***cis*-astringin** | 0.2232 | 0.0085 | 0.0113 | 0.0216 | 0.0025 | 0.0022 | 0.0238 | 0.0238 | 0.1905 |
| ***trans-isorhapotin**** | 0.0006 | 0.0003 | 0.0006 | 0.0001 | 0.0001 | 0.0001 | 0.0476 | 0.2222 | 0.0476 |
| ***trans*-ε-viniferin** | 0.584 | 0.6266 | 0.9996 | 0.0974 | 0.1398 | 0.068 | 0.7302 | 0.0238 | 0.0238 |
| ***trans-*ω-viniferin** | 0.016 | 0.0247 | 0.0413 | 0.0025 | 0.0064 | 0.0056 | 0.0635 | 0.0238 | 0.0238 |
| ***cis*-ε-viniferin** | 0.0111 | 0.0159 | 0.0233 | 0.006 | 0.0066 | 0.0194 | 0.8333 | 0.1667 | 0.9048 |
| ***trans-*δ*-*viniferin*** | 0.0195 | 0.0122 | 0.0179 | 0.003 | 0.0027 | 0.0022 | 0.0238 | 0.3095 | 0.0238 |
| **pallidol** | 0.0221 | 0.0412 | 0.0412 | 0.0042 | 0.0088 | 0.008 | 0.0238 | 0.0238 | 0.9048 |
| **parthenocisin A*** | 0.0085 | 0.0165 | 0.0095 | 0.0023 | 0.0037 | 0.0032 | 0.0476 | 0.6905 | 0.0476 |
| **ampelopsin A** | 0.0392 | 0.0115 | 0.0215 | 0.0105 | 0.0038 | 0.0145 | 0.0476 | 0.0833 | 0.1905 |
| **vitisinol C** | 0.0031 | 0.0009 | 0.0019 | 0.0005 | 0.0006 | 0.0005 | 0.0238 | 0.0238 | 0.0635 |
| **dimer diglycoside** | 0.004 | 0.0026 | 0.0033 | 0.0003 | 0.0003 | 0.0002 | 0.0159 | 0.0159 | 0.0159 |
| **dimer glycoside A*** | 0.0023 | 0.0015 | 0.0022 | 0.0003 | 0.0003 | 0.0002 | 0.0238 | 0.4206 | 0.0238 |
| **dimer glycoside B** | 0.0054 | 0.0013 | 0.0015 | 0.0019 | 0.0004 | 0.0004 | 0.0238 | 0.0238 | 0.5556 |
| **dimer glycoside C** | 0.004 | 0.0019 | 0.005 | 0.0004 | 0.0005 | 0.0003 | 0.0159 | 0.0159 | 0.0159 |
| **α-viniferin*** | 0.0727 | 0.1774 | 0.0875 | 0.0076 | 0.0278 | 0.0436 | 0.0476 | 1 | 0.0476 |
| **miyabenol C*** | 0.034 | 0.0584 | 0.026 | 0.002 | 0.008 | 0.0057 | 0.0238 | 0.0556 | 0.0238 |
| **hopeaphenol** | 0.2137 | 0.1221 | 0.1117 | 0.0231 | 0.0296 | 0.0253 | 0.0238 | 0.0238 | 0.4127 |
| **isohopeaphenol** | 0.1229 | 0.2212 | 0.2922 | 0.0204 | 0.049 | 0.0543 | 0.0238 | 0.0238 | 0.1111 |
| **r2-viniferin** | 0.0089 | 0.0069 | 0.0285 | 0.0028 | 0.0015 | 0.0085 | 0.1905 | 0.0238 | 0.0238 |
| **r-viniferin** | 0.055 | 0.0682 | 0.2147 | 0.0379 | 0.0093 | 0.0659 | 0.1905 | 0.0238 | 0.0238 |
| *Others* |  |  |  |  |  |  |  |  |  |
| **naringenin** | 0.0009 | 0.003 | 0.0005 | 0.0002 | 0.0004 | 0.0001 | 0.0159 | 0.0159 | 0.0159 |
| **naringenin glucoside** | 1.868 | 0.0656 | 0.014 | 0.1181 | 0.0162 | 0.003 | 0.0159 | 0.0159 | 0.0159 |
| **phloretin*** | 0.0009 | 0.0001 | 0.0008 | 0.0003 | 0.0001 | 0.0003 | 0.0238 | 0.8413 | 0.0238 |
| **taxifolin** | 0.0009 | 0.0021 | 0.0006 | 0.0002 | 0.0001 | 0.0001 | 0.0238 | 0.0317 | 0.0238 |

Supplementary table 5: Metabolite concentration, protein content and percentage of water in the scion wood, graft interface and rootstock wood of hetero-grafts of Vitis vinifera cv. Merlot onto Vitis berlandieri x Vitis riparia cv. Sélection Oppenheim 4, 33 days after grafting. Mean concentrations and standard deviation shown (n = 5). p-value of Wilcoxon-test between scion and interface tissues (S – I), scion and rootstock tissues (S – R), and interface and rootstock tissues (I -R). Stars indicates a significant difference between S – I and between I – R, but no significant difference between S - R. Significance threshold set at p-value < 0.05.

| **Variables** | **Mean** | | | **standard deviation** | | | ***p-value* wilcoxon-test** | | |
| --- | --- | --- | --- | --- | --- | --- | --- | --- | --- |
|  | Scion | Interface | Rootstock | Scion | Interface | Rootstock | S - I | S - R | I - R |
| **% of water** | 54.8225 | 64.524 | 55.1969 | 1.0146 | 5.8587 | 1.7503 | 0.0833 | 1 | 0.0833 |
| **starch** | 93.8475 | 68.6738 | 67.0799 | 12.0474 | 12.4885 | 5.96 | 0.0238 | 0.0238 | 0.8413 |
| **proteins** | 23.8731 | 36.9636 | 31.9617 | 2.9647 | 9.3897 | 7.2437 | 0.0476 | 0.0833 | 0.4206 |
| **sucrose** | 0.9587 | 2.585 | 0.8538 | 0.5868 | 1.6033 | 0.5859 | 0.1429 | 0.8413 | 0.1429 |
| **glucose*** | 1.4634 | 2.9255 | 1.5168 | 0.2548 | 0.5722 | 0.2485 | 0.0119 | 0.5476 | 0.0119 |
| **fructose*** | 1.5732 | 4.3489 | 1.2811 | 0.5245 | 0.8936 | 0.1926 | 0.0119 | 0.2222 | 0.0119 |
| *Amino acids* |  |  |  |  |  |  |  |  |  |
| **Ala*** | 0.4785 | 1.1851 | 0.3915 | 0.1246 | 0.3377 | 0.0607 | 0.0119 | 0.2222 | 0.0119 |
| **Arg** | 3.1126 | 7.0513 | 4.9891 | 0.6112 | 1.682 | 0.6754 | 0.0119 | 0.0119 | 0.0952 |
| **Asn** | 0.9089 | 18.7177 | 7.5646 | 0.2123 | 3.5229 | 0.9279 | 0.0079 | 0.0079 | 0.0079 |
| **Asp** | 1.6653 | 3.4463 | 2.3146 | 0.2246 | 0.4383 | 0.2561 | 0.0079 | 0.0079 | 0.0079 |
| **GABA*** | 0.7287 | 2.6825 | 0.999 | 0.1969 | 0.4486 | 0.1272 | 0.0119 | 0.0556 | 0.0119 |
| **Gln** | 4.2132 | 28.159 | 10.6304 | 1.3656 | 1.8873 | 0.7149 | 0.0079 | 0.0079 | 0.0079 |
| **Glu** | 2.6793 | 3.5581 | 2.9917 | 0.5374 | 0.4714 | 0.3919 | 0.1429 | 0.3095 | 0.1429 |
| **Gly** | 0.1286 | 0.0513 | 0.0219 | 0.1636 | 0.0387 | 0.03 | 0.8413 | 0.5964 | 0.4274 |
| **His** | 0.4955 | 0.5811 | 0.4708 | 0.1845 | 0.3575 | 0.3059 | 0.8413 | 0.8413 | 0.8413 |
| **Ile** | 0.1626 | 1.7224 | 0.5388 | 0.036 | 0.2098 | 0.053 | 0.0079 | 0.0079 | 0.0079 |
| **Leu** | 0.1385 | 0.9902 | 0.5107 | 0.0349 | 0.1454 | 0.0457 | 0.0079 | 0.0079 | 0.0079 |
| **Lys*** | 0.2019 | 0 | 0.2956 | 0.0732 | 0 | 0.1892 | 0.0112 | 0.5476 | 0.0112 |
| **Phe*** | 0.07 | 0.1876 | 0.0978 | 0.0266 | 0.0289 | 0.0249 | 0.0119 | 0.2222 | 0.0119 |
| **Pro** | 0.3659 | 1.5386 | 0.8662 | 0.0332 | 0.361 | 0.0785 | 0.0079 | 0.0079 | 0.0079 |
| **Ser*** | 0.4347 | 1.7985 | 0.5412 | 0.2656 | 0.2451 | 0.1008 | 0.0119 | 0.4206 | 0.0119 |
| **Thr** | 0.8484 | 1.9682 | 1.6958 | 0.1589 | 0.4244 | 0.2334 | 0.0119 | 0.0119 | 0.2222 |
| **Tyr** | 0.4702 | 0.5169 | 0.8284 | 0.1167 | 0.067 | 0.1361 | 0.5476 | 0.0119 | 0.0119 |
| **Val** | 0.1819 | 1.4824 | 0.5948 | 0.0481 | 0.235 | 0.0343 | 0.0079 | 0.0079 | 0.0079 |
| *Phenolic acids* |  |  |  |  |  |  |  |  |  |
| **caftaric acid** | 0.0102 | 0.0007 | 0.0028 | 0.0025 | 0.0003 | 0.0004 | 0.0079 | 0.0079 | 0.0079 |
| **caffeic acid** | 0.0004 | 0.0002 | 0.0003 | 0.0001 | 0.0001 | 0.0001 | 0.0476 | 0.1508 | 0.0833 |
| **gallic acid** | 0.0406 | 0.0288 | 0.0268 | 0.0053 | 0.0039 | 0.0038 | 0.0119 | 0.0119 | 0.8413 |
| **isoferulic acid*** | 0.0002 | 0.0007 | 0.0003 | 0.0001 | 0.0001 | 0.0002 | 0.0119 | 0.8413 | 0.0119 |
| *Flavonols* |  |  |  |  |  |  |  |  |  |
| **quercetin 3 glucoside** | 0.0101 | 0.0026 | 0.0012 | 0.0017 | 0.0005 | 0.0004 | 0.0079 | 0.0079 | 0.0079 |
| **quercetin 3 glucuronide** | 0.0113 | 0.002 | 0.0017 | 0.0021 | 0.0005 | 0.0007 | 0.0119 | 0.0119 | 0.3095 |
| **kaempferol 3 glucoside** | 0.0036 | 0.0006 | 0.0001 | 0.0009 | 0.0003 | 0.0001 | 0.0079 | 0.0079 | 0.0079 |
| *Flavanols* |  |  |  |  |  |  |  |  |  |
| **catechin*** | 0.3163 | 0.079 | 0.3328 | 0.0398 | 0.005 | 0.0389 | 0.0119 | 0.6905 | 0.0119 |
| **epicatechin** | 0.2507 | 0.013 | 0.0433 | 0.0147 | 0.0033 | 0.0021 | 0.0079 | 0.0079 | 0.0079 |
| **epicatechin gallate** | 0.1336 | 0.0357 | 0.0457 | 0.0121 | 0.0035 | 0.0044 | 0.0119 | 0.0119 | 0.0159 |
| **epigallocatechin** | 0.009 | 0.0005 | 0.0013 | 0.0017 | 0.0002 | 0.0002 | 0.0079 | 0.0079 | 0.0079 |
| **epigallocatechin gallate** | 0.0162 | 0.0021 | 0.0061 | 0.0016 | 0.0002 | 0.0005 | 0.0079 | 0.0079 | 0.0079 |
| **gallocatechin** | 0.0104 | 0.0006 | 0.0015 | 0.0024 | 0.0001 | 0.0005 | 0.0119 | 0.0119 | 0.0159 |
| **B1** | 0.3997 | 0.0454 | 0.1694 | 0.0374 | 0.005 | 0.024 | 0.0079 | 0.0079 | 0.0079 |
| **B2** | 0.1225 | 0.0109 | 0.0216 | 0.0119 | 0.0022 | 0.0039 | 0.0079 | 0.0079 | 0.0079 |
| **B3** | 0.1219 | 0.0166 | 0.051 | 0.011 | 0.0032 | 0.0052 | 0.0079 | 0.0079 | 0.0079 |
| **B4** | 0.0795 | 0.0042 | 0.0171 | 0.0056 | 0.001 | 0.0037 | 0.0079 | 0.0079 | 0.0079 |
| **B1 gallate** | 0.2193 | 0.0525 | 0.0385 | 0.0092 | 0.0081 | 0.0027 | 0.0079 | 0.0079 | 0.0079 |
| **C1** | 0.1275 | 0.0257 | 0.0275 | 0.0105 | 0.0151 | 0.003 | 0.0119 | 0.0119 | 0.6905 |
| **trimer1** | 0.1842 | 0.0481 | 0.0956 | 0.0065 | 0.0123 | 0.0162 | 0.0079 | 0.0079 | 0.0079 |
| *Stilbenes* |  |  |  |  |  |  |  |  |  |
| ***trans*-resveratrol** | 0.0506 | 0.0677 | 0.0511 | 0.0183 | 0.0021 | 0.0157 | 0.2262 | 0.8413 | 0.2262 |
| ***trans*-piceid** | 0.0508 | 0.0198 | 0.0203 | 0.0034 | 0.0036 | 0.0016 | 0.0119 | 0.0119 | 0.8413 |
| ***cis*-piceid*** | 0.0011 | 0.0026 | 0.0008 | 0.0004 | 0.0005 | 0.0003 | 0.0119 | 0.4206 | 0.0119 |
| ***trans*-piceatannol** | 0.0276 | 0.0356 | 0.0344 | 0.0059 | 0.007 | 0.0063 | 0.2262 | 0.2262 | 0.8413 |
| ***trans*-astringin** | 0.0042 | 0.0024 | 0.0026 | 0.0004 | 0.0003 | 0.0005 | 0.0119 | 0.0119 | 0.8413 |
| ***cis*-astringin** | 0.2349 | 0.0209 | 0.011 | 0.0135 | 0.0026 | 0.0011 | 0.0079 | 0.0079 | 0.0079 |
| ***trans-isorhapotin**** | 0.0008 | 0.0005 | 0.0005 | 0.0002 | 0.0001 | 0.0002 | 0.0238 | 0.0476 | 1 |
| ***trans*-ε-viniferin** | 0.606 | 0.9225 | 1.108 | 0.0511 | 0.0859 | 0.1523 | 0.0119 | 0.0119 | 0.0317 |
| ***trans-*ω-viniferin** | 0.0151 | 0.0455 | 0.0387 | 0.004 | 0.0081 | 0.0057 | 0.0119 | 0.0119 | 0.1508 |
| ***cis*-ε-viniferin** | 0.0148 | 0.0207 | 0.0254 | 0.009 | 0.0076 | 0.0063 | 0.2222 | 0.2222 | 0.2222 |
| ***trans-*δ*-*viniferin** | 0.0189 | 0.0164 | 0.0177 | 0.0018 | 0.0008 | 0.0023 | 0.0476 | 0.631 | 0.8413 |
| **pallidol** | 0.0233 | 0.0564 | 0.0375 | 0.0032 | 0.0057 | 0.0085 | 0.0079 | 0.0079 | 0.0079 |
| **parthenocisin A*** | 0.0076 | 0.0243 | 0.0093 | 0.0012 | 0.004 | 0.0026 | 0.0119 | 0.4206 | 0.0119 |
| **ampelopsin A** | 0.0348 | 0.0116 | 0.0075 | 0.0111 | 0.0034 | 0.0022 | 0.0119 | 0.0119 | 0.0556 |
| **vitisinol C** | 0.0028 | 0.0016 | 0.0037 | 0.0004 | 0.0002 | 0.0004 | 0.0079 | 0.0079 | 0.0079 |
| **dimer diglycoside** | 0.0046 | 0.004 | 0.0044 | 0.0005 | 0.0004 | 0.0007 | 0.4524 | 0.5476 | 0.4643 |
| **dimer glycoside A** | 0.0027 | 0.0026 | 0.0027 | 0.0002 | 0.0003 | 0.0004 | 0.631 | 0.631 | 0.6905 |
| **dimer glycoside B** | 0.0055 | 0.0029 | 0.005 | 0.0017 | 0.0004 | 0.0005 | 0.0833 | 0.2222 | 0.0238 |
| **dimer glycoside C** | 0.0043 | 0.0047 | 0.0067 | 0.0002 | 0.0009 | 0.0005 | 0.3095 | 0.0119 | 0.0119 |
| **α-viniferin*** | 0.0661 | 0.2386 | 0.0734 | 0.0082 | 0.0297 | 0.0169 | 0.0119 | 0.6905 | 0.0119 |
| **miyabenol C*** | 0.0548 | 0.1879 | 0.0422 | 0.007 | 0.03 | 0.0105 | 0.0119 | 0.0952 | 0.0119 |
| **hopeaphenol** | 0.2158 | 0.3212 | 0.3882 | 0.0204 | 0.034 | 0.0648 | 0.0119 | 0.0119 | 0.0556 |
| **isohopeaphenol** | 0.1547 | 0.4985 | 0.5113 | 0.0164 | 0.0579 | 0.1081 | 0.0119 | 0.0119 | 1 |
| **r2-viniferin** | 0.0078 | 0.0054 | 0.0109 | 0.0009 | 0.0011 | 0.0021 | 0.0119 | 0.0317 | 0.0119 |
| **r-viniferin** | 0.0583 | 0.0469 | 0.0971 | 0.0086 | 0.0101 | 0.0163 | 0.0952 | 0.0119 | 0.0119 |
| *Others* |  |  |  |  |  |  |  |  |  |
| **naringenin*** | 0.0008 | 0.003 | 0.0005 | 0.0002 | 0.0003 | 0.0002 | 0.0119 | 0.0556 | 0.0119 |
| **naringenin glucoside** | 1.8059 | 0.098 | 0.0224 | 0.1088 | 0.0134 | 0.0077 | 0.0079 | 0.0079 | 0.0079 |
| **phloretin** | 0.001 | 0.0004 | 0.0018 | 0.0002 | 0.0002 | 0.0002 | 0.0079 | 0.0079 | 0.0079 |
| **taxifolin** | 0.001 | 0.0028 | 0.0006 | 0.0001 | 0.0002 | 0.0001 | 0.0079 | 0.0079 | 0.0079 |

Supplementary table 6: Metabolite concentration, protein content and percentage of water in the scion wood, graft interface and rootstock wood of hetero-grafts of Vitis vinifera cv. Négrette onto V. berlandieri x V. ruprestris cv. 140 Ruggeri, 33 days after grafting. Mean concentrations and standard deviation shown (n = 5). p-value of Wilcoxon-test between scion and interface tissues (S – I), scion and rootstock tissues (S – R), and interface and rootstock tissues (I -R). Stars indicates a significant difference between S – I and between I – R, but no significant difference between S - R. Significance threshold set at p-value < 0.05.

| **Variables** | **Mean** | | | **standard deviation** | | | ***p-value* wilcoxon-test** | | |
| --- | --- | --- | --- | --- | --- | --- | --- | --- | --- |
|  | Scion | Interface | Rootstock | Scion | Interface | Rootstock | S - I | S - R | I - R |
| **% of water*** | 57.9387 | 72.1637 | 56.5122 | 0.6084 | 1.5671 | 1.3361 | 0.0119 | 0.1161 | 0.0119 |
| **starch** | 76.7712 | 43.0092 | 104.707 | 9.03 | 6.572 | 8.7351 | 0.0079 | 0.0079 | 0.0079 |
| **proteins** | 19.0959 | 31.8113 | 31.5029 | 2.9656 | 4.976 | 5.9747 | 0.0119 | 0.0119 | 0.8413 |
| **sucrose** | 1.1353 | 1.8277 | 1.8533 | 0.5273 | 1.1945 | 0.3239 | 0.3333 | 0.0952 | 1 |
| **glucose** | 0.8542 | 1.547 | 1.0607 | 0.5042 | 0.2188 | 0.5045 | 0.1667 | 0.5476 | 0.2262 |
| **fructose*** | 0.8942 | 3.3917 | 1.4765 | 0.357 | 0.7985 | 0.3139 | 0.0119 | 0.0556 | 0.0119 |
| *Amino acids* |  |  |  |  |  |  |  |  |  |
| **Ala*** | 0.2907 | 0.9578 | 0.4029 | 0.1087 | 0.1115 | 0.1098 | 0.0119 | 0.1508 | 0.0119 |
| **Arg** | 3.3977 | 2.8759 | 3.8704 | 0.4353 | 0.5778 | 1.4752 | 0.4524 | 0.8413 | 0.4643 |
| **Asn** | 1.4788 | 13.4235 | 5.3546 | 0.7053 | 2.4117 | 2.0076 | 0.0079 | 0.0079 | 0.0079 |
| **Asp** | 1.6515 | 2.8035 | 2.4515 | 0.4199 | 0.3993 | 0.7516 | 0.0238 | 0.1429 | 0.3095 |
| **GABA** | 0.539 | 1.5679 | 1.0775 | 0.1897 | 0.1388 | 0.2181 | 0.0119 | 0.0159 | 0.0119 |
| **Gln*** | 6.3119 | 19.4951 | 9.3475 | 1.673 | 2.1057 | 2.8353 | 0.0119 | 0.0556 | 0.0119 |
| **Glu** | 2.4538 | 2.7954 | 3.003 | 0.5392 | 1.5331 | 0.8307 | 0.5476 | 0.5476 | 0.5476 |
| **Gly** | 0.0528 | 0.1178 | 0.0151 | 0.0921 | 0.1833 | 0.0163 | 0.7887 | 1 | 0.7887 |
| **His** | 0.8631 | 0.53 | 0.6382 | 0.0901 | 0.3174 | 0.1315 | 0.0476 | 0.0476 | 0.6905 |
| **Ile** | 0.1408 | 0.3406 | 0.2055 | 0.0177 | 0.1145 | 0.0694 | 0.0238 | 0.1508 | 0.0833 |
| **Leu** | 0.1038 | 0.159 | 0.1451 | 0.0207 | 0.0797 | 0.0445 | 0.1429 | 0.1429 | 1 |
| **Lys** | 0.1965 | 0.102 | 0.1952 | 0.0487 | 0.0324 | 0.11 | 0.0238 | 0.6905 | 0.2262 |
| **Phe** | 0.0654 | 0.1052 | 0.0998 | 0.0092 | 0.0339 | 0.0311 | 0.0238 | 0.0476 | 1 |
| **Pro** | 0.4273 | 0.8138 | 0.5337 | 0.0796 | 0.2582 | 0.1627 | 0.0238 | 0.4206 | 0.1429 |
| **Ser*** | 0.3398 | 1.244 | 0.5546 | 0.1955 | 0.3194 | 0.1858 | 0.0119 | 0.0952 | 0.0119 |
| **Thr** | 0.8967 | 1.093 | 1.125 | 0.2512 | 0.1554 | 0.3249 | 0.3333 | 0.3333 | 1 |
| **Tyr** | 0.5868 | 0.3356 | 0.7851 | 0.061 | 0.0608 | 0.1371 | 0.0079 | 0.0079 | 0.0079 |
| **Val** | 0.1849 | 0.4815 | 0.2831 | 0.0315 | 0.1243 | 0.0749 | 0.0238 | 0.0317 | 0.0317 |
| *Phenolic acids* |  |  |  |  |  |  |  |  |  |
| **caftaric acid** | 0.0079 | 0.0003 | 0.0035 | 0.0019 | 0.0001 | 0.0019 | 0.0119 | 0.0317 | 0.0119 |
| **caffeic acid*** | 0.0006 | 0.0001 | 0.0008 | 0.0002 | 0.0001 | 0.0002 | 0.0119 | 0.1508 | 0.0119 |
| **gallic acid** | 0.0481 | 0.0279 | 0.0348 | 0.0056 | 0.0024 | 0.0021 | 0.0079 | 0.0079 | 0.0079 |
| **isoferulic acid*** | 0.0003 | 0.0009 | 0.0003 | 0.0001 | 0.0002 | 0.0001 | 0.0119 | 0.3095 | 0.0119 |
| *Flavonols* |  |  |  |  |  |  |  |  |  |
| **quercetin 3 glucoside** | 0.0016 | 0.0012 | 0.0008 | 0.0014 | 0.0003 | 0.0003 | 0.5476 | 0.5476 | 0.1667 |
| **quercetin 3 glucuronide** | 0.001 | 0.0018 | 0.0062 | 0.0008 | 0.0009 | 0.0026 | 0.2222 | 0.0119 | 0.0119 |
| **kaempferol 3 glucoside** | 0.0002 | 0.0001 | 0.0001 | 0.0002 | 0.0001 | 0 | 0.5476 | 0.5476 | 0.5476 |
| *Flavanols* |  |  |  |  |  |  |  |  |  |
| **catechin*** | 0.1613 | 0.0513 | 0.216 | 0.0369 | 0.0073 | 0.0304 | 0.0119 | 0.0952 | 0.0119 |
| **epicatechin** | 0.0442 | 0.0049 | 0.0222 | 0.0131 | 0.0008 | 0.0083 | 0.0119 | 0.0159 | 0.0119 |
| **epicatechin gallate** | 0.062 | 0.0147 | 0.0403 | 0.0066 | 0.0017 | 0.004 | 0.0079 | 0.0079 | 0.0079 |
| **epigallocatechin** | 0.0107 | 0.0008 | 0.0022 | 0.0016 | 0.0001 | 0.0009 | 0.0079 | 0.0079 | 0.0079 |
| **epigallocatechin gallate** | 0.0188 | 0.0014 | 0.0102 | 0.0035 | 0.0005 | 0.0026 | 0.0079 | 0.0079 | 0.0079 |
| **gallocatechin** | 0.0114 | 0.0008 | 0.002 | 0.0012 | 0.0004 | 0.0009 | 0.0119 | 0.0119 | 0.0952 |
| **B1*** | 0.1495 | 0.0227 | 0.1461 | 0.0097 | 0.0038 | 0.015 | 0.0119 | 0.8413 | 0.0119 |
| **B2** | 0.0503 | 0.0064 | 0.0337 | 0.0019 | 0.0013 | 0.0022 | 0.0079 | 0.0079 | 0.0079 |
| **B3*** | 0.0557 | 0.0074 | 0.0444 | 0.0033 | 0.0007 | 0.0062 | 0.0119 | 0.0556 | 0.0119 |
| **B4** | 0.0231 | 0.0022 | 0.017 | 0.0017 | 0.0005 | 0.0037 | 0.0079 | 0.0079 | 0.0079 |
| **B1 gallate** | 0.1855 | 0.077 | 0.1006 | 0.0466 | 0.0174 | 0.0261 | 0.0119 | 0.0119 | 0.1508 |
| **C1*** | 0.0566 | 0.0107 | 0.0501 | 0.0043 | 0.0031 | 0.0242 | 0.0119 | 0.1508 | 0.0119 |
| **trimer1*** | 0.0847 | 0.0317 | 0.0968 | 0.0112 | 0.0068 | 0.0251 | 0.0119 | 0.6905 | 0.0119 |
| *Stilbenes* |  |  |  |  |  |  |  |  |  |
| ***trans*-resveratrol** | 0.0357 | 0.0205 | 0.033 | 0.009 | 0.0094 | 0.0106 | 0.0952 | 1 | 0.1429 |
| ***trans*-piceid** | 0.0187 | 0.0097 | 0.0327 | 0.0026 | 0.0025 | 0.0056 | 0.0079 | 0.0079 | 0.0079 |
| ***cis*-piceid** | 0.0006 | 0.0022 | 0.0015 | 0.0001 | 0.0004 | 0.0006 | 0.0119 | 0.0119 | 0.0556 |
| ***trans*-piceatannol** | 0.0215 | 0.0124 | 0.0246 | 0.0062 | 0.0041 | 0.0057 | 0.1429 | 0.6905 | 0.0238 |
| ***trans*-astringin** | 0.0023 | 0.0017 | 0.0043 | 0.0005 | 0.0004 | 0.0007 | 0.0556 | 0.0119 | 0.0119 |
| ***cis*-astringin** | 0.028 | 0.0042 | 0.0128 | 0.0032 | 0.0009 | 0.0022 | 0.0079 | 0.0079 | 0.0079 |
| ***trans-isorhapotin**** | 0.0005 | 0.0003 | 0.0004 | 0.0002 | 0.0001 | 0.0001 | 0.0952 | 0.5476 | 0.5476 |
| ***trans*-ε-viniferin** | 1.2988 | 0.7912 | 0.7957 | 0.0624 | 0.1106 | 0.1385 | 0.0119 | 0.0119 | 0.8413 |
| ***trans-*ω-viniferin** | 0.0599 | 0.0447 | 0.0405 | 0.0032 | 0.0095 | 0.0064 | 0.0833 | 0.0238 | 0.3095 |
| ***cis*-ε-viniferin** | 0.0369 | 0.0351 | 0.0279 | 0.0286 | 0.0266 | 0.0153 | 1 | 1 | 1 |
| ***trans-*δ*-*viniferin** | 0.0213 | 0.0082 | 0.0059 | 0.0025 | 0.0015 | 0.0024 | 0.0119 | 0.0119 | 0.1508 |
| **pallidol** | 0.0244 | 0.0444 | 0.0284 | 0.0045 | 0.013 | 0.0129 | 0.0238 | 1 | 0.2262 |
| **parthenocisin A*** | 0.0102 | 0.0267 | 0.0111 | 0.0029 | 0.0075 | 0.0053 | 0.0119 | 1 | 0.0119 |
| **ampelopsin A** | 0.1265 | 0.0302 | 0.0191 | 0.0088 | 0.0045 | 0.0187 | 0.0119 | 0.0119 | 0.1508 |
| **vitisinol C** | 0.0081 | 0.0022 | 0.002 | 0.0013 | 0.0003 | 0.0007 | 0.0119 | 0.0119 | 0.3095 |
| **dimer diglycoside** | 0.0043 | 0.0048 | 0.01 | 0.0014 | 0.0017 | 0.0042 | 0.6905 | 0.0952 | 0.1429 |
| **dimer glycoside A** | 0.002 | 0.0022 | 0.005 | 0.0003 | 0.0004 | 0.0015 | 0.4206 | 0.0119 | 0.0119 |
| **dimer glycoside B** | 0.011 | 0.0021 | 0.0012 | 0.0018 | 0.0002 | 0.0002 | 0.0079 | 0.0079 | 0.0079 |
| **dimer glycoside C** | 0.0052 | 0.0021 | 0.0032 | 0.0004 | 0.0003 | 0.0008 | 0.0119 | 0.0119 | 0.0556 |
| **α-viniferin** | 0.0791 | 0.1667 | 0.0279 | 0.0178 | 0.0371 | 0.0127 | 0.0079 | 0.0079 | 0.0079 |
| **miyabenol C** | 0.2592 | 0.1197 | 0.0268 | 0.0238 | 0.0294 | 0.0083 | 0.0079 | 0.0079 | 0.0079 |
| **hopeaphenol** | 0.1734 | 0.1039 | 0.1234 | 0.0401 | 0.0319 | 0.0718 | 0.0476 | 0.2262 | 1 |
| **isohopeaphenol** | 0.1182 | 0.2822 | 0.2503 | 0.0618 | 0.1095 | 0.1354 | 0.0952 | 0.1429 | 0.5476 |
| **r2-viniferin** | 0.0043 | 0.0061 | 0.0249 | 0.0011 | 0.0015 | 0.0044 | 0.0556 | 0.0119 | 0.0119 |
| **r-viniferin** | 0.0371 | 0.0556 | 0.1561 | 0.018 | 0.0115 | 0.0186 | 0.2222 | 0.0119 | 0.0119 |
| *Others* |  |  |  |  |  |  |  |  |  |
| **naringenin** | 0.0001 | 0.0009 | 0.0005 | 0 | 0.0001 | 0.0001 | 0.0079 | 0.0079 | 0.0079 |
| **naringenin glucoside** | 0.192 | 0.0122 | 0.0088 | 0.0336 | 0.005 | 0.0045 | 0.0119 | 0.0119 | 0.2222 |
| **phloretin** | 0.0004 | 0.0002 | 0.0014 | 0.0001 | 0.0001 | 0.0002 | 0.0079 | 0.0079 | 0.0079 |
| **taxifolin** | 0.0002 | 0.0016 | 0.0006 | 0.0001 | 0.0001 | 0.0002 | 0.0079 | 0.0079 | 0.0079 |

Supplementary table 7: Metabolite concentration, protein content and percentage of water in the scion wood, graft interface and rootstock wood of homo-grafts of Vitis vinifera cv. Négrette 33 days after grafting. Mean concentrations and standard deviation shown (n = 5). p-value of Wilcoxon-test between scion and interface tissues (S – I), scion and rootstock tissues (S – R), and interface and rootstock tissues (I -R). Stars indicates a significant difference between S – I and between I – R, but no significant difference between S - R. Significance threshold set at p-value < 0.05.

| **Variables** | **Mean** | | | **standard deviation** | | | ***p-value* wilcoxon-test** | | |
| --- | --- | --- | --- | --- | --- | --- | --- | --- | --- |
|  | Scion | Interface | Rootstock | Scion | Interface | Rootstock | S - I | S - R | I - R |
| **% of water*** | 59.0749 | 70.5008 | 58.7361 | 1.8317 | 1.9447 | 0.8909 | 0.0238 | 0.9048 | 0.0238 |
| **starch*** | 81.3071 | 46.0027 | 79.0239 | 9.0469 | 17.4553 | 9.3363 | 0.0238 | 0.9048 | 0.0238 |
| **proteins** | 20.2045 | 26.6492 | 22.8269 | 2.0498 | 7.7 | 3.5665 | 0.2857 | 0.2857 | 0.2857 |
| **sucrose** | 1.4234 | 1.4716 | 1.4519 | 0.2962 | 0.8921 | 0.2624 | 0.5556 | 0.5556 | 0.5556 |
| **glucose** | 0.8864 | 1.1356 | 0.9644 | 0.4471 | 0.3329 | 0.2533 | 0.9048 | 0.9048 | 0.9048 |
| **fructose*** | 1.2933 | 1.9955 | 1.3191 | 0.1275 | 0.5548 | 0.1899 | 0.0238 | 0.9048 | 0.0476 |
| *Amino acids* |  |  |  |  |  |  |  |  |  |
| **Ala** | 0.3435 | 1.0129 | 0.6224 | 0.0521 | 0.1928 | 0.1556 | 0.0238 | 0.0238 | 0.0317 |
| **Arg** | 3.6616 | 4.9789 | 4.9677 | 0.8656 | 1.8139 | 0.5534 | 0.631 | 0.1905 | 0.7302 |
| **Asn*** | 1.1331 | 11.2357 | 2.437 | 0.4742 | 8.3692 | 1.1382 | 0.0238 | 0.0635 | 0.0238 |
| **Asp** | 1.6972 | 2.5422 | 2.1595 | 0.2399 | 0.9987 | 0.3353 | 0.1429 | 0.1429 | 0.5556 |
| **GABA** | 0.8528 | 1.7703 | 1.5606 | 0.1311 | 0.4051 | 0.4511 | 0.0238 | 0.0238 | 0.7302 |
| **Gln** | 7.1825 | 24.2662 | 10.7508 | 1.7804 | 6.5302 | 2.0356 | 0.0238 | 0.0317 | 0.0238 |
| **Glu** | 2.6273 | 2.988 | 3.559 | 0.2874 | 0.733 | 0.2258 | 0.6905 | 0.0476 | 0.4286 |
| **Gly** | 0.0094 | 0.0975 | 0.0437 | 0.0126 | 0.1177 | 0.0256 | 0.3068 | 0.3068 | 0.7302 |
| **His** | 0.257 | 0.2655 | 0.5837 | 0.2425 | 0.3364 | 0.1642 | 1 | 0.1648 | 0.1648 |
| **Ile** | 0.1651 | 0.6599 | 0.2728 | 0.0391 | 0.6643 | 0.0631 | 0.0238 | 0.0952 | 0.1111 |
| **Leu** | 0.1425 | 0.338 | 0.2204 | 0.0343 | 0.3868 | 0.033 | 0.3333 | 0.0952 | 0.4127 |
| **Lys** | 0.1617 | 0.1188 | 0.2278 | 0.0361 | 0.0949 | 0.0425 | 0.3095 | 0.0952 | 0.0952 |
| **Phe** | 0.0509 | 0.0885 | 0.071 | 0.0085 | 0.0527 | 0.0063 | 0.0833 | 0.0476 | 1 |
| **Pro** | 0.6967 | 0.8976 | 0.9811 | 0.1365 | 0.2101 | 0.1708 | 0.2262 | 0.1905 | 0.7302 |
| **Ser** | 0.3585 | 1.221 | 0.5677 | 0.0763 | 0.0737 | 0.0901 | 0.0238 | 0.0317 | 0.0238 |
| **Thr** | 0.7494 | 1.1309 | 1.1574 | 0.1452 | 0.472 | 0.1022 | 0.1429 | 0.0476 | 0.1905 |
| **Tyr** | 0.5064 | 0.2934 | 0.6481 | 0.067 | 0.1557 | 0.1319 | 0.1111 | 0.1111 | 0.1111 |
| **Val** | 0.2609 | 0.6846 | 0.4113 | 0.0805 | 0.3801 | 0.0481 | 0.0238 | 0.0238 | 0.0317 |
| *Phenolic acids* |  |  |  |  |  |  |  |  |  |
| **caftaric acid*** | 0.0064 | 0.0003 | 0.0054 | 0.0016 | 0.0002 | 0.0019 | 0.0238 | 0.5556 | 0.0238 |
| **caffeic acid** | 0.0002 | 0.0001 | 0.0002 | 0.0001 | 0 | 0.0001 | 0.9048 | 0.9048 | 0.0952 |
| **gallic acid*** | 0.0322 | 0.0179 | 0.0316 | 0.0063 | 0.0007 | 0.0033 | 0.0238 | 0.5556 | 0.0238 |
| **isoferulic acid** | 0.0001 | 0.0003 | 0.0002 | 0.0001 | 0.0002 | 0 | 0.0238 | 0.0952 | 0.1905 |
| *Flavonols* |  |  |  |  |  |  |  |  |  |
| **quercetin 3 glucoside*** | 0.0012 | 0.0023 | 0.0013 | 0.0001 | 0.0005 | 0.0001 | 0.0238 | 0.2857 | 0.0238 |
| **quercetin 3 glucuronide** | 0.0004 | 0.0003 | 0.0004 | 0.0002 | 0.0002 | 0.0001 | 0.631 | 0.9048 | 0.631 |
| **kaempferol 3 glucoside*** | 0.0001 | 0.0002 | 0.0001 | 0 | 0.0001 | 0 | 0.0238 | 0.5556 | 0.0238 |
| *Flavanols* |  |  |  |  |  |  |  |  |  |
| **Catechin*** | 0.1463 | 0.0481 | 0.1402 | 0.037 | 0.0131 | 0.0149 | 0.0238 | 0.9048 | 0.0238 |
| **epicatechin*** | 0.0523 | 0.0067 | 0.0448 | 0.0177 | 0.0018 | 0.0167 | 0.0238 | 0.5556 | 0.0238 |
| **epicatechin gallate*** | 0.0603 | 0.0179 | 0.0599 | 0.0067 | 0.0063 | 0.0123 | 0.0238 | 1 | 0.0238 |
| **epigallocatechin*** | 0.0106 | 0.0012 | 0.0081 | 0.0029 | 0.0005 | 0.0035 | 0.0238 | 0.1905 | 0.0238 |
| **epigallocatechin gallate*** | 0.0252 | 0.0043 | 0.0241 | 0.0039 | 0.0026 | 0.005 | 0.0238 | 0.9048 | 0.0238 |
| **gallocatechin*** | 0.0097 | 0.0008 | 0.0079 | 0.0029 | 0.0005 | 0.0035 | 0.0238 | 0.4127 | 0.0238 |
| **B1*** | 0.1243 | 0.0298 | 0.128 | 0.0175 | 0.0102 | 0.0269 | 0.0238 | 0.9048 | 0.0238 |
| **B2*** | 0.038 | 0.0092 | 0.0365 | 0.0051 | 0.0043 | 0.0092 | 0.0238 | 0.5556 | 0.0238 |
| **B3*** | 0.0379 | 0.0087 | 0.0425 | 0.0063 | 0.0022 | 0.0059 | 0.0238 | 0.4127 | 0.0238 |
| **B4*** | 0.0177 | 0.0029 | 0.015 | 0.0026 | 0.0008 | 0.0051 | 0.0238 | 0.4127 | 0.0238 |
| **B1 gallate*** | 0.1787 | 0.0814 | 0.1609 | 0.0261 | 0.0376 | 0.0357 | 0.0238 | 0.1905 | 0.0238 |
| **C1** | 0.047 | 0.0146 | 0.0622 | 0.0243 | 0.0063 | 0.0192 | 0.0833 | 0.2857 | 0.0476 |
| **trimer1** | 0.0706 | 0.0377 | 0.0812 | 0.0321 | 0.0128 | 0.019 | 0.2262 | 0.7302 | 0.0476 |
| *Stilbenes* |  |  |  |  |  |  |  |  |  |
| ***trans*-resveratrol** | 0.0234 | 0.0242 | 0.0193 | 0.0056 | 0.009 | 0.0025 | 0.8413 | 0.5714 | 0.8333 |
| ***trans*-piceid*** | 0.0146 | 0.0057 | 0.0134 | 0.0018 | 0.0016 | 0.0017 | 0.0238 | 0.2857 | 0.0238 |
| ***cis*-piceid*** | 0.0004 | 0.0021 | 0.0006 | 0.0001 | 0.0006 | 0.0002 | 0.0238 | 0.1111 | 0.0238 |
| ***trans*-piceatannol** | 0.0145 | 0.0154 | 0.0105 | 0.0034 | 0.0049 | 0.0032 | 1 | 0.2857 | 0.2857 |
| ***trans*-astringin*** | 0.0017 | 0.0011 | 0.0017 | 0.0002 | 0.0002 | 0.0003 | 0.0238 | 0.7302 | 0.0476 |
| ***cis*-astringin*** | 0.018 | 0.0042 | 0.016 | 0.002 | 0.001 | 0.0028 | 0.0238 | 0.1905 | 0.0238 |
| ***trans-isorhapotin**** | 0.0003 | 0.0004 | 0.0004 | 0.0001 | 0.0002 | 0.0002 | 0.619 | 0.619 | 1 |
| ***trans*-ε-viniferin*** | 1.0844 | 0.8719 | 1.1347 | 0.1257 | 0.1054 | 0.0599 | 0.0476 | 0.7302 | 0.0476 |
| ***trans-*ω-viniferin** | 0.0425 | 0.0427 | 0.0466 | 0.0058 | 0.0037 | 0.0049 | 1 | 0.4286 | 0.4286 |
| ***cis*-ε-viniferin** | 0.0177 | 0.0138 | 0.017 | 0.0044 | 0.003 | 0.006 | 0.7302 | 0.7302 | 0.7302 |
| ***trans-*δ*-*viniferin*** | 0.0202 | 0.0126 | 0.0204 | 0.0026 | 0.0012 | 0.0027 | 0.0238 | 1 | 0.0238 |
| **pallidol*** | 0.0263 | 0.0388 | 0.0257 | 0.0048 | 0.0054 | 0.005 | 0.0238 | 0.9048 | 0.0238 |
| **parthenocisin A** | 0.0074 | 0.0224 | 0.011 | 0.0008 | 0.006 | 0.0025 | 0.0238 | 0.0238 | 0.0317 |
| **ampelopsin A*** | 0.118 | 0.0573 | 0.1219 | 0.0371 | 0.0309 | 0.0151 | 0.0238 | 0.5556 | 0.0238 |
| **vitisinol C*** | 0.0106 | 0.0049 | 0.0102 | 0.0011 | 0.0018 | 0.0013 | 0.0238 | 0.9048 | 0.0238 |
| **dimer diglycoside** | 0.0032 | 0.0029 | 0.0029 | 0.0005 | 0.0007 | 0.0003 | 0.619 | 0.619 | 0.7302 |
| **dimer glycoside A** | 0.0019 | 0.0019 | 0.0019 | 0.0003 | 0.0006 | 0.0003 | 0.9048 | 0.9048 | 0.9048 |
| **dimer glycoside B*** | 0.0094 | 0.0042 | 0.0086 | 0.0016 | 0.0014 | 0.0022 | 0.0238 | 0.5556 | 0.0238 |
| **dimer glycoside C*** | 0.0042 | 0.0028 | 0.0043 | 0.0006 | 0.0005 | 0.0006 | 0.0238 | 0.7302 | 0.0238 |
| **α-viniferin*** | 0.1121 | 0.247 | 0.1447 | 0.0161 | 0.044 | 0.0251 | 0.0238 | 0.0635 | 0.0238 |
| **miyabenol C** | 0.209 | 0.1718 | 0.2259 | 0.0403 | 0.0348 | 0.0062 | 0.631 | 0.7302 | 0.0476 |
| **hopeaphenol** | 0.1425 | 0.1246 | 0.1312 | 0.0209 | 0.0682 | 0.0221 | 0.2857 | 0.5556 | 0.2857 |
| **isohopeaphenol** | 0.0536 | 0.1186 | 0.0736 | 0.0034 | 0.0773 | 0.0126 | 0.0238 | 0.0476 | 0.2857 |
| **r2-viniferin** | 0.0041 | 0.0029 | 0.0041 | 0.0024 | 0.0011 | 0.0011 | 0.5556 | 0.5556 | 0.3333 |
| **r-viniferin** | 0.025 | 0.0195 | 0.0134 | 0.0215 | 0.0152 | 0.0148 | 0.8214 | 0.5714 | 0.9048 |
| *Others* |  |  |  |  |  |  |  |  |  |
| **naringenin** | 0.0001 | 0.0003 | 0.0001 | 0 | 0.0002 | 0 | 0.2857 | 0.2857 | 0.1905 |
| **naringenin glucoside*** | 0.1666 | 0.0836 | 0.139 | 0.0281 | 0.0428 | 0.0221 | 0.0238 | 0.1111 | 0.0476 |
| **phloretin*** | 0.0004 | 0.0002 | 0.0004 | 0.0001 | 0.0001 | 0.0001 | 0.0476 | 0.7302 | 0.0476 |
| **taxifolin** | 0.0002 | 0.0005 | 0.0002 | 0 | 0.0005 | 0.0001 | 0.0476 | 0.4127 | 0.0952 |

Supplementary table 8: Metabolite concentration, protein content and percentage of water in the scion wood, graft interface and rootstock wood of hetero-grafts of Vitis vinifera cv. Négrette onto Vitis berlandieri x Vitis riparia cv. Rességuier Sélection Birolleau 1, 33 days after grafting. Mean concentrations and standard deviation shown (n = 5). p-value of Wilcoxon-test between scion and interface tissues (S – I), scion and rootstock tissues (S – R), and interface and rootstock tissues (I -R). Stars indicates a significant difference between S – I and between I – R, but no significant difference between S - R. Significance threshold set at p-value < 0.05.

| **Variables** | **Mean** | | | **standard deviation** | | | ***p-value* wilcoxon-test** | | |
| --- | --- | --- | --- | --- | --- | --- | --- | --- | --- |
|  | Scion | Interface | Rootstock | Scion | Interface | Rootstock | S - I | S - R | I - R |
| **% of water*** | 58.3213 | 71.3311 | 58.0237 | 0.9454 | 2.3463 | 1.7146 | 0.0238 | 0.8413 | 0.0238 |
| **starch** | 63.805 | 27.3404 | 35.5576 | 18.7104 | 4.8081 | 8.6092 | 0.0238 | 0.0238 | 0.0635 |
| **proteins*** | 21.584 | 34.0568 | 21.8057 | 5.052 | 4.2899 | 2.653 | 0.0476 | 0.8413 | 0.0476 |
| **sucrose** | 1.3803 | 1.6022 | 1.0549 | 0.4092 | 0.5587 | 0.526 | 0.4127 | 0.3333 | 0.3333 |
| **glucose** | 0.9099 | 1.2063 | 1.1396 | 0.4527 | 0.4773 | 0.4207 | 1 | 1 | 1 |
| **fructose** | 1.3366 | 1.9901 | 0.9198 | 0.0931 | 0.195 | 0.2514 | 0.0238 | 0.0278 | 0.0238 |
| *Amino acids* |  |  |  |  |  |  |  |  |  |
| **Ala*** | 0.381 | 1.7684 | 0.3101 | 0.082 | 0.4358 | 0.0466 | 0.0238 | 0.2222 | 0.0238 |
| **Arg** | 2.9417 | 4.7921 | 4.466 | 0.4383 | 2.2459 | 0.7827 | 0.0952 | 0.0238 | 0.9048 |
| **Asn** | 1.0286 | 8.5159 | 4.4021 | 0.2053 | 2.1723 | 0.9062 | 0.0159 | 0.0159 | 0.0159 |
| **Asp*** | 1.7649 | 3.4035 | 2.0518 | 0.2694 | 1.2533 | 0.3219 | 0.0476 | 0.5476 | 0.0952 |
| **GABA*** | 0.9197 | 2.8528 | 0.9451 | 0.1269 | 0.489 | 0.0915 | 0.0238 | 0.6905 | 0.0238 |
| **Gln** | 7.2421 | 24.7456 | 9.7332 | 1.3562 | 8.6007 | 0.7166 | 0.0159 | 0.0159 | 0.0159 |
| **Glu** | 3.0036 | 3.5333 | 2.7208 | 0.5339 | 1.4548 | 0.5489 | 1 | 0.8214 | 0.8214 |
| **Gly** | 0.041 | 0.2037 | 0.0442 | 0.039 | 0.2081 | 0.0267 | 0.0952 | 0.5476 | 0.0952 |
| **His** | 0.7593 | 0.5038 | 0.6071 | 0.1577 | 0.9302 | 0.3468 | 0.3095 | 0.3095 | 0.3095 |
| **Ile** | 0.3009 | 1.671 | 0.3982 | 0.0595 | 0.5413 | 0.0383 | 0.0159 | 0.0159 | 0.0159 |
| **Leu** | 0.245 | 0.9433 | 0.4821 | 0.0461 | 0.2971 | 0.0782 | 0.0159 | 0.0159 | 0.0159 |
| **Lys** | 0.2055 | 0.0943 | 0.5044 | 0.0694 | 0.1388 | 0.1263 | 0.2683 | 0.0238 | 0.0292 |
| **Phe** | 0.0821 | 0.2206 | 0.1383 | 0.018 | 0.0756 | 0.02 | 0.0238 | 0.0238 | 0.0317 |
| **Pro*** | 0.8331 | 1.3334 | 0.607 | 0.1531 | 0.3091 | 0.1649 | 0.0238 | 0.0952 | 0.0238 |
| **Ser*** | 0.4054 | 1.7558 | 0.4714 | 0.089 | 0.4482 | 0.1571 | 0.0238 | 0.6905 | 0.0238 |
| **Thr** | 1.0037 | 1.4988 | 1.6586 | 0.1453 | 0.2969 | 0.2479 | 0.0476 | 0.0238 | 0.5556 |
| **Tyr** | 0.6188 | 0.4603 | 0.7813 | 0.0782 | 0.0791 | 0.0888 | 0.0317 | 0.0238 | 0.0238 |
| **Val*** | 0.4279 | 1.7985 | 0.5127 | 0.0737 | 0.5279 | 0.0642 | 0.0238 | 0.0952 | 0.0238 |
| *Phenolic acids* |  |  |  |  |  |  |  |  |  |
| **caftaric acid** | 0.0104 | 0.0004 | 0.0028 | 0.0008 | 0.0002 | 0.0015 | 0.0159 | 0.0159 | 0.0159 |
| **caffeic acid** | 0.0004 | 0.0001 | 0.0003 | 0.0001 | 0 | 0.0002 | 0.0476 | 0.5556 | 0.5556 |
| **gallic acid** | 0.0482 | 0.0264 | 0.0228 | 0.0055 | 0.0044 | 0.0037 | 0.0238 | 0.0238 | 0.5556 |
| **isoferulic acid** | 0.0002 | 0.0008 | 0.0004 | 0.0001 | 0.0002 | 0.0002 | 0.0238 | 0.0238 | 0.0317 |
| *Flavonols* |  |  |  |  |  |  |  |  |  |
| **quercetin 3 glucoside** | 0.0013 | 0.0011 | 0.0005 | 0.0004 | 0 | 0.0001 | 0.7302 | 0.0238 | 0.0238 |
| **quercetin 3 glucuronide** | 0.0008 | 0.0003 | 0.0002 | 0.0002 | 0 | 0.0001 | 0.0238 | 0.0238 | 1 |
| **kaempferol 3 glucoside** | 0.0003 | 0.0001 | 0.0001 | 0.0002 | 0 | 0.0001 | 0.0952 | 0.0952 | 0.4127 |
| *Flavanols* |  |  |  |  |  |  |  |  |  |
| **catechin** | 0.2266 | 0.0447 | 0.1459 | 0.0165 | 0.0095 | 0.0618 | 0.0238 | 0.0238 | 0.1111 |
| **epicatechin** | 0.0813 | 0.0059 | 0.02 | 0.0126 | 0.001 | 0.008 | 0.0238 | 0.0238 | 0.0635 |
| **epicatechin gallate** | 0.0702 | 0.0126 | 0.0213 | 0.0108 | 0.002 | 0.0061 | 0.0238 | 0.0238 | 0.1111 |
| **epigallocatechin** | 0.0181 | 0.0009 | 0.0014 | 0.0036 | 0.0002 | 0.0005 | 0.0238 | 0.0238 | 0.1905 |
| **epigallocatechin gallate** | 0.0206 | 0.002 | 0.0049 | 0.0047 | 0.0009 | 0.0021 | 0.0238 | 0.0238 | 0.1111 |
| **gallocatechin** | 0.0183 | 0.0009 | 0.0015 | 0.0027 | 0.0004 | 0.0007 | 0.0238 | 0.0238 | 0.1905 |
| **B1** | 0.1839 | 0.0264 | 0.1624 | 0.0126 | 0.0048 | 0.083 | 0.0476 | 0.6905 | 0.0952 |
| **B2** | 0.0615 | 0.0059 | 0.0191 | 0.0024 | 0.0008 | 0.0079 | 0.0238 | 0.0238 | 0.1111 |
| **B3** | 0.0602 | 0.0083 | 0.0305 | 0.0047 | 0.0012 | 0.0144 | 0.0238 | 0.0238 | 0.1111 |
| **B4*** | 0.0226 | 0.0041 | 0.0125 | 0.0063 | 0.0011 | 0.0074 | 0.0476 | 0.0952 | 0.0476 |
| **B1 gallate** | 0.1915 | 0.0474 | 0.0409 | 0.0201 | 0.0056 | 0.0098 | 0.0238 | 0.0238 | 0.4127 |
| **C1** | 0.0832 | 0.0105 | 0.0287 | 0.0138 | 0.0008 | 0.0116 | 0.0238 | 0.0238 | 0.1905 |
| **trimer1*** | 0.1106 | 0.036 | 0.116 | 0.0123 | 0.0072 | 0.0464 | 0.0476 | 0.4206 | 0.0476 |
| *Stilbenes* |  |  |  |  |  |  |  |  |  |
| ***trans*-resveratrol** | 0.0214 | 0.0178 | 0.0186 | 0.0032 | 0.0062 | 0.0028 | 0.619 | 0.619 | 0.9048 |
| ***trans*-piceid** | 0.0164 | 0.0063 | 0.0176 | 0.0011 | 0.001 | 0.0077 | 0.0476 | 0.2222 | 0.1667 |
| ***cis*-piceid** | 0.0005 | 0.0013 | 0.0008 | 0.0001 | 0.0005 | 0.0003 | 0.0476 | 0.0476 | 0.1111 |
| ***trans*-piceatannol** | 0.0112 | 0.0136 | 0.0152 | 0.001 | 0.005 | 0.0041 | 0.5556 | 0.0952 | 0.5556 |
| ***trans*-astringin** | 0.0017 | 0.0013 | 0.0023 | 0.0002 | 0.0002 | 0.0009 | 0.0952 | 0.1905 | 0.1905 |
| ***cis*-astringin** | 0.0201 | 0.0023 | 0.0094 | 0.0012 | 0.0009 | 0.004 | 0.0159 | 0.0159 | 0.0159 |
| ***trans-isorhapotin**** | 0.0003 | 0.0004 | 0.0004 | 0.0001 | 0.0001 | 0.0001 | 0.9048 | 0.9048 | 0.9048 |
| ***trans*-ε-viniferin** | 1.2715 | 0.8704 | 1.0747 | 0.0885 | 0.0474 | 0.1616 | 0.0238 | 0.0238 | 0.1905 |
| ***trans-*ω-viniferin** | 0.0505 | 0.034 | 0.0464 | 0.0022 | 0.0024 | 0.0076 | 0.0476 | 0.4206 | 0.0952 |
| ***cis*-ε-viniferin** | 0.0413 | 0.0166 | 0.0266 | 0.0069 | 0.0071 | 0.0103 | 0.0476 | 0.0833 | 0.1111 |
| ***trans-*δ*-*viniferin** | 0.0199 | 0.0139 | 0.0183 | 0.0036 | 0.001 | 0.0042 | 0.1905 | 0.4206 | 0.2857 |
| **pallidol** | 0.0222 | 0.0519 | 0.0477 | 0.0021 | 0.0076 | 0.0054 | 0.0238 | 0.0238 | 0.2857 |
| **parthenocisin A** | 0.0068 | 0.0205 | 0.0115 | 0.0013 | 0.0038 | 0.0042 | 0.0238 | 0.0238 | 0.0635 |
| **ampelopsin A** | 0.1292 | 0.0286 | 0.0196 | 0.0235 | 0.0059 | 0.0091 | 0.0238 | 0.0238 | 0.1111 |
| **vitisinol C** | 0.0104 | 0.0021 | 0.0026 | 0.0011 | 0.0005 | 0.0005 | 0.0238 | 0.0238 | 0.2857 |
| **dimer diglycoside** | 0.0032 | 0.0027 | 0.0033 | 0.0001 | 0.0003 | 0.0004 | 0.0952 | 1 | 0.0952 |
| **dimer glycoside A** | 0.002 | 0.0016 | 0.0022 | 0.0003 | 0.0002 | 0.0002 | 0.0952 | 0.4206 | 0.0476 |
| **dimer glycoside B** | 0.0108 | 0.0023 | 0.0019 | 0.0015 | 0.0002 | 0.0005 | 0.0238 | 0.0238 | 0.2857 |
| **dimer glycoside C** | 0.0048 | 0.0021 | 0.0044 | 0.0005 | 0.0003 | 0.0014 | 0.0476 | 1 | 0.0952 |
| **α-viniferin** | 0.1067 | 0.2142 | 0.0997 | 0.0082 | 0.0513 | 0.0512 | 0.0476 | 0.1508 | 0.0952 |
| **miyabenol C** | 0.2264 | 0.0971 | 0.0402 | 0.0209 | 0.0088 | 0.032 | 0.0238 | 0.0238 | 0.0635 |
| **hopeaphenol** | 0.1378 | 0.1484 | 0.132 | 0.0125 | 0.0293 | 0.0425 | 0.8413 | 0.8413 | 0.8413 |
| **isohopeaphenol** | 0.0613 | 0.2691 | 0.3008 | 0.0105 | 0.0528 | 0.0679 | 0.0238 | 0.0238 | 0.4127 |
| **r2-viniferin** | 0.0039 | 0.0092 | 0.025 | 0.001 | 0.003 | 0.0106 | 0.0238 | 0.0238 | 0.1905 |
| **r-viniferin** | 0.0185 | 0.0874 | 0.1803 | 0.0113 | 0.0267 | 0.0734 | 0.0238 | 0.0238 | 0.1905 |
| *Others* |  |  |  |  |  |  |  |  |  |
| **naringenin** | 0.0002 | 0.0008 | 0.0005 | 0 | 0.0001 | 0.0002 | 0.0238 | 0.0238 | 0.1111 |
| **naringenin glucoside** | 0.2259 | 0.0121 | 0.0112 | 0.0433 | 0.0044 | 0.0043 | 0.0238 | 0.0238 | 0.9048 |
| **phloretin** | 0.0006 | 0.0001 | 0.0009 | 0.0001 | 0.0001 | 0.0005 | 0.0476 | 0.1508 | 0.0952 |
| **taxifolin** | 0.0002 | 0.0014 | 0.0006 | 0 | 0.0001 | 0.0004 | 0.0159 | 0.0159 | 0.0159 |

Supplementary table 9: Metabolite concentration, protein content and percentage of water in the scion wood, graft interface and rootstock wood of hetero-grafts of Vitis vinifera cv. Négrette onto Vitis berlandieri x Vitis riparia cv. Sélection Oppenheim 4, 33 days after grafting. Mean concentrations and standard deviation shown (n = 5). p-value of Wilcoxon-test between scion and interface tissues (S – I), scion and rootstock tissues (S – R), and interface and rootstock tissues (I -R). Stars indicates a significant difference between S – I and between I – R, but no significant difference between S - R. Significance threshold set at p-value < 0.05.

| **Variables** | **Mean** | | | **standard deviation** | | | ***p-value* wilcoxon-test** | | |
| --- | --- | --- | --- | --- | --- | --- | --- | --- | --- |
|  | Scion | Interface | Rootstock | Scion | Interface | Rootstock | S - I | S - R | I - R |
| **% of water*** | 56.7262 | 69.3931 | 55.2616 | 1.9615 | 1.3373 | 1.0841 | 0.0119 | 0.4206 | 0.0119 |
| **starch** | 92.3994 | 48.3979 | 60.6782 | 6.8278 | 5.6848 | 8.937 | 0.0119 | 0.0119 | 0.0317 |
| **proteins** | 19.3283 | 30.565 | 34.5766 | 3.0303 | 8.3873 | 7.4356 | 0.1429 | 0.0238 | 0.8413 |
| **sucrose** | 1.5619 | 1.675 | 1.0506 | 0.7114 | 0.455 | 0.7281 | 0.6905 | 0.4643 | 0.4643 |
| **glucose** | 1.5132 | 2.1158 | 1.6203 | 0.5013 | 0.3617 | 0.2377 | 0.1429 | 1 | 0.0952 |
| **fructose** | 0.9994 | 3.171 | 1.4736 | 0.2833 | 0.6306 | 0.2203 | 0.0119 | 0.0159 | 0.0119 |
| *Amino acids* |  |  |  |  |  |  |  |  |  |
| **Ala*** | 0.5128 | 0.974 | 0.328 | 0.2791 | 0.2453 | 0.0939 | 0.0476 | 0.4206 | 0.0238 |
| **Arg** | 4.1938 | 5.9163 | 4.8178 | 1.2114 | 0.4818 | 1.7136 | 0.0238 | 0.6905 | 0.631 |
| **Asn** | 1.9959 | 19.3726 | 7.3341 | 0.601 | 3.3045 | 1.864 | 0.0079 | 0.0079 | 0.0079 |
| **Asp*** | 1.9729 | 3.1489 | 2.0932 | 0.6185 | 0.3964 | 0.5467 | 0.0238 | 0.8413 | 0.0238 |
| **GABA*** | 0.7251 | 1.8169 | 0.8643 | 0.1611 | 0.3289 | 0.2935 | 0.0119 | 0.5476 | 0.0119 |
| **Gln*** | 7.3154 | 28.5064 | 10.1371 | 2.3163 | 5.5926 | 2.1769 | 0.0119 | 0.0952 | 0.0119 |
| **Glu** | 4.8132 | 3.7301 | 2.8611 | 4.1132 | 0.6359 | 0.7864 | 1 | 0.8214 | 0.2857 |
| **Gly** | 0.0984 | 0.0671 | 0.0308 | 0.1597 | 0.0548 | 0.0254 | 0.6905 | 0.6905 | 0.6905 |
| **His** | 0.8108 | 0.751 | 0.5117 | 0.2453 | 0.6618 | 0.493 | 0.8413 | 0.8413 | 0.8413 |
| **Ile** | 0.2712 | 1.665 | 0.4795 | 0.0968 | 0.3749 | 0.0996 | 0.0119 | 0.0317 | 0.0119 |
| **Leu** | 0.2144 | 0.8718 | 0.4659 | 0.0799 | 0.1034 | 0.0918 | 0.0079 | 0.0079 | 0.0079 |
| **Lys** | 0.2481 | 0.1392 | 0.3641 | 0.0545 | 0.2733 | 0.1163 | 0.1508 | 0.1508 | 0.1508 |
| **Phe*** | 0.0789 | 0.1726 | 0.0995 | 0.0434 | 0.0248 | 0.0136 | 0.0238 | 0.4206 | 0.0238 |
| **Pro*** | 0.7127 | 1.3056 | 0.7768 | 0.1611 | 0.2827 | 0.165 | 0.0238 | 0.6905 | 0.0238 |
| **Ser*** | 0.4765 | 1.6419 | 0.5099 | 0.3014 | 0.2785 | 0.1664 | 0.0119 | 0.4206 | 0.0119 |
| **Thr*** | 0.951 | 1.9372 | 1.2426 | 0.2302 | 0.1693 | 0.2479 | 0.0119 | 0.0952 | 0.0119 |
| **Tyr** | 0.6397 | 0.4944 | 0.8121 | 0.1719 | 0.0261 | 0.0862 | 0.1508 | 0.1508 | 0.0238 |
| **Val*** | 0.3482 | 1.3475 | 0.4991 | 0.1159 | 0.2898 | 0.1067 | 0.0119 | 0.0556 | 0.0119 |
| *Phenolic acids* |  |  |  |  |  |  |  |  |  |
| **caftaric acid*** | 0.0062 | 0.0005 | 0.0047 | 0.0009 | 0.0003 | 0.0015 | 0.0119 | 0.2222 | 0.0119 |
| **caffeic acid** | 0.0003 | 0.0002 | 0.0004 | 0.0001 | 0.0001 | 0.0003 | 0.2857 | 1 | 0.3333 |
| **gallic acid** | 0.0411 | 0.025 | 0.0254 | 0.0068 | 0.0041 | 0.0052 | 0.0119 | 0.0119 | 0.6905 |
| **isoferulic acid*** | 0.0003 | 0.0007 | 0.0003 | 0.0001 | 0.0001 | 0.0001 | 0.0119 | 0.6905 | 0.0119 |
| *Flavonols* |  |  |  |  |  |  |  |  |  |
| **quercetin 3 glucoside** | 0.004 | 0.0012 | 0.0023 | 0.0066 | 0.0005 | 0.0019 | 0.8413 | 0.8413 | 0.8413 |
| **quercetin 3 glucuronide** | 0.0005 | 0.001 | 0.0036 | 0.0001 | 0.0001 | 0.0024 | 0.0079 | 0.0079 | 0.0079 |
| **kaempferol 3 glucoside** | 0.0001 | 0.0001 | 0.0003 | 0.0001 | 0 | 0.0002 | 0.6905 | 0.4524 | 0.4643 |
| *Flavanols* |  |  |  |  |  |  |  |  |  |
| **catechin** | 0.1582 | 0.0545 | 0.3225 | 0.041 | 0.0128 | 0.0518 | 0.0079 | 0.0079 | 0.0079 |
| **epicatechin*** | 0.0613 | 0.0079 | 0.0512 | 0.0367 | 0.003 | 0.0195 | 0.0119 | 1 | 0.0119 |
| **epicatechin gallate*** | 0.0692 | 0.022 | 0.0487 | 0.0157 | 0.0052 | 0.0102 | 0.0119 | 0.0556 | 0.0119 |
| **epigallocatechin** | 0.0175 | 0.0009 | 0.0018 | 0.0178 | 0.0005 | 0.0008 | 0.0119 | 0.0119 | 0.2222 |
| **epigallocatechin gallate** | 0.02 | 0.0024 | 0.0068 | 0.0035 | 0.0012 | 0.0019 | 0.0079 | 0.0079 | 0.0079 |
| **gallocatechin** | 0.0187 | 0.0013 | 0.0019 | 0.0182 | 0.0008 | 0.0011 | 0.0119 | 0.0119 | 0.4206 |
| **B1*** | 0.1313 | 0.0246 | 0.1673 | 0.0265 | 0.0082 | 0.0197 | 0.0119 | 0.0952 | 0.0119 |
| **B2** | 0.0399 | 0.005 | 0.0248 | 0.0076 | 0.0015 | 0.0025 | 0.0079 | 0.0079 | 0.0079 |
| **B3*** | 0.0439 | 0.0096 | 0.0526 | 0.0096 | 0.0023 | 0.0106 | 0.0119 | 0.3095 | 0.0119 |
| **B4*** | 0.0195 | 0.0031 | 0.018 | 0.0021 | 0.0012 | 0.0058 | 0.0119 | 0.4206 | 0.0119 |
| **B1 gallate** | 0.1761 | 0.0527 | 0.0514 | 0.0449 | 0.0098 | 0.0104 | 0.0119 | 0.0119 | 1 |
| **C1** | 0.0493 | 0.0088 | 0.0287 | 0.0114 | 0.0039 | 0.0032 | 0.0079 | 0.0079 | 0.0079 |
| **trimer1*** | 0.0717 | 0.0294 | 0.0865 | 0.011 | 0.0098 | 0.0131 | 0.0119 | 0.0952 | 0.0119 |
| *Stilbenes* |  |  |  |  |  |  |  |  |  |
| ***trans*-resveratrol** | 0.0395 | 0.0387 | 0.0495 | 0.0127 | 0.0137 | 0.0167 | 0.8413 | 0.631 | 0.631 |
| ***trans*-piceid*** | 0.0172 | 0.0097 | 0.0202 | 0.0038 | 0.0031 | 0.0058 | 0.0238 | 0.5476 | 0.0238 |
| ***cis*-piceid*** | 0.0005 | 0.0016 | 0.0005 | 0 | 0.0006 | 0.0001 | 0.0119 | 0.8413 | 0.0119 |
| ***trans*-piceatannol** | 0.0295 | 0.0256 | 0.0367 | 0.0067 | 0.0077 | 0.0092 | 0.4206 | 0.4206 | 0.2857 |
| ***trans*-astringin** | 0.0026 | 0.0016 | 0.0027 | 0.0006 | 0.0006 | 0.001 | 0.0833 | 0.8413 | 0.0833 |
| ***cis*-astringin** | 0.0229 | 0.0036 | 0.0114 | 0.0036 | 0.0009 | 0.002 | 0.0079 | 0.0079 | 0.0079 |
| ***trans-isorhapotin**** | 0.0006 | 0.0003 | 0.0004 | 0.0001 | 0.0002 | 0.0001 | 0.0833 | 0.0833 | 0.5476 |
| ***trans*-ε-viniferin** | 1.1167 | 0.8762 | 1.0505 | 0.1051 | 0.1071 | 0.2019 | 0.0476 | 0.5476 | 0.2262 |
| ***trans-*ω-viniferin** | 0.0471 | 0.0425 | 0.0405 | 0.0023 | 0.0034 | 0.0061 | 0.0952 | 0.1429 | 0.4206 |
| ***cis*-ε-viniferin** | 0.0247 | 0.0283 | 0.021 | 0.0075 | 0.0107 | 0.0048 | 0.8413 | 0.4643 | 0.4643 |
| ***trans-*δ*-*viniferin*** | 0.0214 | 0.0138 | 0.0182 | 0.0035 | 0.0022 | 0.0027 | 0.0238 | 0.3095 | 0.0476 |
| **pallidol** | 0.0268 | 0.0458 | 0.0351 | 0.0091 | 0.0109 | 0.0094 | 0.0952 | 0.1429 | 0.1508 |
| **parthenocisin A*** | 0.01 | 0.0181 | 0.0082 | 0.003 | 0.0027 | 0.0027 | 0.0119 | 0.3095 | 0.0119 |
| **ampelopsin A** | 0.1058 | 0.0256 | 0.0063 | 0.0092 | 0.0054 | 0.0013 | 0.0079 | 0.0079 | 0.0079 |
| **vitisinol C** | 0.0075 | 0.0025 | 0.0033 | 0.0007 | 0.0005 | 0.0009 | 0.0119 | 0.0119 | 0.0952 |
| **dimer diglycoside** | 0.0037 | 0.0038 | 0.0058 | 0.0008 | 0.0004 | 0.0011 | 0.6905 | 0.0119 | 0.0119 |
| **dimer glycoside A** | 0.0021 | 0.002 | 0.003 | 0.0004 | 0.0003 | 0.0005 | 0.5476 | 0.0119 | 0.0119 |
| **dimer glycoside B** | 0.0097 | 0.0032 | 0.0056 | 0.0006 | 0.0005 | 0.0007 | 0.0079 | 0.0079 | 0.0079 |
| **dimer glycoside C** | 0.0044 | 0.0032 | 0.0072 | 0.0005 | 0.0006 | 0.0017 | 0.0159 | 0.0119 | 0.0119 |
| **α-viniferin*** | 0.0704 | 0.2134 | 0.0846 | 0.0182 | 0.0319 | 0.0196 | 0.0119 | 0.3095 | 0.0119 |
| **miyabenol C** | 0.2236 | 0.1616 | 0.0412 | 0.0335 | 0.038 | 0.0132 | 0.0952 | 0.0119 | 0.0119 |
| **hopeaphenol** | 0.171 | 0.2721 | 0.4006 | 0.0391 | 0.0781 | 0.1083 | 0.0476 | 0.0238 | 0.0952 |
| **isohopeaphenol** | 0.0836 | 0.3496 | 0.4869 | 0.0233 | 0.0923 | 0.1261 | 0.0119 | 0.0119 | 0.0952 |
| **r2-viniferin** | 0.004 | 0.0035 | 0.0097 | 0.0007 | 0.0008 | 0.0029 | 0.5476 | 0.0119 | 0.0119 |
| **r-viniferin** | 0.0385 | 0.0448 | 0.0884 | 0.0136 | 0.0072 | 0.0227 | 0.4206 | 0.0119 | 0.0119 |
| *Others* |  |  |  |  |  |  |  |  |  |
| **naringenin** | 0.0001 | 0.0009 | 0.0005 | 0 | 0.0001 | 0.0001 | 0.0079 | 0.0079 | 0.0079 |
| **naringenin glucoside** | 0.1677 | 0.0173 | 0.0279 | 0.0255 | 0.0046 | 0.0103 | 0.0119 | 0.0119 | 0.0556 |
| **phloretin** | 0.0004 | 0.0003 | 0.0019 | 0 | 0.0001 | 0.0004 | 0.1508 | 0.0119 | 0.0119 |
| **taxifolin** | 0.0001 | 0.0013 | 0.0008 | 0 | 0.0003 | 0.0001 | 0.0079 | 0.0079 | 0.0079 |

Supplementary table 10: Metabolite concentration, protein content and percentage of water in the scion wood, graft interface and rootstock wood of homo-grafts of Vitis berlandieri x Vitis riparia cv. Rességuier Sélection Birolleau 1, 33 days after grafting. Mean concentrations and standard deviation shown (n = 5). p-value of Wilcoxon-test between scion and interface tissues (S – I), scion and rootstock tissues (S – R), and interface and rootstock tissues (I -R). Stars indicates a significant difference between S – I and between I – R, but no significant difference between S - R. Significance threshold set at p-value < 0.05.

| **Variables** | **Mean** | | | **standard deviation** | | | ***p-value* wilcoxon-test** | | |
| --- | --- | --- | --- | --- | --- | --- | --- | --- | --- |
|  | Scion | Interface | Rootstock | Scion | Interface | Rootstock | S - I | S - R | I - R |
| **% of water*** | 61.5183 | 77.592 | 59.7027 | 1.8669 | 1.6978 | 0.7853 | 0.0119 | 0.0556 | 0.0119 |
| **starch** | 23.3961 | 13.426 | 39.479 | 15.6019 | 5.2658 | 8.2591 | 0.3095 | 0.1429 | 0.0238 |
| **proteins*** | 21.1391 | 28.3942 | 22.2718 | 2.6902 | 4.4298 | 2.1074 | 0.0238 | 1 | 0.0476 |
| **sucrose*** | 0.3822 | 1.597 | 0.634 | 0.1836 | 0.524 | 0.2956 | 0.0238 | 0.2222 | 0.0476 |
| **glucose** | 0.7343 | 1.3806 | 0.9191 | 0.442 | 0.2828 | 0.6615 | 0.0952 | 1 | 0.3333 |
| **fructose*** | 0.49 | 2.0207 | 0.7892 | 0.3938 | 0.3963 | 0.4313 | 0.0119 | 0.2222 | 0.0119 |
| *Amino acids* |  |  |  |  |  |  |  |  |  |
| **Ala*** | 0.316 | 1.0838 | 0.4271 | 0.1107 | 0.3138 | 0.1466 | 0.0119 | 0.2222 | 0.0119 |
| **Arg** | 5.7033 | 3.9693 | 5.1047 | 0.9925 | 1.0709 | 1.425 | 0.1667 | 0.6905 | 0.3333 |
| **Asn** | 4.0726 | 6.2463 | 4.6761 | 0.7597 | 1.3361 | 0.6537 | 0.0952 | 0.3095 | 0.1429 |
| **Asp** | 2.2213 | 2.7739 | 2.5665 | 0.1902 | 0.3831 | 0.5231 | 0.0952 | 0.8413 | 0.8413 |
| **GABA** | 1.1399 | 1.6291 | 1.3032 | 0.2308 | 0.4876 | 0.3228 | 0.2857 | 0.4206 | 0.4206 |
| **Gln*** | 9.8625 | 20.3702 | 12.8325 | 0.7004 | 3.7377 | 3.1008 | 0.0238 | 0.0556 | 0.0238 |
| **Glu** | 2.8997 | 2.8629 | 3.2345 | 0.2585 | 0.5053 | 0.6204 | 0.8413 | 0.8214 | 0.8214 |
| **Gly** | 0.1472 | 0.2497 | 0.0608 | 0.228 | 0.2736 | 0.03 | 0.3333 | 0.8413 | 0.0952 |
| **His** | 0.9206 | 0.6357 | 1.032 | 0.4687 | 0.3767 | 0.2206 | 0.3333 | 1 | 0.2857 |
| **Ile*** | 0.4627 | 1.5787 | 0.5849 | 0.0776 | 0.3964 | 0.1856 | 0.0119 | 0.3095 | 0.0119 |
| **Leu*** | 0.5864 | 0.951 | 0.6767 | 0.1061 | 0.2482 | 0.1369 | 0.0238 | 0.3095 | 0.0476 |
| **Lys** | 0.6184 | 0.2779 | 0.5657 | 0.1421 | 0.291 | 0.1784 | 0.2262 | 0.5476 | 0.2262 |
| **Phe** | 0.1312 | 0.1932 | 0.176 | 0.0246 | 0.0419 | 0.0293 | 0.0952 | 0.1429 | 0.5476 |
| **Pro** | 0.521 | 0.8437 | 0.7428 | 0.0541 | 0.2512 | 0.154 | 0.0476 | 0.0476 | 0.6905 |
| **Ser*** | 0.6003 | 1.3613 | 0.5218 | 0.3857 | 0.4735 | 0.1949 | 0.0476 | 1 | 0.0238 |
| **Thr*** | 1.6726 | 1.5035 | 1.3783 | 0.4621 | 0.165 | 0.2965 | 0.5476 | 0.5476 | 0.5476 |
| **Tyr*** | 1.0648 | 0.4834 | 0.9599 | 0.0937 | 0.0587 | 0.2127 | 0.0119 | 0.3095 | 0.0119 |
| **Val*** | 0.4682 | 1.5125 | 0.6711 | 0.0666 | 0.3001 | 0.2021 | 0.0119 | 0.0556 | 0.0119 |
| *Phenolic acids* |  |  |  |  |  |  |  |  |  |
| **caftaric acid*** | 0.0027 | 0.0005 | 0.0049 | 0.0008 | 0.0003 | 0.0021 | 0.0119 | 0.1508 | 0.0119 |
| **caffeic acid** | 0.0001 | 0.0001 | 0.0002 | 0.0001 | 0 | 0.0001 | 0.2222 | 0.2222 | 0.0238 |
| **gallic acid** | 0.017 | 0.0202 | 0.0255 | 0.0041 | 0.0035 | 0.0044 | 0.1508 | 0.1429 | 0.1429 |
| **isoferulic acid*** | 0.0004 | 0.0008 | 0.0005 | 0.0002 | 0.0001 | 0.0001 | 0.0119 | 0.5476 | 0.0119 |
| *Flavonols* |  |  |  |  |  |  |  |  |  |
| **quercetin 3 glucoside** | 0.0003 | 0.0005 | 0.0003 | 0.0002 | 0.0002 | 0.0001 | 0.2262 | 0.6905 | 0.2262 |
| **quercetin 3 glucuronide** | 0.0002 | 0.0001 | 0.0004 | 0.0002 | 0 | 0.0003 | 0.8413 | 0.8413 | 0.0952 |
| **kaempferol 3 glucoside** | 0 | 0 | 0 | 0 | 0 | 0 | 0.8413 | 0.8413 | 0.2857 |
| *Flavanols* |  |  |  |  |  |  |  |  |  |
| **catechin*** | 0.1619 | 0.031 | 0.1739 | 0.0395 | 0.0092 | 0.045 | 0.0119 | 0.6905 | 0.0119 |
| **epicatechin*** | 0.0229 | 0.004 | 0.0236 | 0.0085 | 0.0014 | 0.0082 | 0.0119 | 0.8413 | 0.0119 |
| **epicatechin gallate*** | 0.0259 | 0.0073 | 0.0258 | 0.0176 | 0.0026 | 0.0055 | 0.0119 | 0.2222 | 0.0119 |
| **epigallocatechin** | 0.0014 | 0.0002 | 0.0012 | 0.0009 | 0.0002 | 0.0003 | 0.0833 | 1 | 0.0238 |
| **epigallocatechin gallate*** | 0.0058 | 0.0009 | 0.0065 | 0.0046 | 0.0004 | 0.0013 | 0.0119 | 0.2222 | 0.0119 |
| **gallocatechin*** | 0.0016 | 0.0003 | 0.0013 | 0.0012 | 0.0002 | 0.0006 | 0.0238 | 0.8413 | 0.0238 |
| **B1*** | 0.1567 | 0.023 | 0.1805 | 0.0178 | 0.0023 | 0.0332 | 0.0119 | 0.3095 | 0.0119 |
| **B2*** | 0.024 | 0.0028 | 0.0235 | 0.0045 | 0.0006 | 0.003 | 0.0119 | 1 | 0.0119 |
| **B3*** | 0.0273 | 0.0091 | 0.0332 | 0.0089 | 0.0069 | 0.0071 | 0.0238 | 0.2222 | 0.0238 |
| **B4*** | 0.0142 | 0.003 | 0.0139 | 0.0054 | 0.0007 | 0.0076 | 0.0119 | 1 | 0.0119 |
| **B1 gallate** | 0.0402 | 0.0247 | 0.0416 | 0.0298 | 0.0025 | 0.0073 | 0.4206 | 0.2262 | 0.0238 |
| **C1*** | 0.0331 | 0.0111 | 0.0338 | 0.0076 | 0.0052 | 0.0039 | 0.0119 | 1 | 0.0119 |
| **trimer1*** | 0.1102 | 0.0349 | 0.1373 | 0.0093 | 0.0031 | 0.0291 | 0.0119 | 0.0556 | 0.0119 |
| *Stilbenes* |  |  |  |  |  |  |  |  |  |
| ***trans*-resveratrol** | 0.0164 | 0.0102 | 0.0208 | 0.0073 | 0.0049 | 0.0028 | 0.2222 | 0.2222 | 0.0238 |
| ***trans*-piceid*** | 0.0181 | 0.0051 | 0.0219 | 0.0083 | 0.0016 | 0.0037 | 0.0119 | 0.3095 | 0.0119 |
| ***cis*-piceid** | 0.0004 | 0.0009 | 0.0005 | 0.0001 | 0.0004 | 0.0002 | 0.0952 | 0.4206 | 0.1429 |
| ***trans*-piceatannol*** | 0.0157 | 0.0083 | 0.0174 | 0.0061 | 0.0028 | 0.0039 | 0.0238 | 0.4206 | 0.0238 |
| ***trans*-astringin*** | 0.0035 | 0.0012 | 0.0032 | 0.0014 | 0.0003 | 0.0006 | 0.0119 | 0.6905 | 0.0119 |
| ***cis*-astringin*** | 0.0131 | 0.0027 | 0.011 | 0.0049 | 0.0013 | 0.0027 | 0.0119 | 0.4206 | 0.0119 |
| ***trans-isorhapotin**** | 0.0004 | 0.0003 | 0.0004 | 0.0002 | 0.0001 | 0.0001 | 0.4643 | 0.5476 | 0.1667 |
| ***trans*-ε-viniferin*** | 0.9631 | 0.639 | 1.1641 | 0.1197 | 0.1092 | 0.181 | 0.0119 | 0.1508 | 0.0119 |
| ***trans-*ω-viniferin*** | 0.0411 | 0.0238 | 0.0517 | 0.0065 | 0.0068 | 0.0065 | 0.0238 | 0.0556 | 0.0238 |
| ***cis*-ε-viniferin** | 0.0251 | 0.0175 | 0.0187 | 0.0105 | 0.0078 | 0.0049 | 0.5476 | 0.5476 | 0.5476 |
| ***trans-*δ*-*viniferin*** | 0.0238 | 0.0131 | 0.0217 | 0.0029 | 0.0019 | 0.0039 | 0.0238 | 0.6905 | 0.0238 |
| **pallidol** | 0.0397 | 0.042 | 0.0509 | 0.0048 | 0.0053 | 0.0137 | 0.5476 | 0.4643 | 0.4643 |
| **parthenocisin A** | 0.0088 | 0.0125 | 0.0101 | 0.0011 | 0.0031 | 0.0036 | 0.1667 | 0.6905 | 0.3333 |
| **ampelopsin A*** | 0.0211 | 0.0059 | 0.0137 | 0.0101 | 0.002 | 0.0086 | 0.0238 | 0.2222 | 0.0238 |
| **vitisinol C*** | 0.0021 | 0.0005 | 0.002 | 0.0013 | 0.0002 | 0.0003 | 0.0119 | 0.5476 | 0.0119 |
| **dimer diglycoside** | 0.004 | 0.0032 | 0.0048 | 0.0012 | 0.0008 | 0.0019 | 0.631 | 0.6905 | 0.2857 |
| **dimer glycoside A** | 0.0024 | 0.0019 | 0.0031 | 0.0007 | 0.0005 | 0.0013 | 0.3333 | 0.8413 | 0.0952 |
| **dimer glycoside B** | 0.0029 | 0.0006 | 0.002 | 0.0034 | 0.0005 | 0.0008 | 0.0833 | 0.8413 | 0.0476 |
| **dimer glycoside C*** | 0.0041 | 0.0016 | 0.0055 | 0.0008 | 0.0006 | 0.0007 | 0.0119 | 0.0556 | 0.0119 |
| **α-viniferin** | 0.1363 | 0.1505 | 0.0875 | 0.0321 | 0.0308 | 0.0244 | 0.6905 | 0.0476 | 0.0476 |
| **miyabenol C** | 0.0262 | 0.0376 | 0.0265 | 0.0126 | 0.015 | 0.0101 | 0.2857 | 0.8413 | 0.3333 |
| **hopeaphenol** | 0.0696 | 0.0806 | 0.1244 | 0.0239 | 0.0186 | 0.0465 | 0.4206 | 0.0952 | 0.2262 |
| **isohopeaphenol** | 0.22 | 0.1983 | 0.3125 | 0.042 | 0.0329 | 0.1097 | 1 | 0.1429 | 0.1429 |
| **r2-viniferin*** | 0.0213 | 0.0077 | 0.0231 | 0.0061 | 0.0015 | 0.003 | 0.0119 | 0.5476 | 0.0119 |
| **r-viniferin*** | 0.174 | 0.0789 | 0.1804 | 0.0332 | 0.013 | 0.0259 | 0.0119 | 0.8413 | 0.0119 |
| *Others* |  |  |  |  |  |  |  |  |  |
| **naringenin*** | 0.0003 | 0.0006 | 0.0004 | 0.0001 | 0.0001 | 0.0001 | 0.0119 | 0.6905 | 0.0119 |
| **naringenin glucoside** | 0.0157 | 0.0051 | 0.0093 | 0.0084 | 0.0025 | 0.0032 | 0.0238 | 0.0952 | 0.0833 |
| **phloretin*** | 0.0008 | 0.0001 | 0.001 | 0.0003 | 0.0001 | 0.0003 | 0.0119 | 0.2222 | 0.0119 |
| **taxifolin*** | 0.0003 | 0.0011 | 0.0006 | 0.0001 | 0.0002 | 0.0002 | 0.0119 | 0.0556 | 0.0119 |

Supplementary table 11: Metabolite concentration, protein content and percentage of water in the scion wood, graft interface and rootstock wood of homo-grafts of Vitis berlandieri x Vitis riparia cv. Sélection Oppenheim 4, 33 days after grafting. Mean concentrations and standard deviation shown (n = 5). p-value of Wilcoxon-test between scion and interface tissues (S – I), scion and rootstock tissues (S – R), and interface and rootstock tissues (I -R). Stars indicates a significant difference between S – I and between I – R, but no significant difference between S - R. Significance threshold set at p-value < 0.05.

| **Variables** | **Mean** | | | **standard deviation** | | | ***p-value* wilcoxon-test** | | |
| --- | --- | --- | --- | --- | --- | --- | --- | --- | --- |
|  | Scion | Interface | Rootstock | Scion | Interface | Rootstock | S - I | S - R | I - R |
| **% of water** | 54.6931 | 73.3303 | 56.8488 | 1.1964 | 3.5094 | 0.8796 | 0.0179 | 0.0361 | 0.0179 |
| **starch** | 70.8074 | 33.9455 | 56.9988 | 16.9566 | 18.6973 | 5.1479 | 0.0952 | 0.4206 | 0.2262 |
| **proteins** | 37.2042 | 24.3037 | 34.4836 | 8.6748 | 6.4588 | 6.248 | 0.0833 | 0.5476 | 0.0833 |
| **sucrose** | 1.2696 | 1.6068 | 1.963 | 0.1922 | 1.3189 | 0.3869 | 0.8413 | 0.0952 | 0.8413 |
| **glucose** | 1.3474 | 1.4003 | 1.2765 | 0.5094 | 0.3498 | 0.8402 | 1 | 1 | 1 |
| **fructose*** | 1.2996 | 2.6335 | 1.3691 | 0.3794 | 0.5269 | 0.2824 | 0.0238 | 0.6905 | 0.0238 |
| *Amino acids* |  |  |  |  |  |  |  |  |  |
| **Ala*** | 0.4077 | 1.1723 | 0.4041 | 0.1053 | 0.4509 | 0.0915 | 0.0119 | 0.8413 | 0.0119 |
| **Arg** | 5.5902 | 5.7324 | 5.3663 | 1.7347 | 1.2782 | 0.5401 | 1 | 1 | 1 |
| **Asn*** | 8.9604 | 24.395 | 7.9541 | 1.3208 | 7.9869 | 1.9673 | 0.0238 | 0.3095 | 0.0238 |
| **Asp*** | 2.4924 | 3.7431 | 2.4746 | 0.5148 | 0.4249 | 0.336 | 0.0238 | 1 | 0.0238 |
| **GABA*** | 1.2242 | 2.5059 | 1.1773 | 0.3278 | 0.561 | 0.294 | 0.0119 | 1 | 0.0119 |
| **Gln*** | 10.9158 | 30.2629 | 13.6175 | 1.8398 | 2.7839 | 2.5492 | 0.0119 | 0.0952 | 0.0119 |
| **Glu** | 3.0946 | 3.6056 | 3.4296 | 0.8015 | 0.4482 | 0.2104 | 0.8413 | 0.8413 | 0.8413 |
| **Gly*** | 0.0042 | 0.0888 | 0.0103 | 0.0058 | 0.037 | 0.0116 | 0.0179 | 0.5038 | 0.0179 |
| **His** | 0.4505 | 0.7613 | 0.4874 | 0.1139 | 0.379 | 0.0903 | 0.2262 | 0.5476 | 0.2262 |
| **Ile** | 0.3997 | 1.3246 | 0.5717 | 0.0445 | 0.6134 | 0.157 | 0.0952 | 0.0952 | 0.0952 |
| **Leu** | 0.4268 | 0.8794 | 0.5441 | 0.0612 | 0.4262 | 0.1326 | 0.1508 | 0.1508 | 0.1508 |
| **Lys*** | 0.3525 | 0.0778 | 0.399 | 0.1277 | 0.0905 | 0.0263 | 0.0318 | 1 | 0.0318 |
| **Phe** | 0.1011 | 0.1504 | 0.1251 | 0.0242 | 0.0345 | 0.0215 | 0.1667 | 0.3095 | 0.3095 |
| **Pro** | 0.7446 | 1.3006 | 1.1099 | 0.2235 | 0.2303 | 0.2241 | 0.0238 | 0.0833 | 0.3095 |
| **Ser** | 0.5099 | 1.6295 | 0.8237 | 0.2135 | 0.3085 | 0.1999 | 0.0119 | 0.0317 | 0.0119 |
| **Thr** | 1.3248 | 1.6889 | 1.4796 | 0.1148 | 0.2789 | 0.1316 | 0.1429 | 0.1429 | 0.1508 |
| **Tyr*** | 1.1593 | 0.4802 | 0.9976 | 0.1992 | 0.1058 | 0.1208 | 0.0119 | 0.1508 | 0.0119 |
| **Val** | 0.4364 | 1.3308 | 0.7252 | 0.0834 | 0.4812 | 0.1799 | 0.0238 | 0.0476 | 0.0556 |
| *Phenolic acids* |  |  |  |  |  |  |  |  |  |
| **caftaric acid*** | 0.0023 | 0.0003 | 0.0035 | 0.0006 | 0.0002 | 0.0008 | 0.0119 | 0.0952 | 0.0119 |
| **caffeic acid** | 0.0004 | 0.0002 | 0.0003 | 0.0001 | 0.0001 | 0.0001 | 0.0476 | 0.1429 | 0.1508 |
| **gallic acid** | 0.0166 | 0.0199 | 0.0226 | 0.0013 | 0.0031 | 0.0053 | 0.0833 | 0.0833 | 0.5476 |
| **isoferulic acid** | 0.0007 | 0.0008 | 0.0004 | 0.0002 | 0.0003 | 0 | 0.5476 | 0.2262 | 0.2262 |
| *Flavonols* |  |  |  |  |  |  |  |  |  |
| **quercetin 3 glucoside** | 0.0008 | 0.0017 | 0.0013 | 0.0005 | 0.0009 | 0.0009 | 0.6667 | 0.6905 | 0.6905 |
| **quercetin 3 glucuronide** | 0.0015 | 0.0007 | 0.0031 | 0.001 | 0.0005 | 0.0023 | 0.3095 | 0.3095 | 0.0952 |
| **kaempferol 3 glucoside** | 0.0001 | 0.0001 | 0.0001 | 0.0001 | 0.0001 | 0.0001 | 0.3333 | 0.3333 | 1 |
| *Flavanols* |  |  |  |  |  |  |  |  |  |
| **catechin*** | 0.2931 | 0.0389 | 0.3037 | 0.0308 | 0.0116 | 0.079 | 0.0119 | 0.8413 | 0.0119 |
| **epicatechin*** | 0.0453 | 0.005 | 0.0338 | 0.0064 | 0.0022 | 0.0085 | 0.0119 | 0.0556 | 0.0119 |
| **epicatechin gallate** | 0.0532 | 0.0126 | 0.042 | 0.0066 | 0.0069 | 0.0067 | 0.0119 | 0.0317 | 0.0119 |
| **epigallocatechin** | 0.0008 | 0.0004 | 0.001 | 0.0002 | 0.0007 | 0.0002 | 0.1508 | 0.1508 | 0.1508 |
| **epigallocatechin gallate** | 0.0053 | 0.0016 | 0.0062 | 0.0019 | 0.0023 | 0.001 | 0.0833 | 0.8413 | 0.0833 |
| **gallocatechin*** | 0.0009 | 0.0002 | 0.0011 | 0.0003 | 0.0002 | 0.0005 | 0.0238 | 0.4206 | 0.0238 |
| **B1*** | 0.218 | 0.0199 | 0.1742 | 0.0253 | 0.0072 | 0.0409 | 0.0119 | 0.1508 | 0.0119 |
| **B2** | 0.0371 | 0.0046 | 0.0253 | 0.0022 | 0.0025 | 0.0052 | 0.0079 | 0.0079 | 0.0079 |
| **B3*** | 0.0529 | 0.0068 | 0.0516 | 0.0058 | 0.0025 | 0.0176 | 0.0119 | 0.6905 | 0.0119 |
| **B4*** | 0.0227 | 0.0019 | 0.0144 | 0.0068 | 0.0009 | 0.0043 | 0.0119 | 0.0952 | 0.0119 |
| **B1 gallate** | 0.0599 | 0.0296 | 0.0414 | 0.0114 | 0.0268 | 0.007 | 0.1508 | 0.0952 | 0.1508 |
| **C1** | 0.0656 | 0.0084 | 0.0258 | 0.0314 | 0.0046 | 0.0043 | 0.0079 | 0.0079 | 0.0079 |
| **trimer1*** | 0.1066 | 0.0243 | 0.089 | 0.0129 | 0.0082 | 0.0147 | 0.0119 | 0.0952 | 0.0119 |
| *Stilbenes* |  |  |  |  |  |  |  |  |  |
| ***trans*-resveratrol** | 0.0522 | 0.0393 | 0.0449 | 0.0022 | 0.0108 | 0.005 | 0.2222 | 0.0238 | 0.2222 |
| ***trans*-piceid*** | 0.0231 | 0.0081 | 0.0219 | 0.0051 | 0.0036 | 0.0042 | 0.0119 | 0.8413 | 0.0119 |
| ***cis*-piceid*** | 0.0005 | 0.0014 | 0.0007 | 0.0002 | 0.0006 | 0.0002 | 0.0238 | 0.4206 | 0.0238 |
| ***trans*-piceatannol*** | 0.0325 | 0.0156 | 0.0306 | 0.003 | 0.0046 | 0.006 | 0.0238 | 1 | 0.0238 |
| ***trans*-astringin*** | 0.003 | 0.0013 | 0.0028 | 0.0009 | 0.0003 | 0.0006 | 0.0119 | 1 | 0.0119 |
| ***cis*-astringin*** | 0.0121 | 0.0028 | 0.0108 | 0.0021 | 0.0012 | 0.001 | 0.0119 | 0.2222 | 0.0119 |
| ***trans-isorhapotin**** | 0.0005 | 0.0003 | 0.0004 | 0.0002 | 0.0001 | 0.0001 | 0.2262 | 0.6905 | 0.1667 |
| ***trans*-ε-viniferin*** | 0.9964 | 0.7407 | 1.0363 | 0.1147 | 0.1208 | 0.1075 | 0.0476 | 0.6905 | 0.0238 |
| ***trans-*ω-viniferin** | 0.0358 | 0.0374 | 0.0369 | 0.0037 | 0.0072 | 0.0035 | 1 | 1 | 1 |
| ***cis*-ε-viniferin** | 0.0139 | 0.014 | 0.0153 | 0.0053 | 0.0055 | 0.0071 | 1 | 1 | 1 |
| ***trans-*δ*-*viniferin** | 0.0128 | 0.0124 | 0.0135 | 0.0024 | 0.0018 | 0.0021 | 1 | 0.631 | 0.631 |
| **pallidol** | 0.0319 | 0.0441 | 0.0298 | 0.0048 | 0.0111 | 0.0028 | 0.1429 | 0.4206 | 0.0476 |
| **parthenocisin A** | 0.0087 | 0.0142 | 0.0061 | 0.0024 | 0.0053 | 0.0008 | 0.0556 | 0.0476 | 0.0238 |
| **ampelopsin A** | 0.0146 | 0.0159 | 0.0068 | 0.0036 | 0.0279 | 0.0019 | 0.2222 | 0.0238 | 0.2222 |
| **vitisinol C** | 0.0059 | 0.0028 | 0.0046 | 0.0017 | 0.0012 | 0.0014 | 0.0476 | 0.2222 | 0.1429 |
| **dimer diglycoside** | 0.0036 | 0.0035 | 0.0048 | 0.0003 | 0.0012 | 0.0008 | 0.8413 | 0.0238 | 0.1429 |
| **dimer glycoside A** | 0.0023 | 0.0021 | 0.0033 | 0.0004 | 0.0004 | 0.0003 | 0.4206 | 0.0119 | 0.0119 |
| **dimer glycoside B** | 0.0044 | 0.0022 | 0.0057 | 0.0007 | 0.0008 | 0.0007 | 0.0119 | 0.0317 | 0.0119 |
| **dimer glycoside C*** | 0.0058 | 0.0029 | 0.0072 | 0.0009 | 0.0008 | 0.0014 | 0.0119 | 0.1508 | 0.0119 |
| **α-viniferin*** | 0.0523 | 0.1907 | 0.0555 | 0.0114 | 0.0459 | 0.0139 | 0.0119 | 0.6905 | 0.0119 |
| **miyabenol C*** | 0.0303 | 0.1312 | 0.0323 | 0.005 | 0.0377 | 0.0077 | 0.0119 | 1 | 0.0119 |
| **hopeaphenol** | 0.2878 | 0.2377 | 0.4049 | 0.0415 | 0.097 | 0.0513 | 0.3095 | 0.0238 | 0.0238 |
| **isohopeaphenol** | 0.2353 | 0.2607 | 0.3663 | 0.0357 | 0.1095 | 0.0455 | 0.5476 | 0.0238 | 0.0833 |
| **r2-viniferin*** | 0.0137 | 0.0034 | 0.0094 | 0.0025 | 0.001 | 0.0018 | 0.0119 | 0.0556 | 0.0119 |
| **r-viniferin*** | 0.1058 | 0.0335 | 0.0851 | 0.0122 | 0.0063 | 0.0122 | 0.0119 | 0.0556 | 0.0119 |
| *Others* |  |  |  |  |  |  |  |  |  |
| **naringenin** | 0.0004 | 0.0006 | 0.0006 | 0 | 0.0003 | 0.0001 | 0.2262 | 0.0238 | 1 |
| **naringenin glucoside** | 0.0233 | 0.019 | 0.0157 | 0.0039 | 0.0344 | 0.0056 | 0.1508 | 0.1508 | 0.1508 |
| **phloretin*** | 0.0019 | 0.0002 | 0.002 | 0.0003 | 0.0001 | 0.0006 | 0.0119 | 0.8413 | 0.0119 |
| **taxifolin** | 0.0005 | 0.0012 | 0.0008 | 0.0001 | 0.0005 | 0.0002 | 0.1429 | 0.1429 | 0.1508 |

Supplementary table 12: Metabolite concentration, protein content and percentage of water in the scion wood, graft interface and rootstock wood of hetero-grafts of V. vinifera cv. Ugni Blanc onto Vitis berlandieri x Vitis ruprestris cv. 140 Ruggeri, 33 days after grafting. Mean concentrations and standard deviation shown (n = 5). p-value of Wilcoxon-test between scion and interface tissues (S – I), scion and rootstock tissues (S – R), and interface and rootstock tissues (I -R). Stars indicates a significant difference between S – I and between I – R, but no significant difference between S - R. Significance threshold set at p-value < 0.05.

| **Variables** | **Mean** | | | **standard deviation** | | | ***p-value* wilcoxon-test** | | |
| --- | --- | --- | --- | --- | --- | --- | --- | --- | --- |
|  | Scion | Interface | Rootstock | Scion | Interface | Rootstock | S - I | S - R | I - R |
| **% of water** | 62.4765 | 70.2849 | 55.4956 | 1.3318 | 1.0131 | 2.2511 | 0.0079 | 0.0079 | 0.0079 |
| **starch** | 74.328 | 46.9788 | 86.5621 | 21.9209 | 15.2258 | 30.4216 | 0.0833 | 1 | 0.0476 |
| **proteins** | 18.4424 | 30.3092 | 29.4756 | 2.9157 | 4.9176 | 6.8968 | 0.0238 | 0.0476 | 0.8413 |
| **sucrose** | 1.1752 | 1.7239 | 1.9245 | 0.312 | 0.9471 | 0.3937 | 0.4206 | 0.0952 | 0.4206 |
| **glucose** | 1.3014 | 1.8243 | 1.2587 | 0.6279 | 0.3281 | 0.6606 | 0.2262 | 1 | 0.2262 |
| **fructose*** | 1.2533 | 3.3421 | 1.2994 | 0.4285 | 0.3901 | 0.5666 | 0.0119 | 1 | 0.0119 |
| *Amino acids* |  |  |  |  |  |  |  |  |  |
| **Ala*** | 0.4478 | 0.7436 | 0.474 | 0.2211 | 0.1863 | 0.1345 | 0.0833 | 0.5476 | 0.0833 |
| **Arg** | 2.8996 | 3.7375 | 4.7521 | 1.494 | 1.455 | 1.0708 | 0.4206 | 0.2857 | 0.4206 |
| **Asn*** | 2.4043 | 17.1645 | 6.3282 | 2.1562 | 6.2045 | 3.7588 | 0.0238 | 0.0952 | 0.0238 |
| **Asp** | 2.0905 | 3.2206 | 2.6434 | 1.0307 | 0.9041 | 0.9637 | 0.2857 | 0.3095 | 0.3095 |
| **GABA*** | 1.1941 | 2.3585 | 1.4448 | 0.5824 | 0.7176 | 0.4556 | 0.0476 | 0.2222 | 0.0476 |
| **Gln*** | 9.1709 | 21.8433 | 11.9712 | 5.0287 | 6.8677 | 4.1222 | 0.0476 | 0.0952 | 0.0476 |
| **Glu** | 2.7166 | 2.8845 | 3.3274 | 1.2914 | 0.7534 | 1.2486 | 0.6905 | 0.6905 | 0.6905 |
| **Gly** | 0.0173 | 0.0253 | 0.1119 | 0.0249 | 0.0099 | 0.172 | 0.2262 | 0.1667 | 0.5476 |
| **His** | 0.8351 | 0.389 | 0.8026 | 0.4613 | 0.3609 | 0.3444 | 0.313 | 1 | 0.313 |
| **Ile** | 0.2487 | 0.5204 | 0.3077 | 0.1125 | 0.1503 | 0.1204 | 0.0476 | 0.2222 | 0.0833 |
| **Leu** | 0.165 | 0.2268 | 0.2351 | 0.0956 | 0.0691 | 0.0516 | 0.3333 | 0.3333 | 1 |
| **Lys** | 0.1579 | 0.0326 | 0.3447 | 0.0905 | 0.0593 | 0.1969 | 0.0855 | 0.0952 | 0.0335 |
| **Phe** | 0.0797 | 0.0866 | 0.1188 | 0.0443 | 0.0089 | 0.0338 | 0.1508 | 0.1508 | 0.1508 |
| **Pro** | 0.537 | 1.2072 | 0.6254 | 0.2141 | 0.469 | 0.1289 | 0.0476 | 0.2222 | 0.1429 |
| **Ser** | 0.4485 | 1.0135 | 0.7739 | 0.2181 | 0.2419 | 0.341 | 0.0476 | 0.1429 | 0.2222 |
| **Thr** | 0.8356 | 1.2576 | 1.5694 | 0.4104 | 0.409 | 0.4816 | 0.3333 | 0.1667 | 0.4206 |
| **Tyr** | 0.4907 | 0.3971 | 1.0247 | 0.3305 | 0.0981 | 0.2005 | 1 | 0.0833 | 0.0238 |
| **Val** | 0.3209 | 0.6614 | 0.4012 | 0.1731 | 0.198 | 0.1077 | 0.0833 | 0.1508 | 0.0833 |
| *Phenolic acids* |  |  |  |  |  |  |  |  |  |
| **caftaric acid*** | 0.0034 | 0.0004 | 0.0029 | 0.0017 | 0.0003 | 0.0012 | 0.0119 | 0.6905 | 0.0119 |
| **caffeic acid** | 0.0007 | 0.0002 | 0.0008 | 0.0008 | 0.0001 | 0.0001 | 0.0833 | 0.1508 | 0.0238 |
| **gallic acid** | 0.0124 | 0.0191 | 0.0279 | 0.0032 | 0.0064 | 0.0043 | 0.0952 | 0.0238 | 0.0476 |
| **isoferulic acid** | 0.0004 | 0.0007 | 0.0003 | 0.0003 | 0.0002 | 0.0001 | 0.1429 | 0.5476 | 0.0238 |
| *Flavonols* |  |  |  |  |  |  |  |  |  |
| **quercetin 3 glucoside** | 0.0009 | 0.001 | 0.0005 | 0.0002 | 0.0003 | 0.0003 | 0.8413 | 0.1429 | 0.1429 |
| **quercetin 3 glucuronide** | 0.0013 | 0.0015 | 0.0044 | 0.0009 | 0.0009 | 0.0034 | 0.3095 | 0.0833 | 0.0833 |
| **kaempferol 3 glucoside** | 0.0004 | 0.0001 | 0.0001 | 0.0007 | 0 | 0.0001 | 0.631 | 0.631 | 0.8413 |
| *Flavanols* |  |  |  |  |  |  |  |  |  |
| **catechin** | 0.3108 | 0.0519 | 0.1738 | 0.0263 | 0.0048 | 0.0334 | 0.0079 | 0.0079 | 0.0079 |
| **epicatechin** | 0.1306 | 0.006 | 0.0307 | 0.0184 | 0.0011 | 0.0085 | 0.0079 | 0.0079 | 0.0079 |
| **epicatechin gallate** | 0.0499 | 0.0162 | 0.0342 | 0.0055 | 0.0007 | 0.0051 | 0.0079 | 0.0079 | 0.0079 |
| **epigallocatechin** | 0.0006 | 0.0003 | 0.0034 | 0.0001 | 0.0001 | 0.0007 | 0.0079 | 0.0079 | 0.0079 |
| **epigallocatechin gallate** | 0.0011 | 0.0007 | 0.0116 | 0.0004 | 0.0002 | 0.0026 | 0.0556 | 0.0119 | 0.0119 |
| **gallocatechin** | 0.0007 | 0.0001 | 0.0029 | 0.0004 | 0 | 0.0008 | 0.0079 | 0.0079 | 0.0079 |
| **B1** | 0.2495 | 0.0302 | 0.1296 | 0.038 | 0.0071 | 0.0173 | 0.0079 | 0.0079 | 0.0079 |
| **B2** | 0.1102 | 0.0105 | 0.0311 | 0.0163 | 0.0021 | 0.0038 | 0.0079 | 0.0079 | 0.0079 |
| **B3*** | 0.0508 | 0.0075 | 0.0389 | 0.0077 | 0.0015 | 0.0072 | 0.0119 | 0.0556 | 0.0119 |
| **B4** | 0.0272 | 0.0033 | 0.0143 | 0.0046 | 0.001 | 0.0052 | 0.0079 | 0.0079 | 0.0079 |
| **B1 gallate** | 0.0915 | 0.0699 | 0.0894 | 0.0251 | 0.0124 | 0.0432 | 0.2857 | 0.6905 | 0.6905 |
| **C1** | 0.1308 | 0.0203 | 0.0379 | 0.0262 | 0.0045 | 0.0023 | 0.0079 | 0.0079 | 0.0079 |
| **trimer1** | 0.1303 | 0.0432 | 0.0766 | 0.0105 | 0.007 | 0.0178 | 0.0079 | 0.0079 | 0.0079 |
| *Stilbenes* |  |  |  |  |  |  |  |  |  |
| ***trans*-resveratrol** | 0.0388 | 0.0263 | 0.0256 | 0.0062 | 0.0041 | 0.0032 | 0.0119 | 0.0119 | 1 |
| ***trans*-piceid** | 0.024 | 0.0137 | 0.032 | 0.0022 | 0.0024 | 0.004 | 0.0079 | 0.0079 | 0.0079 |
| ***cis*-piceid*** | 0.0012 | 0.0025 | 0.0013 | 0.0002 | 0.0006 | 0.0009 | 0.0238 | 0.5476 | 0.0476 |
| ***trans*-piceatannol** | 0.015 | 0.0128 | 0.0228 | 0.0016 | 0.0032 | 0.0041 | 0.4206 | 0.0119 | 0.0119 |
| ***trans*-astringin** | 0.0012 | 0.0019 | 0.0051 | 0.0003 | 0.0002 | 0.001 | 0.0079 | 0.0079 | 0.0079 |
| ***cis*-astringin** | 0.019 | 0.0039 | 0.0103 | 0.0018 | 0.0008 | 0.0024 | 0.0079 | 0.0079 | 0.0079 |
| ***trans-isorhapotin**** | 0.0004 | 0.0002 | 0.0004 | 0.0001 | 0.0001 | 0.0001 | 0.0833 | 0.5476 | 0.0476 |
| ***trans*-ε-viniferin** | 0.7823 | 0.7495 | 0.6581 | 0.0433 | 0.1176 | 0.0459 | 0.5476 | 0.0238 | 0.3333 |
| ***trans-*ω-viniferin** | 0.0197 | 0.0342 | 0.034 | 0.0034 | 0.0062 | 0.0062 | 0.0119 | 0.0119 | 1 |
| ***cis*-ε-viniferin** | 0.0338 | 0.0279 | 0.0225 | 0.0262 | 0.0149 | 0.0201 | 0.5476 | 0.5476 | 0.5476 |
| ***trans-*δ*-*viniferin** | 0.0101 | 0.0082 | 0.0051 | 0.0015 | 0.0017 | 0.0017 | 0.1508 | 0.0238 | 0.0238 |
| **pallidol*** | 0.0218 | 0.0449 | 0.0227 | 0.004 | 0.0129 | 0.0062 | 0.0238 | 1 | 0.0238 |
| **parthenocisin A*** | 0.0107 | 0.0288 | 0.0094 | 0.0013 | 0.0064 | 0.0015 | 0.0119 | 0.2222 | 0.0119 |
| **ampelopsin A** | 0.1321 | 0.0346 | 0.0164 | 0.0189 | 0.0049 | 0.0056 | 0.0079 | 0.0079 | 0.0079 |
| **vitisinol C** | 0.0064 | 0.0017 | 0.0019 | 0.0011 | 0.0004 | 0.0006 | 0.0119 | 0.0119 | 0.4206 |
| **dimer diglycoside** | 0.003 | 0.0058 | 0.0075 | 0.001 | 0.0014 | 0.0023 | 0.0119 | 0.0119 | 0.2222 |
| **dimer glycoside A** | 0.0014 | 0.0027 | 0.0038 | 0.0002 | 0.0003 | 0.001 | 0.0119 | 0.0119 | 0.0952 |
| **dimer glycoside B** | 0.0072 | 0.0019 | 0.0009 | 0.0007 | 0.0004 | 0.0002 | 0.0079 | 0.0079 | 0.0079 |
| **dimer glycoside C** | 0.0046 | 0.0029 | 0.0022 | 0.0001 | 0.0004 | 0.0004 | 0.0119 | 0.0119 | 0.0159 |
| **α-viniferin** | 0.0734 | 0.1877 | 0.0258 | 0.02 | 0.0229 | 0.0075 | 0.0079 | 0.0079 | 0.0079 |
| **miyabenol C** | 0.0375 | 0.0844 | 0.024 | 0.0065 | 0.0165 | 0.0036 | 0.0119 | 0.0159 | 0.0119 |
| **hopeaphenol** | 0.2074 | 0.1327 | 0.0714 | 0.0391 | 0.0248 | 0.0105 | 0.0159 | 0.0119 | 0.0119 |
| **isohopeaphenol*** | 0.1081 | 0.2651 | 0.1214 | 0.0313 | 0.0778 | 0.029 | 0.0119 | 0.6905 | 0.0119 |
| **r2-viniferin** | 0.0034 | 0.0081 | 0.0279 | 0.0017 | 0.0013 | 0.0057 | 0.0079 | 0.0079 | 0.0079 |
| **r-viniferin** | 0.0342 | 0.0728 | 0.1783 | 0.0029 | 0.0167 | 0.0268 | 0.0079 | 0.0079 | 0.0079 |
| *Others* |  |  |  |  |  |  |  |  |  |
| **naringenin** | 0.0003 | 0.0007 | 0.0004 | 0.0001 | 0.0001 | 0.0001 | 0.0119 | 0.1508 | 0.0119 |
| **naringenin glucoside** | 0.0519 | 0.008 | 0.0226 | 0.0101 | 0.0029 | 0.0237 | 0.0238 | 0.1429 | 1 |
| **phloretin** | 0.0005 | 0.0001 | 0.0012 | 0.0002 | 0 | 0.0001 | 0.0079 | 0.0079 | 0.0079 |
| **taxifolin** | 0.0008 | 0.0016 | 0.0004 | 0.0002 | 0.0001 | 0.0001 | 0.0119 | 0.0317 | 0.0119 |

Supplementary table 13: Metabolite concentration, protein content and percentage of water in the scion wood, graft interface and rootstock wood of hetero-grafts of V. vinifera cv. Ugni Blanc onto Vitis berlandieri x Vitis riparia cv. Rességuier Sélection Birolleau 1, 33 days after grafting. Mean concentrations and standard deviation shown (n = 5). p-value of Wilcoxon-test between scion and interface tissues (S – I), scion and rootstock tissues (S – R), and interface and rootstock tissues (I -R). Stars indicates a significant difference between S – I and between I – R, but no significant difference between S - R. Significance threshold set at p-value < 0.05.

| **Variables** | **Mean** | | | **standard deviation** | | | ***p-value* wilcoxon-test** | | |
| --- | --- | --- | --- | --- | --- | --- | --- | --- | --- |
|  | Scion | Interface | Rootstock | Scion | Interface | Rootstock | S - I | S - R | I - R |
| **% of water** | 62.8157 | 68.7437 | 57.9252 | 1.5159 | 2.4918 | 1.0574 | 0.0159 | 0.0119 | 0.0119 |
| **starch** | 72.1176 | 36.7107 | 46.8772 | 6.8235 | 15.1075 | 6.5976 | 0.0119 | 0.0119 | 0.1508 |
| **proteins** | 20.0988 | 39.0421 | 25.4872 | 0.8648 | 7.6159 | 3.5367 | 0.0159 | 0.0159 | 0.0159 |
| **sucrose** | 1.2822 | 1.9196 | 0.4068 | 0.6056 | 0.5471 | 0.5581 | 0.2222 | 0.0517 | 0.0335 |
| **glucose** | 1.2169 | 2.5677 | 1.75 | 0.6758 | 0.6937 | 0.5053 | 0.0238 | 0.2222 | 0.0833 |
| **fructose*** | 1.3563 | 3.0978 | 1.0135 | 0.2396 | 0.6458 | 1.0308 | 0.0238 | 0.2222 | 0.0238 |
| *Amino acids* |  |  |  |  |  |  |  |  |  |
| **Ala*** | 0.6881 | 1.5202 | 0.3979 | 0.2994 | 0.5592 | 0.0978 | 0.0476 | 0.0952 | 0.0238 |
| **Arg** | 2.963 | 5.8576 | 6.6877 | 0.683 | 1.0082 | 1.6183 | 0.0119 | 0.0119 | 0.6905 |
| **Asn** | 1.4631 | 13.9985 | 7.5093 | 0.3873 | 3.0109 | 1.3171 | 0.0079 | 0.0079 | 0.0079 |
| **Asp*** | 2.407 | 4.7307 | 2.9174 | 0.4391 | 1.0654 | 0.6646 | 0.0238 | 0.2222 | 0.0238 |
| **GABA*** | 1.4427 | 3.6815 | 1.1885 | 0.3482 | 0.9471 | 0.1932 | 0.0119 | 0.1508 | 0.0119 |
| **Gln*** | 8.7535 | 26.1772 | 13.2829 | 2.9639 | 6.8134 | 1.8244 | 0.0119 | 0.0556 | 0.0119 |
| **Glu** | 2.6495 | 3.6974 | 3.4701 | 0.5683 | 0.8328 | 0.7163 | 0.2857 | 0.3333 | 0.8413 |
| **Gly** | 0.2255 | 0.2584 | 0.0746 | 0.1841 | 0.3961 | 0.0885 | 0.5476 | 0.5476 | 0.5476 |
| **His** | 0.9987 | 0.6115 | 1.184 | 0.41 | 0.3245 | 0.405 | 0.4643 | 0.5476 | 0.2857 |
| **Ile*** | 0.3242 | 1.3903 | 0.4816 | 0.1664 | 0.444 | 0.0885 | 0.0119 | 0.3095 | 0.0119 |
| **Leu** | 0.2341 | 0.7154 | 0.5191 | 0.0877 | 0.1989 | 0.0392 | 0.0119 | 0.0119 | 0.0159 |
| **Lys** | 0.2899 | 0.0305 | 0.7547 | 0.148 | 0.0396 | 0.0657 | 0.0119 | 0.0119 | 0.0119 |
| **Phe** | 0.0929 | 0.2466 | 0.1729 | 0.0298 | 0.0701 | 0.0285 | 0.0119 | 0.0119 | 0.0556 |
| **Pro** | 0.5941 | 1.8693 | 0.907 | 0.1298 | 0.6847 | 0.1715 | 0.0238 | 0.0317 | 0.0317 |
| **Ser** | 0.8756 | 2.0823 | 0.5796 | 0.4609 | 1.0919 | 0.1555 | 0.0833 | 0.4206 | 0.0238 |
| **Thr** | 0.9969 | 1.7886 | 2.0877 | 0.3646 | 0.5021 | 0.5036 | 0.0476 | 0.0476 | 0.4206 |
| **Tyr** | 0.3964 | 0.3668 | 0.922 | 0.1049 | 0.1195 | 0.1459 | 0.6905 | 0.0119 | 0.0119 |
| **Val*** | 0.436 | 1.5157 | 0.5777 | 0.1595 | 0.3789 | 0.0808 | 0.0119 | 0.1508 | 0.0119 |
| *Phenolic acids* |  |  |  |  |  |  |  |  |  |
| **caftaric acid*** | 0.0031 | 0.0003 | 0.0035 | 0.0009 | 0.0002 | 0.0006 | 0.0119 | 0.6905 | 0.0119 |
| **caffeic acid** | 0.0002 | 0.0001 | 0.0001 | 0.0001 | 0 | 0.0001 | 0.6667 | 0.8413 | 0.8413 |
| **gallic acid** | 0.0125 | 0.0213 | 0.0233 | 0.0012 | 0.0034 | 0.0034 | 0.0119 | 0.0119 | 0.5476 |
| **isoferulic acid*** | 0.0002 | 0.0007 | 0.0003 | 0.0001 | 0.0002 | 0.0001 | 0.0119 | 0.0952 | 0.0119 |
| *Flavonols* |  |  |  |  |  |  |  |  |  |
| **quercetin 3 glucoside** | 0.0009 | 0.0009 | 0.0002 | 0.0002 | 0.0003 | 0 | 0.5476 | 0.0119 | 0.0119 |
| **quercetin 3 glucuronide** | 0.0005 | 0.0002 | 0.0003 | 0.0002 | 0.0002 | 0.0002 | 0.2262 | 0.2262 | 0.8413 |
| **kaempferol 3 glucoside** | 0.0001 | 0.0001 | 0 | 0 | 0 | 0 | 0.4643 | 0.4524 | 0.6905 |
| *Flavanols* |  |  |  |  |  |  |  |  |  |
| **catechin** | 0.279 | 0.0367 | 0.1677 | 0.0234 | 0.0054 | 0.0244 | 0.0079 | 0.0079 | 0.0079 |
| **epicatechin** | 0.1321 | 0.0066 | 0.0258 | 0.0146 | 0.001 | 0.0045 | 0.0079 | 0.0079 | 0.0079 |
| **epicatechin gallate** | 0.0533 | 0.0126 | 0.0238 | 0.0053 | 0.0035 | 0.0043 | 0.0119 | 0.0119 | 0.0159 |
| **epigallocatechin*** | 0.0007 | 0.0001 | 0.0015 | 0.0001 | 0 | 0.0005 | 0.0119 | 0.1508 | 0.0119 |
| **epigallocatechin gallate** | 0.0009 | 0.0007 | 0.0066 | 0.0003 | 0.0004 | 0.0019 | 0.6905 | 0.0119 | 0.0119 |
| **gallocatechin** | 0.0006 | 0.0002 | 0.0017 | 0.0003 | 0.0002 | 0.0003 | 0.0317 | 0.0119 | 0.0119 |
| **B1** | 0.2635 | 0.0345 | 0.1701 | 0.0126 | 0.0076 | 0.0183 | 0.0079 | 0.0079 | 0.0079 |
| **B2** | 0.1178 | 0.0101 | 0.0234 | 0.0073 | 0.0024 | 0.0031 | 0.0079 | 0.0079 | 0.0079 |
| **B3** | 0.0498 | 0.0079 | 0.0341 | 0.0057 | 0.0031 | 0.0065 | 0.0079 | 0.0079 | 0.0079 |
| **B4** | 0.0269 | 0.0049 | 0.0109 | 0.0027 | 0.001 | 0.0015 | 0.0079 | 0.0079 | 0.0079 |
| **B1 gallate** | 0.0855 | 0.0381 | 0.0376 | 0.0131 | 0.0117 | 0.0052 | 0.0119 | 0.0119 | 1 |
| **C1** | 0.1218 | 0.0237 | 0.0339 | 0.0106 | 0.0168 | 0.0051 | 0.0119 | 0.0119 | 0.5476 |
| **trimer1*** | 0.1598 | 0.0492 | 0.122 | 0.033 | 0.0109 | 0.0251 | 0.0119 | 0.0952 | 0.0119 |
| *Stilbenes* |  |  |  |  |  |  |  |  |  |
| ***trans*-resveratrol** | 0.0307 | 0.019 | 0.0202 | 0.0104 | 0.0088 | 0.005 | 0.1429 | 0.1429 | 0.6905 |
| ***trans*-piceid*** | 0.0189 | 0.0078 | 0.0201 | 0.0021 | 0.0036 | 0.0018 | 0.0119 | 0.3095 | 0.0119 |
| ***cis*-piceid** | 0.0007 | 0.0009 | 0.0004 | 0.0002 | 0.0001 | 0.0001 | 0.2222 | 0.0476 | 0.0238 |
| ***trans*-piceatannol** | 0.0107 | 0.0139 | 0.0167 | 0.0036 | 0.0038 | 0.0047 | 0.3333 | 0.1667 | 0.4206 |
| ***trans*-astringin** | 0.0011 | 0.0016 | 0.0026 | 0.0001 | 0.0006 | 0.0005 | 0.6905 | 0.0238 | 0.0238 |
| ***cis*-astringin** | 0.0168 | 0.0025 | 0.0104 | 0.0014 | 0.0017 | 0.0027 | 0.0079 | 0.0079 | 0.0079 |
| ***trans-isorhapotin**** | 0.0004 | 0.0005 | 0.0006 | 0.0001 | 0.0002 | 0.0002 | 0.4643 | 0.4524 | 0.6905 |
| ***trans*-ε-viniferin** | 0.77 | 0.883 | 1.0412 | 0.0391 | 0.133 | 0.0718 | 0.2222 | 0.0238 | 0.0833 |
| ***trans-*ω-viniferin** | 0.0156 | 0.0339 | 0.0451 | 0.0023 | 0.0053 | 0.0031 | 0.0119 | 0.0119 | 0.0159 |
| ***cis*-ε-viniferin** | 0.0167 | 0.0204 | 0.0216 | 0.0006 | 0.008 | 0.0073 | 1 | 0.4524 | 1 |
| ***trans-*δ*-*viniferin** | 0.0111 | 0.017 | 0.0199 | 0.0019 | 0.0022 | 0.0022 | 0.0238 | 0.0238 | 0.0556 |
| **pallidol** | 0.0226 | 0.063 | 0.0371 | 0.0046 | 0.0105 | 0.005 | 0.0079 | 0.0079 | 0.0079 |
| **parthenocisin A*** | 0.0087 | 0.0219 | 0.0081 | 0.0015 | 0.0053 | 0.0015 | 0.0119 | 0.6905 | 0.0119 |
| **ampelopsin A** | 0.1451 | 0.0341 | 0.0115 | 0.0081 | 0.0074 | 0.0025 | 0.0079 | 0.0079 | 0.0079 |
| **vitisinol C** | 0.0065 | 0.0016 | 0.0021 | 0.0009 | 0.0003 | 0.0004 | 0.0119 | 0.0119 | 0.1508 |
| **dimer diglycoside** | 0.0022 | 0.0038 | 0.0034 | 0.0003 | 0.0005 | 0.0005 | 0.0119 | 0.0119 | 0.2222 |
| **dimer glycoside A** | 0.0012 | 0.0023 | 0.0022 | 0.0003 | 0.0003 | 0.0004 | 0.0119 | 0.0119 | 1 |
| **dimer glycoside B** | 0.0071 | 0.0017 | 0.0015 | 0.0007 | 0.0002 | 0.0004 | 0.0119 | 0.0119 | 0.6905 |
| **dimer glycoside C*** | 0.004 | 0.0026 | 0.0043 | 0.0004 | 0.0005 | 0.0008 | 0.0119 | 0.8413 | 0.0119 |
| **α-viniferin** | 0.0657 | 0.232 | 0.0858 | 0.0104 | 0.0354 | 0.0107 | 0.0119 | 0.0317 | 0.0119 |
| **miyabenol C** | 0.032 | 0.0705 | 0.0237 | 0.0079 | 0.0116 | 0.0032 | 0.0119 | 0.0317 | 0.0119 |
| **hopeaphenol** | 0.1942 | 0.2045 | 0.1687 | 0.0136 | 0.0517 | 0.0487 | 1 | 0.4524 | 0.4643 |
| **isohopeaphenol** | 0.083 | 0.3268 | 0.3245 | 0.0094 | 0.0437 | 0.0582 | 0.0119 | 0.0119 | 0.8413 |
| **r2-viniferin** | 0.0058 | 0.0111 | 0.0275 | 0.0005 | 0.0018 | 0.0049 | 0.0079 | 0.0079 | 0.0079 |
| **r-viniferin** | 0.0379 | 0.1056 | 0.2037 | 0.004 | 0.021 | 0.0386 | 0.0079 | 0.0079 | 0.0079 |
| *Others* |  |  |  |  |  |  |  |  |  |
| **naringenin*** | 0.0002 | 0.0007 | 0.0003 | 0.0001 | 0.0002 | 0 | 0.0119 | 0.3095 | 0.0119 |
| **naringenin glucoside** | 0.0584 | 0.0106 | 0.0096 | 0.0085 | 0.0031 | 0.0014 | 0.0119 | 0.0119 | 0.6905 |
| **phloretin** | 0.0006 | 0.0002 | 0.001 | 0.0001 | 0 | 0.0001 | 0.0079 | 0.0079 | 0.0079 |
| **taxifolin*** | 0.0004 | 0.0014 | 0.0005 | 0.0001 | 0.0002 | 0.0001 | 0.0119 | 0.4206 | 0.0119 |

Supplementary table 14: Metabolite concentration, protein content and percentage of water in the scion wood, graft interface and rootstock wood of hetero-grafts of V. vinifera cv. Ugni Blanc onto Vitis berlandieri x Vitis riparia cv. Sélection Oppenheim 4, 33 days after grafting. Mean concentrations and standard deviation shown (n = 5). p-value of Wilcoxon-test between scion and interface tissues (S – I), scion and rootstock tissues (S – R), and interface and rootstock tissues (I -R). Stars indicates a significant difference between S – I and between I – R, but no significant difference between S - R. Significance threshold set at p-value < 0.05.

| **Variables** | **Mean** | | | **standard deviation** | | | ***p-value* wilcoxon-test** | | |
| --- | --- | --- | --- | --- | --- | --- | --- | --- | --- |
|  | Scion | Interface | Rootstock | Scion | Interface | Rootstock | S - I | S - R | I - R |
| **% of water** | 61.9751 | 69.7455 | 54.9661 | 1.1326 | 1.282 | 1.0948 | 0.0079 | 0.0079 | 0.0079 |
| **starch** | 73.1141 | 47.0258 | 59.1155 | 15.3068 | 8.2913 | 16.4927 | 0.1667 | 0.2222 | 0.2222 |
| **proteins** | 20.5639 | 31.4401 | 28.6346 | 4.617 | 5.947 | 2.8781 | 0.0238 | 0.0238 | 0.3095 |
| **sucrose** | 1.2893 | 2.2384 | 0.7308 | 0.3463 | 0.748 | 0.533 | 0.1429 | 0.2222 | 0.0476 |
| **glucose** | 1.8652 | 2.231 | 1.6776 | 0.2747 | 0.5801 | 0.4447 | 0.6905 | 0.6905 | 0.6667 |
| **fructose*** | 1.6473 | 4.1187 | 1.5559 | 0.4827 | 0.4125 | 0.4267 | 0.0119 | 0.5476 | 0.0119 |
| *Amino acids* |  |  |  |  |  |  |  |  |  |
| **Ala*** | 0.4554 | 0.9747 | 0.3913 | 0.1935 | 0.091 | 0.0668 | 0.0119 | 1 | 0.0119 |
| **Arg** | 2.5415 | 5.4689 | 4.7153 | 0.8146 | 0.7652 | 0.6333 | 0.0119 | 0.0119 | 0.3095 |
| **Asn** | 1.9097 | 22.1107 | 7.7728 | 0.4939 | 5.2883 | 1.5 | 0.0079 | 0.0079 | 0.0079 |
| **Asp** | 1.6746 | 3.3258 | 2.1564 | 0.295 | 0.4172 | 0.2377 | 0.0119 | 0.0317 | 0.0119 |
| **GABA*** | 1.1256 | 2.5442 | 1.2115 | 0.4372 | 0.3677 | 0.2209 | 0.0119 | 0.5476 | 0.0119 |
| **Gln*** | 6.9318 | 25.3816 | 10.264 | 2.2604 | 3.1047 | 1.8746 | 0.0119 | 0.0556 | 0.0119 |
| **Glu** | 2.2072 | 3.0313 | 2.7617 | 0.3673 | 0.2631 | 0.25 | 0.0476 | 0.0476 | 0.0952 |
| **Gly** | 0.0215 | 0.0312 | 0.0378 | 0.0266 | 0.0178 | 0.0235 | 0.5476 | 0.5476 | 0.5476 |
| **His** | 0.5903 | 0.6304 | 0.7194 | 0.3766 | 0.6167 | 0.0776 | 0.6905 | 0.4524 | 0.6905 |
| **Ile** | 0.2539 | 1.5856 | 0.4515 | 0.0361 | 0.1387 | 0.1234 | 0.0119 | 0.0159 | 0.0119 |
| **Leu** | 0.1693 | 0.8406 | 0.4437 | 0.0214 | 0.0993 | 0.1526 | 0.0119 | 0.0159 | 0.0119 |
| **Lys** | 0.0842 | 0.0086 | 0.3021 | 0.0424 | 0.0193 | 0.0118 | 0.0178 | 0.0146 | 0.0146 |
| **Phe** | 0.0624 | 0.1487 | 0.1206 | 0.0179 | 0.0332 | 0.0144 | 0.0119 | 0.0119 | 0.4206 |
| **Pro** | 0.4762 | 1.2533 | 0.734 | 0.0681 | 0.2559 | 0.1565 | 0.0119 | 0.0119 | 0.0159 |
| **Ser*** | 0.3569 | 1.5985 | 0.5274 | 0.1364 | 0.2199 | 0.1532 | 0.0119 | 0.0952 | 0.0119 |
| **Thr** | 0.6442 | 1.6662 | 1.1404 | 0.1555 | 0.21 | 0.049 | 0.0079 | 0.0079 | 0.0079 |
| **Tyr** | 0.3473 | 0.4674 | 0.9066 | 0.0865 | 0.0481 | 0.1097 | 0.0159 | 0.0119 | 0.0119 |
| **Val** | 0.3347 | 1.4146 | 0.5433 | 0.0431 | 0.1414 | 0.1004 | 0.0119 | 0.0159 | 0.0119 |
| *Phenolic acids* |  |  |  |  |  |  |  |  |  |
| **caftaric acid*** | 0.0038 | 0.0004 | 0.0032 | 0.001 | 0.0003 | 0.0011 | 0.0119 | 0.4206 | 0.0119 |
| **caffeic acid** | 0.0008 | 0.0001 | 0.0006 | 0.0011 | 0.0001 | 0.0002 | 0.0833 | 0.2222 | 0.0238 |
| **gallic acid** | 0.0114 | 0.0212 | 0.0247 | 0.0023 | 0.0037 | 0.0063 | 0.0119 | 0.0119 | 0.3095 |
| **isoferulic acid** | 0.0004 | 0.0008 | 0.0004 | 0.0002 | 0.0002 | 0.0001 | 0.0833 | 0.8413 | 0.0238 |
| *Flavonols* |  |  |  |  |  |  |  |  |  |
| **quercetin 3 glucoside** | 0.0011 | 0.0011 | 0.0007 | 0.0004 | 0.0003 | 0.0003 | 1 | 0.2262 | 0.0952 |
| **quercetin 3 glucuronide** | 0.0011 | 0.0004 | 0.002 | 0.0009 | 0.0003 | 0.0015 | 0.1429 | 0.4206 | 0.1429 |
| **kaempferol 3 glucoside** | 0.0001 | 0.0001 | 0.0001 | 0.0001 | 0 | 0.0002 | 1 | 0.631 | 0.631 |
| *Flavanols* |  |  |  |  |  |  |  |  |  |
| **catechin*** | 0.2649 | 0.0514 | 0.2573 | 0.0416 | 0.0106 | 0.0676 | 0.0119 | 1 | 0.0119 |
| **epicatechin** | 0.1099 | 0.0066 | 0.0373 | 0.0209 | 0.0019 | 0.0102 | 0.0079 | 0.0079 | 0.0079 |
| **epicatechin gallate** | 0.0546 | 0.0185 | 0.0386 | 0.011 | 0.0034 | 0.0055 | 0.0119 | 0.0317 | 0.0119 |
| **epigallocatechin** | 0.0004 | 0.0002 | 0.0015 | 0.0002 | 0.0001 | 0.0005 | 0.1508 | 0.0119 | 0.0119 |
| **epigallocatechin gallate** | 0.0008 | 0.0008 | 0.0072 | 0.0001 | 0.0004 | 0.0025 | 0.1508 | 0.0119 | 0.0119 |
| **gallocatechin** | 0.0006 | 0.0002 | 0.0016 | 0.0003 | 0.0002 | 0.001 | 0.0317 | 0.0317 | 0.0238 |
| **B1*** | 0.2099 | 0.0303 | 0.1599 | 0.0496 | 0.0084 | 0.0413 | 0.0119 | 0.2222 | 0.0119 |
| **B2** | 0.0909 | 0.008 | 0.023 | 0.0196 | 0.002 | 0.0093 | 0.0079 | 0.0079 | 0.0079 |
| **B3*** | 0.0403 | 0.0097 | 0.0462 | 0.0074 | 0.0024 | 0.0124 | 0.0119 | 0.4206 | 0.0119 |
| **B4*** | 0.0248 | 0.0026 | 0.016 | 0.0087 | 0.0006 | 0.0037 | 0.0119 | 0.0952 | 0.0119 |
| **B1 gallate** | 0.0862 | 0.0445 | 0.05 | 0.0249 | 0.0162 | 0.0151 | 0.0476 | 0.0476 | 0.8413 |
| **C1** | 0.1124 | 0.0173 | 0.0278 | 0.0284 | 0.0048 | 0.0141 | 0.0119 | 0.0119 | 0.0952 |
| **trimer1*** | 0.1168 | 0.0361 | 0.0814 | 0.0248 | 0.0082 | 0.0172 | 0.0119 | 0.0952 | 0.0119 |
| *Stilbenes* |  |  |  |  |  |  |  |  |  |
| ***trans*-resveratrol** | 0.0598 | 0.0347 | 0.0335 | 0.0378 | 0.0105 | 0.0087 | 0.4643 | 0.4643 | 1 |
| ***trans*-piceid** | 0.0225 | 0.0125 | 0.0183 | 0.0058 | 0.003 | 0.004 | 0.0476 | 0.2222 | 0.0833 |
| ***cis*-piceid** | 0.0013 | 0.0023 | 0.0007 | 0.0005 | 0.0006 | 0.0003 | 0.0556 | 0.0556 | 0.0238 |
| ***trans*-piceatannol** | 0.0214 | 0.0197 | 0.0293 | 0.007 | 0.0038 | 0.0036 | 0.8413 | 0.3333 | 0.0238 |
| ***trans*-astringin** | 0.001 | 0.0016 | 0.0021 | 0.0002 | 0.0002 | 0.0001 | 0.0159 | 0.0119 | 0.0119 |
| ***cis*-astringin** | 0.0161 | 0.0042 | 0.0117 | 0.0028 | 0.0015 | 0.0011 | 0.0119 | 0.0159 | 0.0119 |
| ***trans-isorhapotin**** | 0.0004 | 0.0004 | 0.0004 | 0.0001 | 0 | 0.0001 | 1 | 0.9286 | 1 |
| ***trans*-ε-viniferin** | 0.8186 | 0.9317 | 1.0642 | 0.1087 | 0.0693 | 0.1469 | 0.0476 | 0.0476 | 0.2222 |
| ***trans-*ω-viniferin** | 0.0296 | 0.0402 | 0.0413 | 0.017 | 0.004 | 0.0032 | 0.2262 | 0.2262 | 1 |
| ***cis*-ε-viniferin** | 0.0275 | 0.0171 | 0.0244 | 0.0236 | 0.0045 | 0.0106 | 0.6905 | 0.6905 | 0.6667 |
| ***trans-*δ*-*viniferin** | 0.0116 | 0.0143 | 0.0165 | 0.0026 | 0.0022 | 0.0062 | 0.2262 | 0.2262 | 0.6905 |
| **pallidol*** | 0.0328 | 0.0509 | 0.0307 | 0.0108 | 0.0066 | 0.0058 | 0.0119 | 0.5476 | 0.0119 |
| **parthenocisin A*** | 0.0157 | 0.0222 | 0.0102 | 0.0041 | 0.0014 | 0.0028 | 0.0119 | 0.0556 | 0.0119 |
| **ampelopsin A** | 0.1494 | 0.0377 | 0.0128 | 0.0352 | 0.0071 | 0.004 | 0.0079 | 0.0079 | 0.0079 |
| **vitisinol C** | 0.0066 | 0.0025 | 0.0033 | 0.0013 | 0.0006 | 0.001 | 0.0238 | 0.0238 | 0.2222 |
| **dimer diglycoside** | 0.0028 | 0.0052 | 0.0065 | 0.0005 | 0.0013 | 0.0016 | 0.0119 | 0.0119 | 0.0952 |
| **dimer glycoside A** | 0.0014 | 0.0027 | 0.0032 | 0.0002 | 0.0004 | 0.0003 | 0.0119 | 0.0119 | 0.1508 |
| **dimer glycoside B** | 0.0072 | 0.0031 | 0.0044 | 0.0011 | 0.0006 | 0.0011 | 0.0119 | 0.0119 | 0.0952 |
| **dimer glycoside C** | 0.005 | 0.0039 | 0.006 | 0.0008 | 0.0005 | 0.0012 | 0.0833 | 0.2222 | 0.0476 |
| **α-viniferin*** | 0.1052 | 0.2382 | 0.0786 | 0.0335 | 0.0518 | 0.0284 | 0.0119 | 0.3095 | 0.0119 |
| **miyabenol C*** | 0.056 | 0.1371 | 0.0418 | 0.0191 | 0.0149 | 0.0103 | 0.0119 | 0.3095 | 0.0119 |
| **hopeaphenol** | 0.231 | 0.291 | 0.3682 | 0.0559 | 0.0662 | 0.1203 | 0.3333 | 0.1667 | 0.4206 |
| **isohopeaphenol** | 0.1293 | 0.3597 | 0.4247 | 0.036 | 0.0385 | 0.0988 | 0.0119 | 0.0119 | 0.2222 |
| **r2-viniferin** | 0.0047 | 0.0043 | 0.0151 | 0.002 | 0.0007 | 0.0085 | 0.5476 | 0.0119 | 0.0119 |
| **r-viniferin** | 0.0284 | 0.0473 | 0.1219 | 0.0144 | 0.0106 | 0.0467 | 0.0556 | 0.0119 | 0.0119 |
| *Others* |  |  |  |  |  |  |  |  |  |
| **naringenin*** | 0.0003 | 0.0007 | 0.0004 | 0.0001 | 0.0001 | 0.0001 | 0.0119 | 0.4206 | 0.0119 |
| **naringenin glucoside** | 0.0588 | 0.0087 | 0.0131 | 0.0136 | 0.0034 | 0.0043 | 0.0119 | 0.0119 | 0.1508 |
| **phloretin** | 0.0007 | 0.0003 | 0.0016 | 0.0003 | 0.0001 | 0.0004 | 0.0119 | 0.0159 | 0.0119 |
| **taxifolin*** | 0.0007 | 0.0015 | 0.0007 | 0.0004 | 0.0001 | 0.0001 | 0.0119 | 0.6905 | 0.0119 |

Supplementary table 15: Metabolite concentration, protein content and percentage of water in the scion wood, graft interface and rootstock wood of homo-grafts of V. vinifera cv. Ugni Blanc 33 days after grafting. Mean concentrations and standard deviation shown (n = 5). p-value of Wilcoxon-test between scion and interface tissues (S – I), scion and rootstock tissues (S – R), and interface and rootstock tissues (I -R). Stars indicates a significant difference between S – I and between I – R, but no significant difference between S - R. Significance threshold set at p-value < 0.05.

| **Variables** | **Mean** | | | **standard deviation** | | | ***p-value* wilcoxon-test** | | |
| --- | --- | --- | --- | --- | --- | --- | --- | --- | --- |
|  | Scion | Interface | Rootstock | Scion | Interface | Rootstock | S - I | S - R | I - R |
| **% of water*** | 61.0788 | 74.5631 | 62.4345 | 1.8008 | 2.4204 | 1.7151 | 0.0119 | 0.4206 | 0.0119 |
| **starch** | 62.5644 | 70.5563 | 49.1126 | 27.2466 | 23.4745 | 8.9306 | 0.6905 | 0.6905 | 0.2857 |
| **proteins*** | 22.7207 | 25.8968 | 18.4548 | 1.7 | 1.5657 | 5.5024 | 0.0238 | 0.1508 | 0.0238 |
| **sucrose** | 1.269 | 1.7676 | 0.8918 | 0.1937 | 0.5816 | 0.1376 | 0.3095 | 0.0476 | 0.0238 |
| **glucose** | 0.7977 | 1.3671 | 0.5745 | 0.5783 | 0.3346 | 0.5742 | 0.1429 | 0.6004 | 0.1429 |
| **fructose*** | 0.9303 | 1.877 | 0.5648 | 0.3697 | 0.1999 | 0.2439 | 0.0119 | 0.0952 | 0.0119 |
| *Amino acids* |  |  |  |  |  |  |  |  |  |
| **Ala** | 0.3781 | 0.7377 | 0.4581 | 0.2295 | 0.1556 | 0.1 | 0.0833 | 0.4206 | 0.0476 |
| **Arg** | 2.8379 | 7.874 | 10.0031 | 1.2938 | 5.5107 | 2.444 | 0.0476 | 0.0238 | 0.2222 |
| **Asn** | 1.0854 | 6.7663 | 2.7785 | 0.5397 | 1.8989 | 0.3528 | 0.0079 | 0.0079 | 0.0079 |
| **Asp** | 2.1647 | 3.1445 | 2.684 | 0.6783 | 0.6703 | 0.4561 | 0.2857 | 0.3095 | 0.3095 |
| **GABA** | 0.8697 | 1.744 | 1.3983 | 0.3271 | 0.278 | 0.3512 | 0.0238 | 0.0833 | 0.1508 |
| **Gln** | 7.4548 | 22.0053 | 14.3291 | 2.6173 | 3.6336 | 2.3783 | 0.0079 | 0.0079 | 0.0079 |
| **Glu** | 2.8147 | 3.2347 | 3.4963 | 0.8782 | 1.3983 | 0.6052 | 0.8413 | 0.4643 | 0.4643 |
| **Gly** | 0.0381 | 0.0514 | 0.026 | 0.0543 | 0.041 | 0.0317 | 0.7944 | 0.8325 | 0.6667 |
| **His** | 0.3875 | 0.655 | 1.2222 | 0.5201 | 1.1324 | 0.4198 | 0.9166 | 0.1667 | 0.2262 |
| **Ile*** | 0.1782 | 0.3256 | 0.2179 | 0.0715 | 0.0653 | 0.0249 | 0.0238 | 0.2222 | 0.0238 |
| **Leu** | 0.1314 | 0.1704 | 0.1673 | 0.0495 | 0.0641 | 0.0132 | 0.631 | 0.4524 | 1 |
| **Lys** | 0.1685 | 0.2221 | 0.4585 | 0.146 | 0.3949 | 0.16 | 0.5296 | 0.0476 | 0.2137 |
| **Phe** | 0.0576 | 0.065 | 0.0532 | 0.0285 | 0.0361 | 0.015 | 1 | 1 | 1 |
| **Pro** | 0.5141 | 0.7989 | 0.6424 | 0.0807 | 0.102 | 0.1494 | 0.0238 | 0.2222 | 0.2222 |
| **Ser** | 0.4698 | 1.051 | 0.5249 | 0.348 | 0.2687 | 0.154 | 0.0833 | 0.4206 | 0.0476 |
| **Thr** | 0.743 | 1.891 | 2.183 | 0.3254 | 1.5002 | 0.5262 | 0.0476 | 0.0238 | 0.1508 |
| **Tyr** | 0.1851 | 0.2961 | 0.456 | 0.0726 | 0.3267 | 0.076 | 1 | 0.0238 | 0.2262 |
| **Val*** | 0.2831 | 0.4591 | 0.2968 | 0.1049 | 0.1145 | 0.0404 | 0.0476 | 0.5476 | 0.0476 |
| *Phenolic acids* |  |  |  |  |  |  |  |  |  |
| **caftaric acid** | 0.0024 | 0.0002 | 0.0002 | 0.0006 | 0.0001 | 0 | 0.0119 | 0.0119 | 0.5476 |
| **caffeic acid** | 0.0003 | 0.0001 | 0.0003 | 0.0002 | 0 | 0.0003 | 0.0238 | 0.8413 | 0.3333 |
| **gallic acid** | 0.0121 | 0.0085 | 0.0085 | 0.0013 | 0.0013 | 0.0017 | 0.0238 | 0.0238 | 1 |
| **isoferulic acid** | 0.0003 | 0.0003 | 0.0005 | 0.0001 | 0 | 0.0002 | 0.6905 | 0.0119 | 0.0119 |
| *Flavonols* |  |  |  |  |  |  |  |  |  |
| **quercetin 3 glucoside*** | 0.0013 | 0.0022 | 0.0011 | 0.0004 | 0.0003 | 0.0004 | 0.0238 | 0.4206 | 0.0238 |
| **quercetin 3 glucuronide** | 0.0009 | 0.0003 | 0.0006 | 0.0004 | 0.0001 | 0.0004 | 0.0476 | 0.5476 | 0.5476 |
| **kaempferol 3 glucoside*** | 0.0001 | 0.0003 | 0.0001 | 0.0001 | 0.0001 | 0 | 0.0238 | 0.5476 | 0.0238 |
| *Flavanols* |  |  |  |  |  |  |  |  |  |
| **catechin*** | 0.2333 | 0.078 | 0.2686 | 0.0191 | 0.0123 | 0.0368 | 0.0119 | 0.0952 | 0.0119 |
| **epicatechin** | 0.0991 | 0.0083 | 0.037 | 0.0115 | 0.0021 | 0.0046 | 0.0079 | 0.0079 | 0.0079 |
| **epicatechin gallate** | 0.0485 | 0.0211 | 0.0267 | 0.0092 | 0.0033 | 0.0017 | 0.0079 | 0.0079 | 0.0079 |
| **epigallocatechin** | 0.0004 | 0.0001 | 0.0001 | 0.0003 | 0.0001 | 0.0001 | 0.0952 | 0.1429 | 0.2222 |
| **epigallocatechin gallate** | 0.0005 | 0.0002 | 0.0004 | 0.0002 | 0.0001 | 0.0001 | 0.0833 | 0.6905 | 0.0833 |
| **gallocatechin** | 0.0005 | 0 | 0.0002 | 0.0001 | 0 | 0.0002 | 0.0238 | 0.0317 | 0.0317 |
| **B1** | 0.2009 | 0.0425 | 0.1073 | 0.0311 | 0.0078 | 0.0174 | 0.0079 | 0.0079 | 0.0079 |
| **B2** | 0.0899 | 0.0155 | 0.0293 | 0.0134 | 0.0027 | 0.0045 | 0.0079 | 0.0079 | 0.0079 |
| **B3** | 0.0381 | 0.0089 | 0.022 | 0.0085 | 0.0019 | 0.0032 | 0.0079 | 0.0079 | 0.0079 |
| **B4*** | 0.0338 | 0.0043 | 0.0156 | 0.0251 | 0.001 | 0.0116 | 0.0119 | 0.3095 | 0.0119 |
| **B1 gallate** | 0.0729 | 0.0531 | 0.0274 | 0.0129 | 0.0046 | 0.0046 | 0.0079 | 0.0079 | 0.0079 |
| **C1** | 0.1095 | 0.0253 | 0.0344 | 0.0179 | 0.0037 | 0.0064 | 0.0119 | 0.0119 | 0.0317 |
| **trimer1** | 0.1238 | 0.0543 | 0.0506 | 0.0184 | 0.0121 | 0.0074 | 0.0119 | 0.0119 | 1 |
| *Stilbenes* |  |  |  |  |  |  |  |  |  |
| ***trans*-resveratrol** | 0.0301 | 0.0148 | 0.0131 | 0.0082 | 0.0039 | 0.0019 | 0.0119 | 0.0119 | 0.3095 |
| ***trans*-piceid** | 0.0199 | 0.005 | 0.0102 | 0.0028 | 0.0017 | 0.0017 | 0.0079 | 0.0079 | 0.0079 |
| ***cis*-piceid*** | 0.0007 | 0.0016 | 0.0005 | 0.0002 | 0.0006 | 0.0002 | 0.0119 | 0.0952 | 0.0119 |
| ***trans*-piceatannol** | 0.0135 | 0.011 | 0.013 | 0.0038 | 0.0039 | 0.0021 | 0.631 | 0.8413 | 0.631 |
| ***trans*-astringin** | 0.0016 | 0.0015 | 0.0023 | 0.0003 | 0.0004 | 0.0003 | 0.8413 | 0.0238 | 0.0238 |
| ***cis*-astringin** | 0.0169 | 0.0029 | 0.0073 | 0.0017 | 0.0017 | 0.0023 | 0.0119 | 0.0119 | 0.0159 |
| ***trans-isorhapotin**** | 0.0005 | 0.0006 | 0.0006 | 0.0001 | 0.0002 | 0.0003 | 0.4524 | 0.631 | 0.8413 |
| ***trans*-ε-viniferin** | 0.7832 | 0.7078 | 0.6751 | 0.027 | 0.0613 | 0.0489 | 0.0833 | 0.0238 | 0.4206 |
| ***trans-*ω-viniferin*** | 0.0194 | 0.0264 | 0.0174 | 0.003 | 0.0036 | 0.0024 | 0.0476 | 0.3095 | 0.0238 |
| ***cis*-ε-viniferin** | 0.0156 | 0.0163 | 0.0235 | 0.0064 | 0.0025 | 0.0078 | 1 | 0.2262 | 0.2262 |
| ***trans-*δ*-*viniferin** | 0.0133 | 0.01 | 0.0119 | 0.0021 | 0.0014 | 0.0015 | 0.0476 | 0.4206 | 0.1429 |
| **pallidol** | 0.0286 | 0.0353 | 0.0217 | 0.0048 | 0.0051 | 0.0039 | 0.1508 | 0.0476 | 0.0238 |
| **parthenocisin A*** | 0.0105 | 0.0257 | 0.011 | 0.0018 | 0.0044 | 0.0034 | 0.0119 | 0.8413 | 0.0119 |
| **ampelopsin A** | 0.1512 | 0.0719 | 0.109 | 0.0066 | 0.0096 | 0.0037 | 0.0079 | 0.0079 | 0.0079 |
| **vitisinol C*** | 0.0074 | 0.0033 | 0.0058 | 0.0012 | 0.0006 | 0.0012 | 0.0119 | 0.0556 | 0.0119 |
| **dimer diglycoside** | 0.0022 | 0.003 | 0.0016 | 0.0002 | 0.0005 | 0.0003 | 0.0159 | 0.0159 | 0.0159 |
| **dimer glycoside A*** | 0.0014 | 0.0017 | 0.001 | 0.0002 | 0.0001 | 0.0003 | 0.0238 | 0.0556 | 0.0238 |
| **dimer glycoside B** | 0.0074 | 0.0036 | 0.0045 | 0.0007 | 0.0005 | 0.0004 | 0.0119 | 0.0119 | 0.0317 |
| **dimer glycoside C** | 0.0043 | 0.0032 | 0.0031 | 0.0003 | 0.0003 | 0.0003 | 0.0119 | 0.0119 | 0.6905 |
| **α-viniferin*** | 0.0949 | 0.2259 | 0.1024 | 0.0198 | 0.0398 | 0.0218 | 0.0119 | 0.6905 | 0.0119 |
| **miyabenol C** | 0.0479 | 0.0648 | 0.0312 | 0.0132 | 0.0063 | 0.0064 | 0.0556 | 0.0556 | 0.0238 |
| **hopeaphenol** | 0.2321 | 0.148 | 0.1713 | 0.0288 | 0.0312 | 0.0239 | 0.0119 | 0.0119 | 0.3095 |
| **isohopeaphenol*** | 0.1155 | 0.1634 | 0.1053 | 0.0311 | 0.0273 | 0.0278 | 0.0476 | 0.6905 | 0.0476 |
| **r2-viniferin*** | 0.0045 | 0.002 | 0.0039 | 0.0008 | 0.0003 | 0.0008 | 0.0119 | 0.2222 | 0.0119 |
| **r-viniferin** | 0.0221 | 0.0163 | 0.0173 | 0.017 | 0.0087 | 0.0123 | 0.5476 | 0.5476 | 0.5476 |
| *Others* |  |  |  |  |  |  |  |  |  |
| **naringenin** | 0.0003 | 0.0001 | 0.0002 | 0.0001 | 0 | 0.0001 | 0.0476 | 0.4206 | 0.3333 |
| **naringenin glucoside** | 0.0495 | 0.0542 | 0.0388 | 0.0053 | 0.0083 | 0.0063 | 0.5476 | 0.0476 | 0.0238 |
| **phloretin** | 0.0005 | 0.0001 | 0.0002 | 0.0001 | 0.0001 | 0.0001 | 0.0159 | 0.0159 | 0.0159 |
| **taxifolin** | 0.0007 | 0.0003 | 0.0004 | 0.0003 | 0 | 0.0001 | 0.0238 | 0.0476 | 0.0952 |

Supplementary table 16: Mass of precursor ion (*Precursor Ion*), mass of one of the fragments used for quantification (*Quantifier*), retention time (*Rt*) and ion polarity for the 49 polyphenols for HPLC-QqQ in MRM mode.

|  | *Precusor Ion* | *Quantifier* | *Rt (min)* | | *Ion Polarity* |  |
| --- | --- | --- | --- | --- | --- | --- |
| *Phenolic acids* |  |  |  |  | | |
| caftaric acid | 311 | 149 | 6.21 | Negative | | |
| caffeic acid | 181 | 163 | 9.138 | Positive | | |
| gallic acid | 169 | 125 | 2.372 | Negative | | |
| isoferulic acid | 195 | 177.1 | 11.8 | Positive | | |
| *Flavonols* |  |  |  |  | | |
| quercetin 3 glucoside* | 465 | 303 | 12 | Positive | | |
| quercetin 3 glucuronide | 479 | 303 | 11.869 | Positive | | |
| kaempferol 3 glucoside* | 449 | 287 | 12.826 | Positive | | |
| *Flavanols* |  |  |  |  | | |
| catechin* | 291 | 139 | 8.4 | Positive | | |
| epicatechin | 291 | 139 | 9.8 | Positive | | |
| epicatechin gallate | 443 | 123.1 | 11.855 | Positive | | |
| epigallocatechin | 307 | 139.1 | 7.806 | Positive | | |
| epigallocatechin gallate | 459 | 139 | 9.908 | Positive | | |
| gallocatechin | 307 | 151.1 | 7.822 | Positive | | |
| B1 | 577 | 289 | 7.586 | Negative | | |
| B2 | 577 | 289 | 9.15 | Negative | | |
| B3 | 577 | 289 | 7.91 | Negative | | |
| B4* | 577 | 289 | 8.7 | Negative | | |
| B1 gallate | 731 | 127 | 10.5 | Positive | | |
| C1 | 865 | 289.1 | 10.193 | Negative | | |
| trimer1 | 865 | 289.1 | 8.6 | Negative | | |
| *Stilbenes* |  |  |  |  | | |
| *trans*-resveratrol | 229 | 135 | 14.515 | Positive | | |
| *trans*-piceid | 389 | 227 | 11.66 | Negative | | |
| *cis*-piceid* | 389 | 227 | 13.403 | Negative | | |
| *trans*-piceatannol | 245 | 135 | 12.7 | Positive | | |
| *trans*-astringin | 405 | 243 | 10.292 | Negative | | |
| *cis*-astringin | 407 | 245 | 12.18 | Positive | | |
| *trans-isorhapotin** | 419 | 257 | 12.237 | Negative | | |
| *trans*-ε-viniferin | 455 | 361 | 17.184 | Positive | | |
| *trans-*ω-viniferin* | 455 | 361 | 17.9 | Positive | | |
| *cis*-ε-viniferin | 455 | 361 | 16.779 | Positive | | |
| *trans-*δ*-*viniferin | 455 | 361 | 18.5 | Positive | | |
| pallidol | 455 | 361 | 13.7 | Positive | | |
| parthenocisin A* | 455 | 361 | 14.65 | Positive | | |
| ampelopsin A | 471 | 453 | 12.469 | Positive | | |
| vitisinol C* | 429 | 107 | 17.201 | Positive | | |
| dimer diglycoside | 777 | 615 | 14.55 | Negative | | |
| dimer glycoside A* | 615 | 453 | 13.3 | Negative | | |
| dimer glycoside B | 615 | 453 | 13.6 | Negative | | |
| dimer glycoside C | 615 | 453 | 15.036 | Negative | | |
| α-viniferin* | 679 | 359 | 18.138 | Positive | | |
| miyabenol C | 681 | 575 | 17.523 | Positive | | |
| hopeaphenol | 908 | 359 | 15.95 | Positive | | |
| isohopeaphenol* | 908 | 359 | 16.25 | Positive | | |
| r2-viniferin* | 908 | 559 | 17.81 | Positive | | |
| r-viniferin | 908 | 559 | 19.28 | Positive | | |
| *Others* |  |  |  |  | | |
| naringenin | 271 | 119.1 | 17.39 | Negative | | |
| naringenin glucoside | 435 | 273 | 12.956 | Positive | | |
| phloretin | 275 | 107 | 13.866 | Positive | | |
| taxifolin | 303 | 285 | 12.189 | Negative | | |
