# Supplementary figures and images for: Identifying early metabolite markers of successful graft union formation in grapevine
Source: Hortic Res. 2022 Jan 19;9:uhab070. doi: 10.1093/hr/uhab070 (PMC8881376; doi:10.1093/hr/uhab070)

Color Key  
and Density Plot

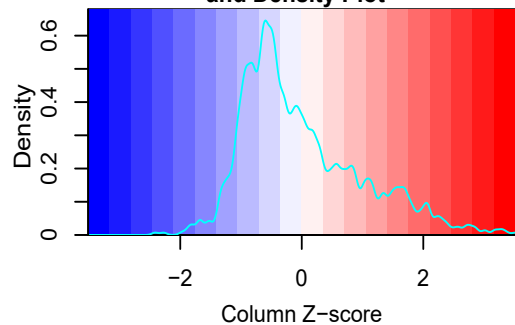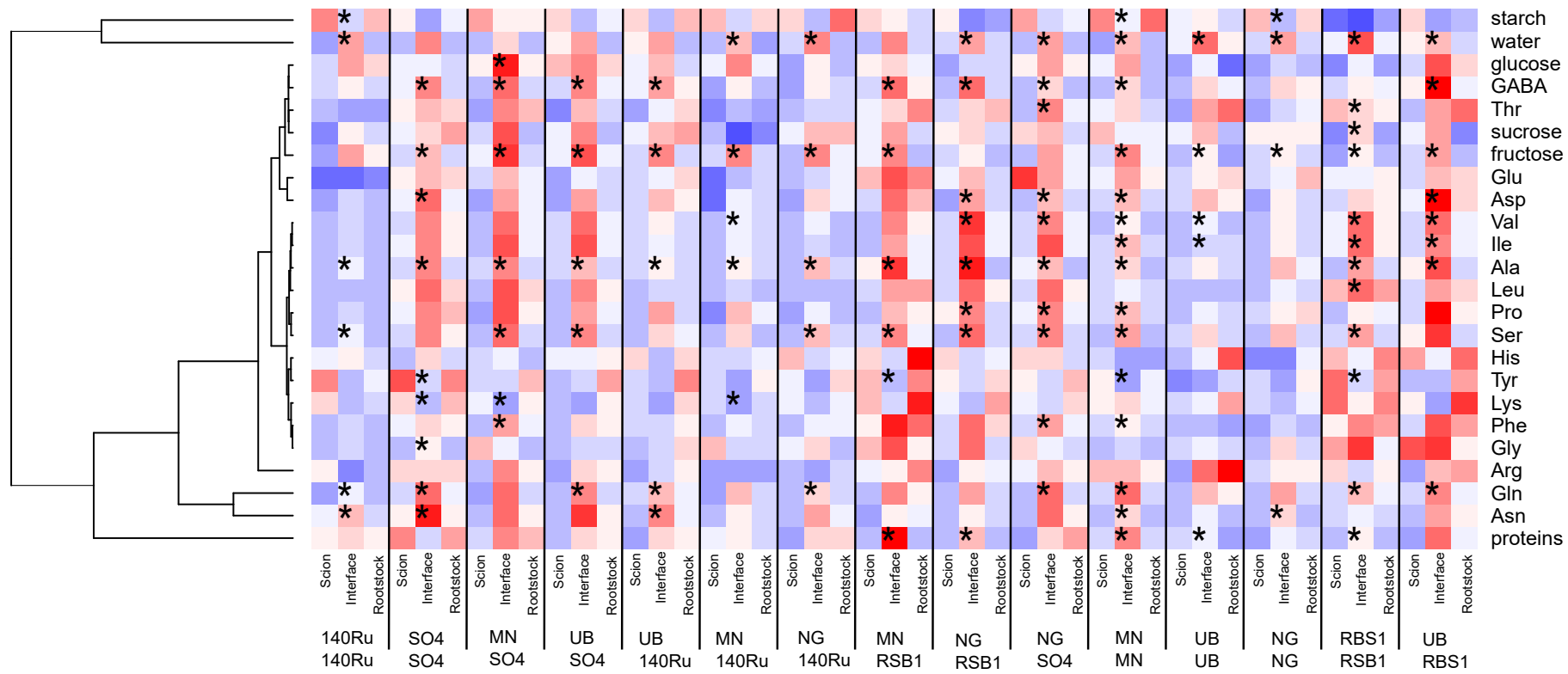

Supplement: Web_Material_uhab070 [file web_material_uhab070.zip › supplementary figure 1.pdf]

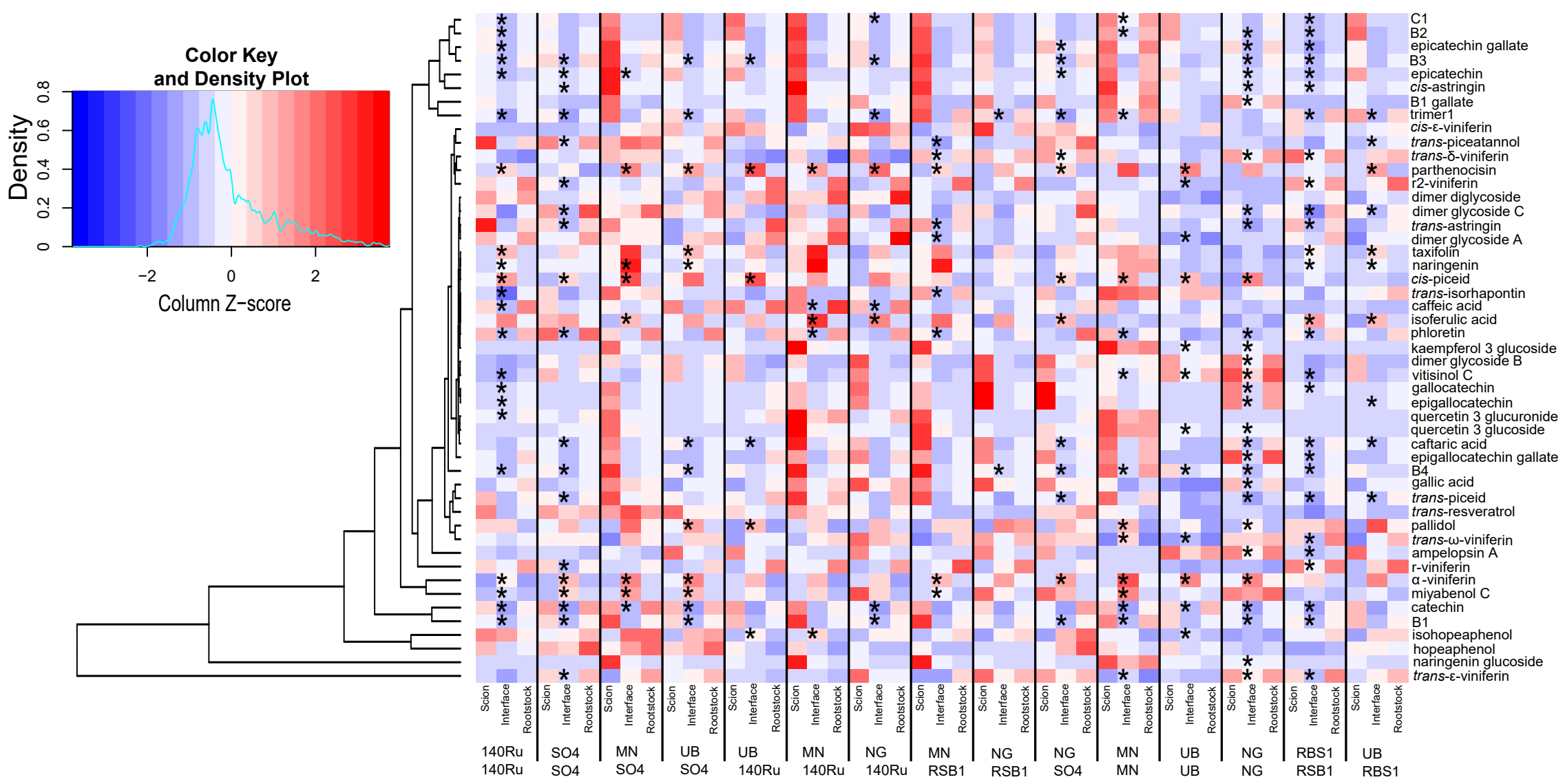

Supplement: Web_Material_uhab070 [file web_material_uhab070.zip › supplementary figure 2.pdf]
